# Heme oxygenase‐1 deficiency triggers exhaustion of hematopoietic stem cells

**DOI:** 10.15252/embr.201947895

**Published:** 2019-12-29

**Authors:** Krzysztof Szade, Monika Zukowska, Agata Szade, Witold Nowak, Izabella Skulimowska, Maciej Ciesla, Karolina Bukowska‐Strakova, Gunsagar Singh Gulati, Neli Kachamakova‐Trojanowska, Anna Kusienicka, Elisa Einwallner, Jacek Kijowski, Szymon Czauderna, Harald Esterbauer, Vladimir Benes, Irving L Weissman, Jozef Dulak, Alicja Jozkowicz

**Affiliations:** ^1^ Department of Medical Biotechnology Faculty of Biochemistry, Biophysics and Biotechnology Jagiellonian University Krakow Poland; ^2^ Institute for Stem Cell Biology and Regenerative Medicine Stanford University Stanford CA USA; ^3^ Department of Clinical Immunology Institute of Pediatrics Jagiellonian University Medical College Krakow Poland; ^4^ Malopolska Centre of Biotechnology Jagiellonian University Krakow Poland; ^5^ Department of Laboratory Medicine Center of Translational Research Medical University of Vienna Vienna Austria; ^6^ Department of Transplantation Institute of Pediatrics Jagiellonian University Medical College Krakow Poland; ^7^ Genomics Core Facility EMBL Heidelberg Heidelberg Germany

**Keywords:** aging, bone marrow, cxcl12‐abudant reticular cells, endothelial cells, niche, Haematology, Regenerative Medicine

## Abstract

While intrinsic changes in aging hematopoietic stem cells (HSCs) are well characterized, it remains unclear how extrinsic factors affect HSC aging. Here, we demonstrate that cells in the niche—endothelial cells (ECs) and CXCL12‐abundant reticular cells (CARs)—highly express the heme‐degrading enzyme, heme oxygenase 1 (HO‐1), but then decrease its expression with age. HO‐1‐deficient animals (HO‐1^−/−^) have altered numbers of ECs and CARs that produce less hematopoietic factors. HSCs co‐cultured *in vitro* with HO‐1^−/−^ mesenchymal stromal cells expand, but have altered kinetic of growth and differentiation of derived colonies. HSCs from young HO‐1^−/−^ animals have reduced quiescence and regenerative potential. Young HO‐1^−/−^ HSCs exhibit features of premature exhaustion on the transcriptional and functional level. HO‐1^+/+^ HSCs transplanted into HO‐1^−/−^ recipients exhaust their regenerative potential early and do not reconstitute secondary recipients. In turn, transplantation of HO‐1^−/−^ HSCs to the HO‐1^+/+^ recipients recovers the regenerative potential of HO‐1^−/−^ HSCs and reverses their transcriptional alterations. Thus, HSC‐extrinsic activity of HO‐1 prevents HSCs from premature exhaustion and may restore the function of aged HSCs.

## Graphical abstract

The bone marrow niche harbors cell populations that constitutively express heme oxygenase 1 (HO‐1). HO‐1 regulates the niche and protects HSCs from exhaustion in a cell extrinsic manner.

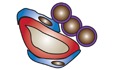

## Introduction

Hematopoietic stem cells (HSCs) maintain the production of all blood cells throughout life, but their functional status changes with age 1, 2, 3, 4. As shown both in mouse and human, aged HSCs expand their pool; however, their regenerative potential is impaired 2, 3, 4, 5. HSCs isolated from old mice and old humans reconstitute hematopoiesis worse than young HSCs upon transplantation, preferentially differentiate toward myeloid lineage, and accumulate DNA damage 3, 4, 5, 6, 7. Age‐related changes in HSCs result in reduced immunity in elderly individuals and are linked to cardiovascular diseases, myelodysplasia, and leukemic malignancies 8, 9, 10, 11. While the effect of aging on HSCs is now better characterized, the questions of how these changes arise and whether or not they are reversible remain yet largely unanswered 12, 13, 14.

Until now, the mechanisms found to contribute to the aging of HSCs have been mostly intrinsic to the HSCs 6, 15. These include age‐related accumulation of mutations and cell‐autonomous changes in the transcriptome and epigenome of HSCs 6, 7, 16, 17, 18, 19. Although HSC‐extrinsic factors from the local bone marrow (BM) environment of HSCs—the HSC niche—or systemic factors are critical for HSC maintenance 20, 21, 22, little is known about their contribution to HSC aging.

Recent findings indicate that HSCs occupy a perivascular niche and localize in direct proximity to endothelial cells (ECs) and mesenchymal stromal cells (MSCs) surrounding vessels 23, 24. Among the many cell types in the HSC niche, the ECs and MSCs constitute the main source of stromal cell‐derived factor 1α (SDF‐1α) and stem cell factor (SCF)—extrinsic factors critical for HSC maintenance 25, 26, 27, 28. Specific deletion of either *Sdf1α* or *Scf* in either ECs or MSCs causes hematopoietic collapse or triggers over‐activation of HSCs and their release from the niche 22, 25, 26, 27.

Given the crucial role of the perivascular niche in maintaining HSCs, we hypothesized that HSC‐extrinsic factors that support function of endothelial cells and regulate the activity of hematopoietic mediators may be implicated in HSC aging. This led us to heme oxygenase 1 (HO‐1), a free heme‐degrading enzyme, as a potential niche‐dependent factor that may affect HSC homeostasis.

HO‐1 is an antioxidative, anti‐inflammatory, and antiapoptotic protein, undetectable in most cell types in a steady state but induced under the stress conditions 29. Only in some cell types, as Kupffer cells in the liver or CD4^+^CD25^+^ regulatory T cells, HO‐1 is constitutively expressed 30. HO‐1 deficiency disturbs iron metabolism and redistribution leading to microcytic anemia, what may potentially represent another systemic extrinsic factor that affects HSC exhaustion 31.

We and others showed that beyond its classical role in acute stress responses, HO‐1 is important for SDF‐1α signaling 32 and proper function of endothelial cells 33, 34. Here, we identified cell populations constitutively expressing HO‐1 in the bone marrow niche. Using transplantation and genetic models combined with transcriptional profiling, we demonstrated that HO‐1 regulates the bone marrow niche and protects HSCs from premature exhaustion in cell‐extrinsic manner.

## Results

### Bone marrow endothelial and stromal cells express heme oxygenase‐1 in steady‐state conditions

**Figure 1:**
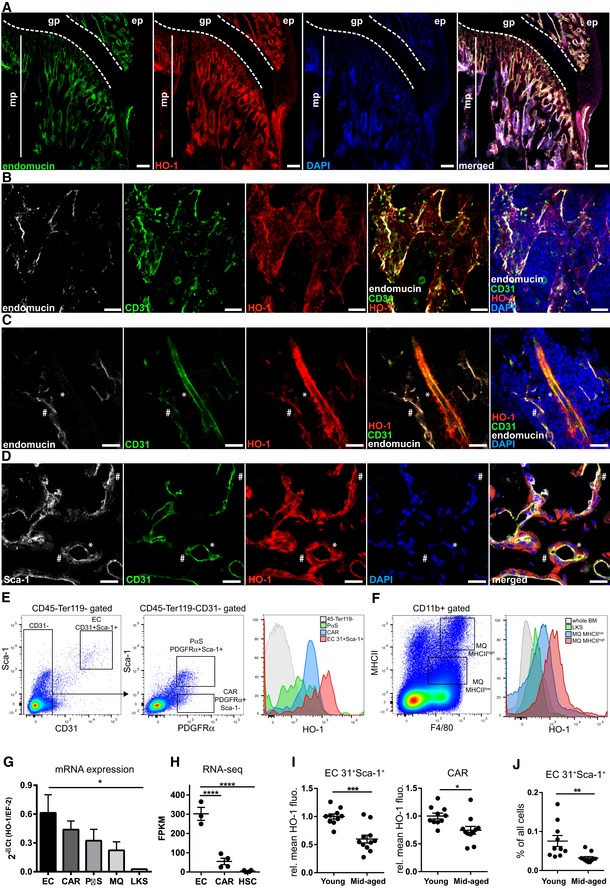
HO‐1 is expressed in BM endothelial cells and pericytes AMetaphysis region in the BM is rich in endomucin^+^ capillaries expressing HO‐1. mp—metaphysis; gp—growth plate; scale bar 100 μm.BThe HO‐1‐positive small capillaries in metaphysis express endomucin and CD31. Shown maximum intensity projection, scale bar 20 μm.CHO‐1 is expressed by smaller endomucin^+^CD31^+^ capillaries (#) as well as in bigger endomucin^−/low^CD31^+^ arteries (*). CD31^−^ pericytes wrapping the artery also express HO‐1 (*); scale bar 20 μm.DHO‐1‐positive capillaries in the metaphysis expressed CD31 and Sca‐1. The capillaries are enveloped by HO‐1‐expressing pericytes. Part of the HO‐1^+^ pericytes express Sca‐1 (#), while others show no or low Sca‐1 signal (*); scale bar 20 μm.EFlow cytometry analysis revealed the highest expression of HO‐1 in CD31^+^Sca‐1^+^ ECs. CAR and PαS populations also express HO‐1, while most of non‐hematopoietic CD45^−^Ter119^−^ are HO‐1‐negative in steady‐state conditions.FBM macrophages (MQs) express HO‐1. The MHCII^high^ MQ expresses higher levels of HO‐1 than MHCII^low^ MQ. Cells within whole HSPC compartment (LKS) express no or low levels of HO‐1 in comparison with MQ.G, HHO‐1 expression on mRNA level quantified by (G) qPCR or (H) RNA‐seq. qPCR analysis based on two independent experiments *n* = 10–11/group, and RNA‐seq analysis has *n* = 3–4/group, two‐tailed *t*‐test for two groups comparison, and one‐way ANOVA with Bonferroni post‐test for multiple group comparison. *P < 0.05, ****P < 0.0001. Data are shown as mean ± SEM.IECs and CARs from middle‐aged animals (11–12 months) express lower levels of HO‐1 protein. Two independent experiments, *n* = 5–10/group. Data are shown as mean ± SEM. *P < 0.05, ***P < 0.001, two‐tailed unpaired *t*‐test.JMiddle‐aged animals have lower frequency of ECs. Two independent experiments, *n* = 10–11/group. Data are shown as mean ± SEM. **P < 0.01, two‐tailed unpaired *t*‐test. The control staining of HO‐1 on HO‐1^−/−^ bone marrow section is provided in Appendix Fig S1.

**Figure EV1:**
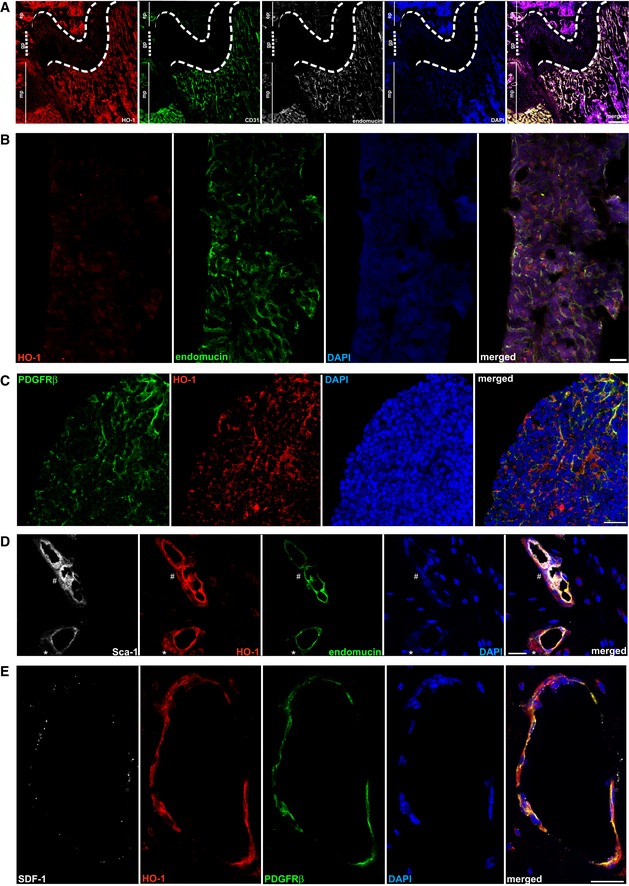
Expression of HO‐1 in BM niche (related to Fig 1) HO‐1 is expressed by CD31^+^endomucin^+^ endothelial cells in metaphysis region of a tibia, scale bar 200 μm.HO‐1 is expressed in sinusoids in diaphysis region, however, at lower levels, scale bar 100 μm.PDGFRβ^+^ stromal cells in diaphysis region of the bone express HO‐1, scale bar 20 μm.Pericytes express HO‐1. Part of the HO‐1^+^ pericytes express Sca‐1 (#), while others express no or low levels of Sca‐1 (*), scale bar 20 μm.HO‐1 is expressed by PDGFRβ^+^ stromal cells. Part of HO‐1^+^PDGFRβ^+^ cells produce SDF‐1α, scale bar 20 μm.

We first determined the distribution of HO‐1 in the murine BM niche under steady‐state conditions. Confocal microscopy analysis of mouse tibias and femurs revealed a high level of HO‐1 protein in endomucin‐positive (endomucin^+^) capillaries in the bone metaphysis (Figs 1A and EV1A), while HO‐1 expression in endomucin^+^ sinusoids in the bone diaphysis, although detectable, was lower (Fig EV1B). Further characterization showed that HO‐1 was expressed in both endomucin^+^CD31^+^ small capillaries (Fig 1B) and bigger endomucin^−/low^CD31^+^ arteries (Fig 1C).

We noticed that pericytes surrounding the capillaries also expressed HO‐1 (Fig 1C and D). Most HO‐1‐expressing pericytes were PDGFRβ‐positive and were present in both the metaphyseal and diaphyseal regions of the bones (Fig EV1C). The HO‐1‐expressing pericytes were also heterogeneous by the Sca‐1 expression, ranging from Sca‐1 positive to Sca‐1 low and negative (Figs 1C and EV1D). Moreover, throughout the BM cavity, we detected PDGFRβ‐positive cells that expressed HO‐1 and produced SDF‐1α (Fig EV1E).

Next, we quantified the expression of HO‐1 in the various bone marrow populations by flow cytometry and real‐time PCR. Consistent with immunochemical staining, we identified a CD45^−^Ter119^−^CD31^+^Sca‐1^+^ population of endothelial cells and two subsets of CD45^−^Ter119^−^CD31^−^PDGFRα^+^ cells that differ in Sca‐1 expression by flow cytometry (Fig 1E). Bone marrow cells with the phenotype CD45^−^Ter119^−^CD31^−^PDGFRα^+^Sca‐1^−^ correspond to the previously described Cxcl12‐abundant reticular cells (CARs) 28, 35, while cells with the CD45^−^Ter119^−^CD31^−^PDGFRα^+^Sca‐1^+^ phenotype correspond to the PDGFRα‐ and Sca‐1‐expressing mesenchymal cells (PαSs) 36. The flow cytometric analysis revealed that ECs, CARs, and PαSs cells all highly express HO‐1 in a steady state, with the highest expression found in the CD31^+^Sca‐1^+^ ECs (Fig 1E). In contrast, the vast majority of CD45^−^Ter119^−^ non‐hematopoietic bone marrow cells did not express HO‐1 (Fig 1E). We also examined HO‐1 in BM hematopoietic cells. Among them, the highest HO‐1 expression was found in macrophages (MQs), particularly in the CD11b^+^F4/80^+^MHCII^high^ fraction, followed by the CD11b^+^F4/80^+^MHCII^low^ fraction (Fig 1F). The pool of hematopoietic stem and progenitor cells (HSPCs), defined as Lin^−^c‐Kit^+^Sca‐1^+^(LKS), expressed only low levels of HO‐1 (Fig 1F).

To directly compare the expression of HO‐1 between the various populations, we sorted CD31^+^Sca‐1^+^ ECs, CARs, PαSs, BM macrophages (CD11b^+^F4/80^+^MHCII^+^), and HSPCs (LKS) and measured the mRNA levels of HO‐1 by qPCR (Fig 1G). Concordantly with results obtained by flow cytometry, HO‐1 mRNA expression was the highest in ECs, high in CARs, PαSs, and MQ, and lowest in HSPCs (Fig 1G). We also checked the expression of HO‐1 in our RNA‐sequencing (RNA‐seq) data sets that include strictly defined sorted LT‐HSCs (LKS CD150^+^CD48^−^CD34^−^) and the two most highly HO‐1‐expressing populations in the BM, namely ECs and CARs. We found that LT‐HSCs expressed negligible levels of HO‐1 compared to ECs and CARs (Fig 1H).

Next, we asked whether the expression of HO‐1 in ECs and CARs changes with age. Using flow cytometry, we observed that HO‐1 protein level was significantly lower in BM CD31^+^Sca‐1^+^ ECs and CARs isolated from mid‐aged animals (11–12 months old) as compared to young animals (1.5–3 months old; Fig 1I). Moreover, the frequency of CD31^+^Sca‐1^+^ ECs in BM was also decreased in mid‐aged animals (Fig 1J). Altogether, we found that HO‐1 is highly expressed under steady‐state conditions in cells that constitute the perivascular BM niche (ECs and CARs) and that its expression in the niche decreases with age.

### Lack of HO‐1 affects numbers of ECs, CARs, and macrophages and dysregulates expression of hematopoietic factors in BM

**Figure 2:**
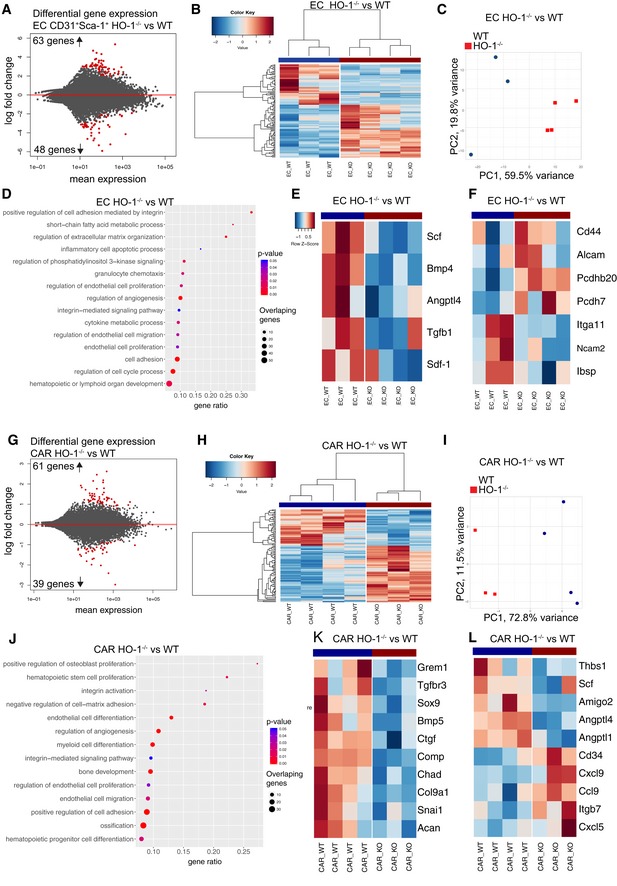
HO‐1 deficiency affects expression of hematopoietic factors in ECs and CARs ARNA‐seq revealed 111 DEGs in HO‐1^−/−^ ECs.B, CThe identified DEGs separated HO‐1^−/−^ and HO‐1^+/+^ ECs by (B) hierarchical clustering and (C) PCA.DSelected GOBP terms enriched in GSEA in HO‐1^−/−^ ECs.E, FHeatmap of selected (E) hematopoietic factors and (F) adhesion molecules expression in HO‐1^−/−^ and HO‐1^+/+^ ECs.GRNA‐seq revealed 100 DEGs in HO‐1^−/−^ CARs.H, IThe identified DEGs separated HO‐1^−/−^ and HO‐1^+/+^ CARs by (H) hierarchical clustering and (I) PCA.JSelected GOBP terms enriched in GSEA in HO‐1^−/−^ CARs.K, LHeatmap of selected (K) skeletal biology and (L) hematopoietic factors expression in HO‐1^−/−^ and HO‐1^+/+^ CARs. Correlation similarity metric and average linkage clustering were used in the presented heatmaps.Data information: Color key on the heatmaps represents gene expression (as *z*‐score among row) red—high expression, blue—low expression.

Given the high HO‐1 expression in ECs and CARs, we investigated how lack of HO‐1 affects these populations. To this aim, we sorted ECs and CARs from wild‐type (HO‐1^+/+^) and HO‐1‐deficient mice (HO‐1^−/−^) and compared their transcriptional profile by RNA‐seq. DESeq2 analysis identified 111 differentially expressed genes (DEGs, FDR < 0.1, Fig 2A, Dataset EV1) that distinguished the HO‐1^−/−^ and HO‐1^+/+^ ECs by hierarchical clustering and principal component analysis (PCA) (Fig 2B and C). Gene set enrichment analysis (GSEA) indicated that lack of HO‐1 not only affects endothelial biology (angiogenesis, endothelial cell migration, and proliferation), but also dysregulates genes related to hematopoiesis (Fig 2D and E). Among the downregulated genes was *Scf* (*Kitl*,* P*_adj_ = 0.09). The other dysregulated genes were implicated in integrin‐mediated adhesion and signaling pathways (Fig 2D and F). Among HO‐1^−/−^ CARs, we found 100 significant DEGs (Fig 2G, Dataset EV2) that also separate HO‐1^−/−^ and HO‐1^+/+^ CARs by hierarchical clustering and PCA (Fig 2H and I). GSEA indicated that these dysregulated genes are related to skeletal biology (osteoblast proliferation, bone development, and ossification) (Fig 2J and K), hematopoietic progenitor differentiation, hematopoietic stem cell proliferation, and myeloid cell differentiation (Fig 2J and L). The remaining significantly enriched gene sets for HO‐1^−/−^ vs. HO‐1^+/+^ CARs also included cell adhesion and integrin signaling pathway, as well as genes involved in the regulation of endothelial migration, proliferation, and angiogenesis (Fig 2J and L).

**Figure 3:**
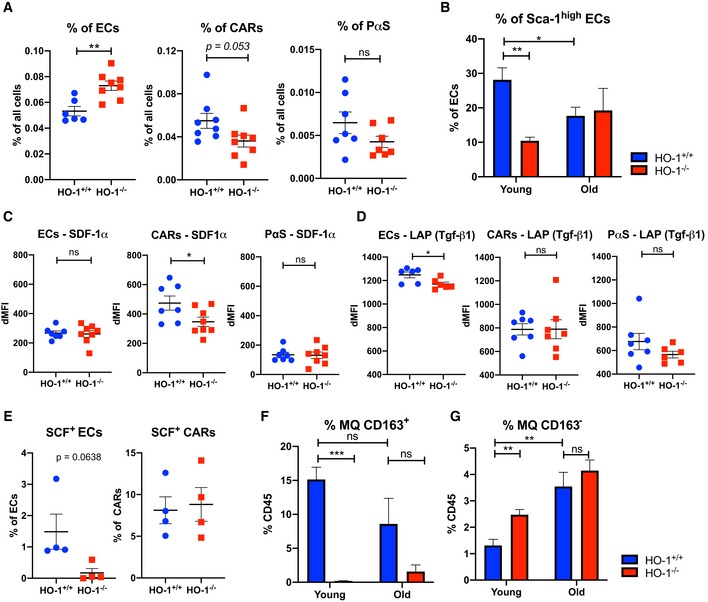
HO‐1^−/−^ mice have disturbed frequency of ECs, CARs, and MQs in BM and altered production of hematopoietic cytokines AHO‐1^−/−^ mice have more ECs and less CARs (*P* = 0.053) in BM, while frequency of PαS does not differ.BYoung HO‐1^−/−^ mice have lower frequency of Sca‐1^high^ fraction among BM ECs, what resembles phenotype of BM ECs in old mice.C, DIntracellular flow cytometry reveals (C) lower levels of SDF‐1α in HO‐1^−/−^ CARs and (D) lower levels of LAP protein in HO‐1^−/−^ ECs.EAnalysis of cell surface SCF revealed lower frequency of SCF^+^ ECs in HO‐1^−/−^ while no differences among CARs.F, GThe frequency of (F) CD163^+^ MQs and (G) CD163^−^ MQs in BM of HO‐1^−/−^ mice is altered.Data information: Where individual data points not shown *n* = 4/group; unpaired two‐tailed *t*‐test. *P < 0.05, **P < 0.01, ***P < 0.001. Data shown are mean ± SEM from a single experiment.

Next, we checked whether altered transcriptome profile of ECs and CARs in HO‐1^−/−^ mice is linked to altered frequency of these populations *in vivo*. We found that frequency of ECs is increased (Fig 3A) while frequency of CARs is decreased (*P* = 0.053) in BM of HO‐1^−/−^ animals. We did not observe significant differences in frequency of PαS population. To further explore the heterogeneity of ECs, we analyzed the frequency of Sca‐1^high^ fraction of ECs. The Sca‐1^high^ ECs fraction consists of arterial and type H ECs that are diminished with aging 37, 38. Indeed, we observed that frequency of Sca‐1^high^ ECs is decreased in old HO‐1^+/+^ animals (18–24 months old). However, HO‐1^−/−^ mice have decreased frequency of Sca‐1^high^ ECs already in young age (3 months), what resembles phenotype observed in old HO‐1^+/+^ animals (Fig 3B). Then, we checked the production of hematopoietic factors by intracellular flow cytometry. We found that HO‐1^−/−^ CARs produce less SDF‐1α, while there were no differences among ECs and PαS (Fig 3C). In contrast, the production of LAP (Tgf‐β1) was lower in HO‐1^−/−^ ECs, but did not differ in the other tested populations (Fig 3D). While we were not able to reliably detect intracellular SCF, we analyzed cells positive for membrane‐bound, cell surface SCF. We noticed a small fraction of SCF^+^ cells among the ECs and tendency (*P* = 0.064) to lower frequency of SCF^+^ among ECs in HO‐1^−/−^ animals (Fig 3E). The frequency of SCF^+^ among CARs did not differ (Fig 3E), and we did not detect SCF^+^ PαS.

Finally, as HO‐1 was highly expressed in MQ (defined as CD11b^+^MHCII^+^F4/80^+^), we checked the frequency of MQs in HO‐1^−/−^ animals. First, we observed that CD163^+^ MQ fraction, which is involved in formation of erythroblastic islands and removal of senescent erythrocytes 39, is drastically reduced in old and young HO‐1^−/−^ animals (Fig 3F). In contrast, CD163^−^ MQs are more frequent in young, but not in old HO‐1^−/−^ animals (Fig 3G).

### Young HO‐1^−/−^ mice possess expanded pool of activated HSC

**Figure 4:**
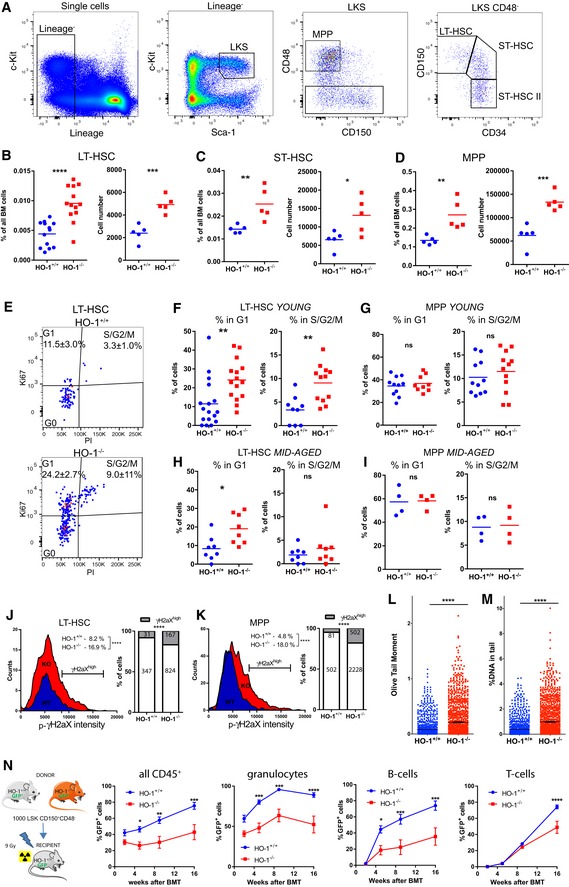
HO‐1 deficiency causes loss of LT‐HSC quiescence and expansion of stem cell pool AGating strategy of hematopoietic stem and progenitor cells.B–DYoung HO‐1^−/−^ mice possess higher number of (B) LT‐HSCs, (C) ST‐HSCs, and (D) MPPs.EExemplary analysis of cell cycle with Ki67 and nuclear dye.FMore young HO‐1^−/−^ LT‐HSCs are in G1 and S/G2/M cell cycle phases. The presented cell cycle analysis is from two independent experiments.GYoung HO‐1^−/−^ MPPs do not differ in cell cycling from young HO‐1^+/+^ MPPs. The presented cell cycle analysis is from two independent experiments.HMore old HO‐1^−/−^ LT‐HSCs are in G1 phase in comparison with old HO‐1^+/+^ LT‐HSCs, but not in S/G2/M phase. The presented cell cycle analysis is from two independent experiments.IOld MPPs do not differ in cell cycling between genotypes. The presented cell cycle analysis is from two independent experiments.JYoung HO‐1^−/−^ LT‐HSCs contain more γH2aX^high^ cells than young HO‐1^+/+^; 378–991 cells analyzed from seven mice/group.KYoung HO‐1^−/−^ MPPs contain more γH2aX^high^ cells than young HO‐1^+/+^; 1,693–2,790 cells analyzed from seven mice/group.L, MAlkaline comet assay revealed that HO‐1^−/−^ LT‐HSCs possess (L) higher oil tail moment and (M) more DNA in the comet tail. 1,068–1,189 cells analyzed from six mice/group.NHSCs from young HO‐1^−/−^ mice provide worse hematopoietic reconstitution after transplantation than HO‐1^+/+^ HSCs. Data are shown as mean ± SEM, *n* = 8–9 mice/group.Data information: For comparison of two groups unpaired, two‐tailed *t*‐test was used. For contingency analysis (J and K), Fisher's exact test was applied. For two variable comparisons (N), 2‐way ANOVA with Bonferroni post‐test was used. *P < 0.05, **P < 0.01, ***P < 0.001, ****P < 0.0001.

**Figure EV2:**
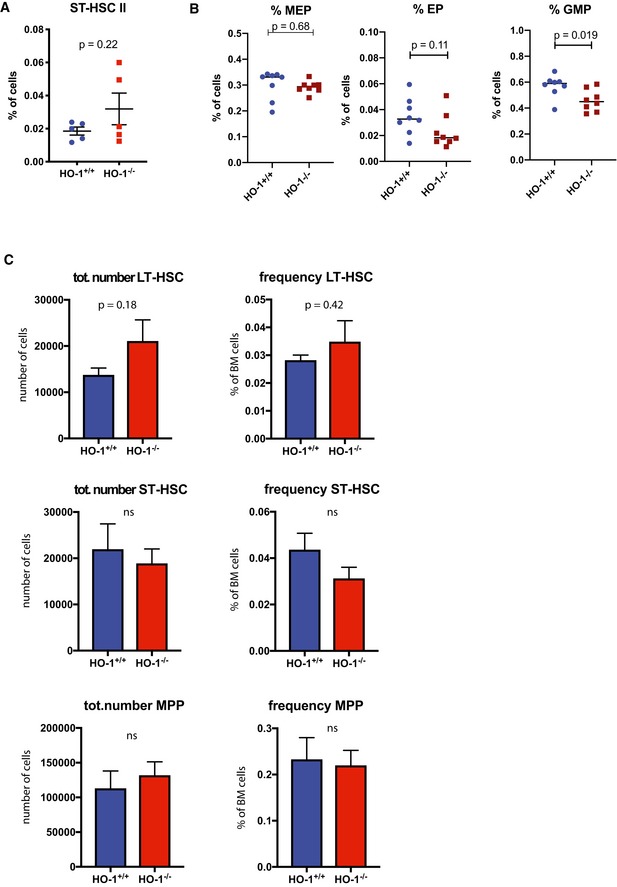
Frequency of hematopoietic stem and progenitors cells in HO‐1^+/+^ and HO‐1^−/−^ mice (related to Fig 4) Frequency of ST‐HSC II population in young HO‐1^+/+^ and HO‐1^−/−^ mice. Two‐tailed unpaired *t*‐test.Frequency of MEP, EP, and GMP populations in young HO‐1^+/+^ and HO‐1^−/−^ mice. Two‐tailed unpaired *t*‐test.Frequency and total number of LT‐HSCs, ST‐HSCs, and MPPs in 12‐month‐old mice, *n* = 5 mice/group. Unpaired, two‐tailed *t*‐test.

HO‐1 deficiency in ECs and CARs alters the expression of niche‐derived factors necessary for HSC function (Figs 2 and 3). Therefore, we investigated how HO‐1 deficiency affects LT‐HSCs and downstream progenitors (Fig 4A). Young (1.5–3 months old) HO‐1^−/−^ mice have increased frequency and total number of LT‐HSCs (LKS CD150^+^CD48^−^CD34^−^, Fig 4B), ST‐HSCs (LKS CD150^mid^CD48^−^CD34^+^, Fig 4C), and MPPs (LKS CD150^−^CD48^+^, Fig 4D) compared to young HO‐1^+/+^ mice. We did not observe significant difference among ST‐HSC II population (Fig EV2A). Among committed progenitors, frequency of early megakaryocyte–erythroid progenitors (Lin^−^c‐Kit^+^Sca‐1^−^CD48^mid^CD150^+^) and erythroid progenitors (Lin^−^c‐Kit^+^Sca‐1^−^CD48^−^CD150^−^) was unchanged in HO‐1^−/−^ mice (Fig EV2B), while frequency of granulocyte–monocyte progenitors was decreased (Lin^−^c‐Kit^+^Sca‐1^−^CD48^+^CD150^−^CD34^+^, Fig EV2B). However, in mid‐aged mice (12 months old), the frequency and total number of LT‐HSCs, ST‐HSCs, and MPPs did not differ between HO‐1^−/−^ and HO‐1^+/+^ genotypes (Fig EV2C). Next, we determined the cell cycle status of the LT‐HSCs isolated from HO‐1^−/−^ mice. The flow cytometry analysis based on Ki67 and DNA content (Fig 4E) revealed that there were significantly more LT‐HSCs in G1 and S/G2/M phases in young HO‐1^−/−^ mice than in young HO‐1^+/+^ mice (Fig 4F). Notably, this effect was specific to LT‐HSCs, as young HO‐1^−/−^ MPPs showed the same cell cycle status as young HO‐1^+/+^ MPPs (Fig 4G). Among older mice, we still observed a higher percentage of LT‐HSCs in G1 phase, although there was no difference in the frequency of LT‐HSCs actively cycling in S/G2/M phases (Fig 4H). We also did not observe any differences in cell cycle status among MPPs in older HO‐1^−/−^ animals (Fig 4I).

Next, we measured the levels of phosphorylated H2aX (p‐γH2aX), an established marker of aged LT‐HSCs, linked to increased DNA damage and replication stress 7, 40, 41. Levels of γH2aX were significantly higher in LT‐HSCs (Fig 4J) and MPPs (Fig 4K) in young HO‐1^−/−^ in comparison with young wild‐type mice. We also noticed that in young HO‐1^+/+^ mice, a lower percentage of MPPs as compared to LT‐HSCs is p‐γH2aX^high^ (Fig 4J and K), which is consistent with recent reports indicating that DNA repair is activated upon cell cycle entry and differentiation into MPPs 42. To analyze directly the DNA damage, we performed the alkaline comet assay on LT‐HSCs from young HO‐1^−/−^ and HO‐1^+/+^ mice. Consistently with p‐γH2aX analysis, HO‐1^−/−^ LT‐HSCs had significantly higher oil tail moment (Fig 4L) and more DNA in tail (Fig 4M), indicating more DNA damage.

Finally, we assessed the functionality of HO‐1^−/−^ ‐derived LT‐HSCs in a transplantation model (Fig 4N). Transplanted HO‐1^−/−^ LT‐HSCs were less efficient at reconstituting irradiated HO‐1^+/+^ recipients than control HO‐1^+/+^ LT‐HSCs in all tested lineages (granulocytes, B cells, and T cells) (Fig 4N). In conclusion, the LT‐HSCs from young HO‐1^−/−^ mice exhibit features that resemble the phenotype of aged LT‐HSCs, including an expanded pool, more DNA damage, and impaired regenerative potential.

### Transcriptome profile of young HO‐1^−/−^ LT‐HSC resembles transcriptome profile of aged LT‐HSCs

Our analysis showed that LT‐HSCs isolated from young HO‐1^−/−^ mice display markers of aged LT‐HSCs. Therefore, we hypothesized that HO‐1 deficiency may contribute to the premature exhaustion of LT‐HSCs. To test this hypothesis in the most comprehensive way possible, we profiled the transcriptome of LT‐HSCs from young (3‐months old) and aged (~ 18 months old) mice of HO‐1^+/+^ and HO‐1^−/−^ genotype by RNA sequencing.

**Figure 5:**
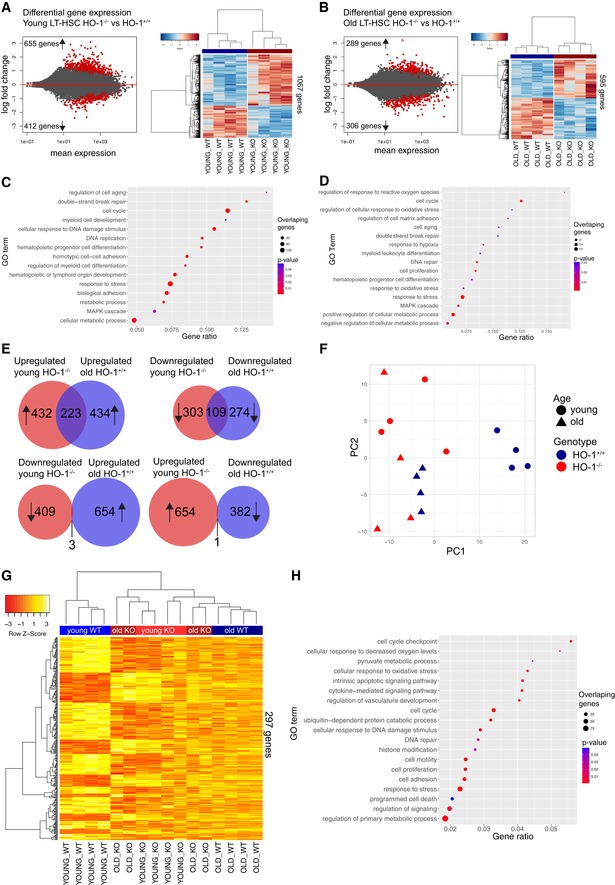
LT‐HSCs from young HO‐1^−/−^ mice possess transcriptional profile resembling aged LT‐HSCs RNA‐seq revealed 1,067 DEGs between young HO‐1^−/−^ and HO‐1^+/+^ LT‐HSCs.RNA‐seq revealed 595 DEGs between old HO‐1^−/−^ and HO‐1^+/+^ LT‐HSCs.Selected GOBP terms enriched in GSEA in young HO‐1^−/−^ LT‐HSCs.Selected GOBP terms enriched in GSEA in old HO‐1^−/−^ LT‐HSCs.Significant part of DEGs (31% total) in young HO‐1^−/−^ LT‐HSCs overlaps with DEGs identified in LT‐HSCs during physiological aging.PC1 in PCA on selected 1,148 genes separated young HO‐1^+/+^ LT‐HSCs from other groups, clustering the young HO‐1^−/−^ LT‐HSCs together with old LT‐HSCs.Hierarchical clustering on the 20% of most correlated 297 genes with PC1 showed two major clusters that include young HO‐1^+/+^ LT‐HSCs in one and old LT‐HSCs with young HO‐1^−/−^ LT‐HSCs in second.Selected GOBP terms enriched in GSEA based on 297 identified genes.Data information: Color key on the heatmaps represents gene expression (as *z*‐score among row).

First, we compared LT‐HSCs from young HO‐1^−/−^ and HO‐1^+/+^ mice to determine the effect of HO‐1 deficiency on the LT‐HSC transcriptome. We identified 1,067 significant DEGs (FDR < 0.1) between LT‐HSCs from young HO‐1^−/−^ and young HO‐1^+/+^ mice, which segregated these groups into distinct clusters (Fig 5A, Dataset EV3). Similarly, we identified 595 significant DEGs (FDR < 0.1) between LT‐HSCs from old HO‐1^−/−^ and old HO‐1^+/+^ mice, which segregated these groups into distinct clusters (Fig 5B, Dataset EV4). We performed GSEA using the Gene Ontology Biological Process (GOBP) database to ask which biological processes are associated with these DEGs. The comparison of HO‐1^−/−^ and HO‐1^+/+^ LT‐HSCs from both young and old mice identified similar GOBP terms (Fig 5C and D). We found that the significant pathways were associated with cell aging, cell cycle, and DNA damage repair, consistent with our flow cytometry results of cell cycle status and DNA damage (Fig 3, Appendix Fig S2). The DEGs were additionally implicated in cell adhesion, metabolic control, and regulation of hematopoietic differentiation (Fig 5C and D). Interestingly, the GSEA also identified processes related to response to reactive oxygen species (ROS) and response to hypoxia, but only between old HO‐1^−/−^ and HO‐1^+/+^ LT‐HSCs (Fig 5D).

Next, we asked whether the genes dysregulated in young HO‐1^−/−^ LT‐HSCs resemble the expression changes in LT‐HSCs during aging. First, we identified 1,040 significant gene expression changes (FDR < 0.1) in LT‐HSCs acquired with age by comparing young HO‐1^+/+^ LT‐HSC and old HO‐1^+/+^ LT‐HSC (Dataset EV5). Then, we compared the DEGs between aged HO‐1^+/+^ and young HO‐1^+/+^ LT‐HSCs with the DEGs between young HO‐1^−/−^ and young HO‐1^+/+^ LT‐HSCs. We found 223 shared DEGs that are upregulated in both young HO‐1^−/−^ LT‐HSCs and old HO‐1^+/+^ LT‐HSCs compared to young HO‐1^+/+^ LT‐HSCs (Fig 5E, Dataset EV6), and 109 shared DEGs that are downregulated in young HO‐1^−/−^ LT‐HSCs and old HO‐1^+/+^ LT‐HSCs compared to young HO‐1^+/+^ LT‐HSCs (Fig 5E, Dataset EV6). In contrast, we found only four DEGs that are significantly changed in opposite directions in young HO‐1^−/−^ LT‐HSCs and old HO‐1^+/+^ LT‐HSCs (Fig 5E). This suggests that 31% of DEGs between young HO‐1^−/−^ LT‐HSCs and young HO‐1^+/+^ LT‐HSCs are shared with the DEGs found between aged HO‐1^+/+^ LT‐HSC and young HO‐1^+/+^ LT‐HSC, which represents normal aging.

To identify the core genes that make transcriptional profile of HO‐1^−/−^ similar to aged LT‐HSCs, we asked whether there are genes similarly expressed in young HO‐1^−/−^ LT‐HSCs and old LT‐HSCs regardless of the genotype, but differentially expressed in young HO‐1^+/+^ LT‐HSCs. We found 1,448 DEGs that satisfied these criteria and performed principal component analysis (PCA) based on the identified DEGs. The PC1 component separated the young HO‐1^+/+^ LT‐HSCs from the other samples and grouped young HO‐1^−/−^ LT‐HSC with old HO‐1^−/−^ and HO‐1^+/+^ LT‐HSC (Fig 5F). To identify the most significant genes that provide this separation, we calculated the top 20% of most correlated (both positively and negatively) genes with PC1. We identified 297 genes that clustered young HO‐1^+/+^ LT‐HSCs separately and young HO‐1^−/−^ LT‐HSCs together with old HO‐1^−/−^ and old HO‐1^+/+^ LT‐HSCs (Fig 5G). GSEA revealed that these 297 genes are involved in a broad variety of biological processes, including cell cycle, DNA repair, metabolism, cell adhesion, response to stress, signaling, histone modification, protein catabolic processes, and apoptosis (Fig 5H).

**Figure EV3:**
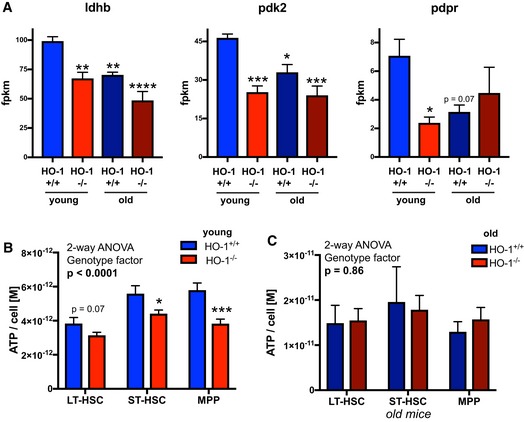
Decreased expression of genes regulating pyruvate metabolism in young HO‐1^−/−^ LT‐HSCs and old LT‐HSCs is associated with lower ATP levels ALdhb, Pdk2, and Pdpr are downregulated in young HO‐1^−/−^ LT‐HSCs, but not in old HO‐1^−/−^ LT‐HSCs. Analyzed by RNA‐seq. Data are shown as mean ± SEM, four mice/group, *P < 0.05, **P < 0.01, ***P < 0.001, ****P < 0.0001 comparing to young HO‐1^+/+^ group, two‐tailed unpaired *t*‐test.B, CATP levels are (B) lower in young HO‐1^−/−^ LT‐HSCs comparing to young HO‐1^+/+^ LT‐HSCs, (C) but not in old HO‐1^−/−^ LT‐HSCs comparing to old HO‐1^+/+^ LT‐HSCs. ATP levels measured in two independent experiments. Data are shown as mean ± SEM, *n* = 8–18/group. *P < 0.05, ***P < 0.001, two‐tailed unpaired *t*‐test.

Particularly, this GSEA above indicated that alterations in expression of genes regulating pyruvate metabolism converge the young HO‐1^−/−^ LT‐HSCs with old LT‐HSCs. Pyruvate metabolism and the regulation of glycolysis are quiescence checkpoints in HSCs 43. Decreased expression of genes regulating pyruvate and glycolysis in LT‐HSCs, like pyruvate dehydrogenase kinases (*Pdks*) and lactate dehydrogenases (*Ldhs*), respectively, is linked with lower ATP levels 43. We found that young HO‐1^−/−^ LT‐HSCs downregulate expression of *Ldhb*,* Pdk2*, and *Pdpr*, similarly to old LT‐HSCs (Fig EV3A). Consistently, we observed that HO‐1 deficiency results in lower amounts of ATP in stem and progenitor cells in young mice (Fig EV3B) but not in old mice (Fig EV3C).

Overall, transcriptomic analysis of young HO‐1^−/−^ LT‐HSCs revealed similarity to transcriptome profile of old HO‐1^+/+^ LT‐HSC. We found that transcriptome similarity of young HO‐1^−/−^ LT‐HSC to aged LT‐HSC is manifested by expression of genes involved in variety of biological process, including downregulation of genes involved in the pyruvate metabolism, that was linked with lower amount of ATP.

### Extrinsic HO‐1 deficiency causes exhaustion of LT‐HSCs

**Figure 6:**
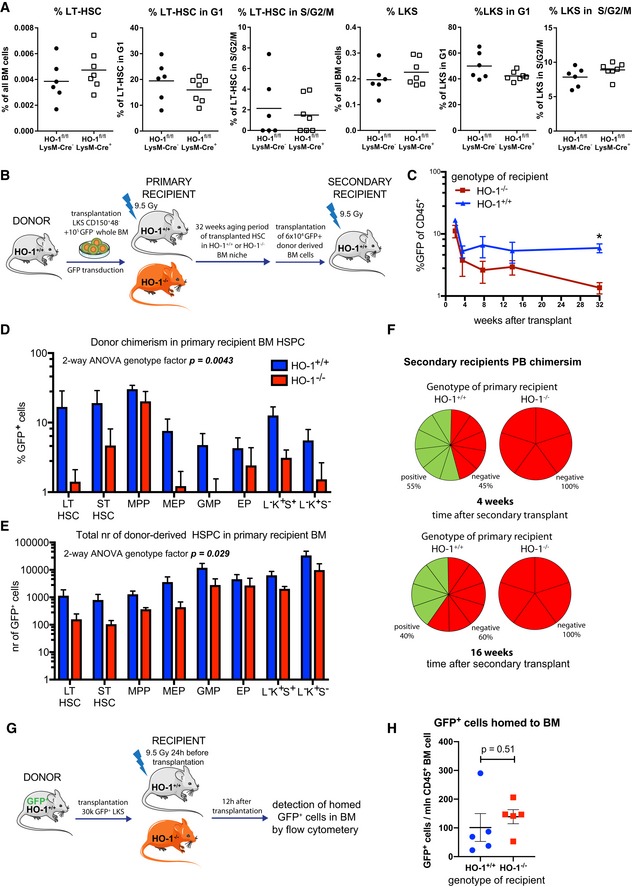
HO‐1 deficiency in the niche causes exhaustion of LT‐HSCs ADeletion of HO‐1 in myeloid lineage did not cause expansion of LT‐HSCs and did not alter their cell cycle status.BScheme of the experiment assessing long‐term effect of HO‐1‐deficient niche on function of HSCs.CLong‐term PB chimerism derived from HSCs transplanted to HO‐1^−/−^ recipients is lower than from HSCs transplanted to HO‐1^+/+^ recipients. *P < 0.05, 2‐way ANOVA. Data are shown as mean ± SEM, *n* = 5–12/group.D, E(D) Chimerism among BM HSPC fractions and (E) total number of cells derived from HSCs transplanted to HO‐1^−/−^ recipients are lower than from HSCs transplanted to HO‐1^+/+^ recipients. Data are shown as mean ± SEM, *n* = 5–12/group.FHSCs that were initially transplanted to primary HO‐1^−/−^ recipients did not reconstitute secondary HO‐1^+/+^ recipients, in contrast to HSCs that were initially transplanted to HO‐1^+/+^ recipients, *n* = 5–12/group.GScheme of the experiment assessing short‐term homing of HSPC to HO‐1‐deficient niche.HShort‐term homing of HSPC to HO‐1^+/+^ or HO‐1^−/−^ BM did not differ. Data are shown as mean ± SEM, *n* = 5/group, unpaired *t*‐test.

We found that LT‐HSCs from HO‐1^−/−^ mice are impaired and exhibit the transcriptional profile similar to aged LT‐HSCs. As we demonstrated, expression of HO‐1 is low in LT‐HSCs, but high in macrophages, ECs, and CARs (Fig 1E–H). Therefore, we hypothesized that the observed LT‐HSC phenotype in HO‐1^−/−^ mice is caused by HO‐1 deficiency in the HSC microenvironment. To verify this, we first asked whether HO‐1 expression in macrophages is necessary to maintain quiescence of LT‐HSCs. Macrophages, especially their CD163^+^ fraction, are responsible for proper iron redistribution, and their deficiency is a primary cause of microcytic anemia in HO‐1^−/−^ mice 44. Therefore, we analyzed the LT‐HSCs in mice with conditional deletion of HO‐1 with Cre recombinase driven by the lysozyme M promoter (HO‐1^fl/fl^; LysM‐Cre) 45. Deletion of HO‐1 by LysM‐Cre in monocyte–macrophage and neutrophil lineages 45, 46 did not reproduce the impaired LT‐HSC phenotype observed in global HO‐1‐deficient mice. The HO‐1^fl/fl^;LysM‐Cre mice did not show expansion of LT‐HSCs or HSPCs, and there were no differences in the cell cycle, suggesting that lack of HO‐1 in monocyte–macrophage lineage and neutrophils alone does not affect LT‐HSCs (Fig 6A).

Next, to investigate the general cell‐extrinsic role of HO‐1 in regulation of LT‐HSCs, we transplanted HO‐1^+/+^ HSCs to lethally irradiated HO‐1^−/−^ or HO‐1^+/+^ recipients and followed donor chimerism for 32 weeks (Fig 6B). We did not observe any increased mortality or morbidity of HO‐1^−/−^ mice upon irradiation. During the first 3 weeks after transplant, we did not find any significant differences in donor chimerism between HO‐1^−/−^ and HO‐1^+/+^ recipients as measured by percent of GFP^+^CD45^+^ peripheral blood (PB) cells (Fig 6C). However, starting from 7 weeks after transplant, chimerism in HO‐1^−/−^ recipients decreased, reaching the highest and most significant difference after 32 weeks (Fig 6C).

Looking within the bone marrow after 32 weeks, we found lower percent of chimerism (Fig 6D) and a total number of donor‐derived cells (Fig 6E) in BM HSC and HSPC fractions in HO‐1^−/−^ than in HO‐1^+/+^ recipients. To evaluate the function of donor‐derived HO‐1^+/+^ HSCs that were aged for 32 weeks in HO‐1^−/−^ or HO‐1^+/+^ primary recipients, we serially transplanted the same number (6 × 10^4^) of donor‐derived GFP^+^ whole BM cells to secondary young HO‐1^+/+^ recipients (Fig 6B). In this experimental setting, we transplanted BM HO‐1^+/+^ cells to the HO‐1^+/+^ secondary recipients, and the only thing that differentiated the groups was the 32‐week aging period in HO‐1^+/+^ or HO‐1^−/−^ primary recipients (Fig 6B). We found that only BM cells from HO‐1^+/+^ recipients reconstituted secondary recipients, while BM cells from HO‐1^−/−^ primary recipients failed to reconstitute secondary recipients (Fig 6F).

The observed long‐term effect may potentially result from impaired initial homing of transplanted cells to HO‐1^−/−^ niche. To verify this possibility, we transplanted 30k GFP^+^ HSPC (defined as LSK) into irradiated HO‐1^+/+^ or HO‐1^−/−^ recipients and checked the short‐term (12 h) homing to BM (Fig 6G). We did not observe statistical difference in short‐term homing to BM of HO‐1^+/+^ or HO‐1^−/−^ recipients (Fig 6H), what suggests that diminished long‐term reconstitution in HO‐1^−/−^ mice does not result from impaired short‐term homing.

Altogether, these results demonstrate that HSC‐extrinsic HO‐1 deficiency causes exhaustion of LT‐HSCs and impairs their regenerative potential.

### Transplantation to HO‐1^+/+^ recipients can restore the function and transcriptional profile of HO‐1^−/−^ LT‐HSCs

**Figure 7:**
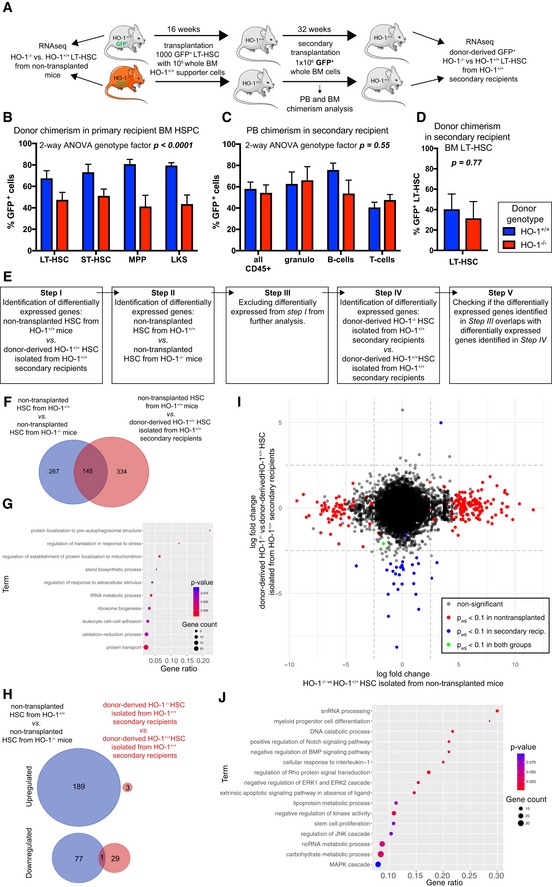
HO‐1^+/+^ expression in the recipients restores function and transcriptional profile of HO‐1^−/−^ LT‐HSCs AScheme of the experiment verifying if the HO‐1^+/+^ niche is able to reverse phenotype of HO‐1^−/−^ LT‐HSCs.BTransplantation of HO‐1^−/−^ HSCs provides lower chimerism among HSPC fractions in primary recipients. Data are shown as mean ± SEM, *n* = 7–8 mice/group.C, DTransplantation of the same number of donor‐derived BM cells from primary recipients provides the same (C) PB chimerism and (D) BM LT‐HSC chimerism. Data are shown as mean ± SEM, *n* = 7–8 mice/group.EAnalysis pipeline used to determine whether transplantation of HO‐1^−/−^ HSCs to the HO‐1^+/+^ recipients reverses their transcriptional alterations.FOverlap between DEGs in young HO‐1^−/−^ HSCs and DEGs changed by transplantation alone. A total of 145 overlapping genes were excluded from further analysis.GGSEA based on 145 excluded genes indicates processes that cannot be analyzed with the pipeline.HOnly 1 out of 267 DEGs identified in non‐transplanted HO‐1^−/−^ LT‐HSCs was still dysregulated in HO‐1^−/−^ LT‐HSCs transplanted twice to the wild‐type HO‐1^+/+^.IComparison of gene log‐fold changes in non‐transplanted HO‐1^−/−^ LT‐HSCs and HO‐1^−/−^ LT‐HSCs transplanted twice to the wild‐type HO‐1^+/+^ showed that transplantation of HO‐1^−/−^ LT‐HSCs twice to the wild‐type niche reverses their transcriptional alterations.JGSEA based on genes that were altered in non‐transplanted HO‐1^−/−^ LT‐HSCs, but were normalized by double transplantation to the wild‐type niche.

Given that extrinsic HO‐1 is necessary for proper HSC function, we asked whether the HO‐1^+/+^ niche can restore the function of LT‐HSCs isolated from young HO‐1^−/−^ mice (Fig 7A). As shown in Fig 4N, young HO‐1^−/−^ HSCs transplanted into primary HO‐1^+/+^ recipients are less efficient at reconstituting hematopoiesis than young HO‐1^+/+^ HSCs, even after 16 weeks of transplant. This indicates that primary transplantation to the HO‐1^+/+^ niche is not sufficient to restore the function of young HO‐1^−/−^ LT‐HSCs.

However, primary recipients transplanted with HO‐1^−/−^ LT‐HSCs have a lower donor‐derived chimerism among HSPCs (Fig 7B), which may explain the lower PB chimerism. To test whether secondary transplantation into the HO‐1^+/+^ recipients can restore the function of HO‐1^−/−^ LT‐HSCs, 16 weeks after primary transplant we transplanted equal numbers of GFP^+^ donor‐derived BM cells from HO‐1^+/+^ primary recipients containing GFP^+^HO‐1^+/+^ chimeric BM or GFP^+^HO‐1^−/−^ chimeric BM into HO‐1^+/+^ secondary recipients (Fig 7A). The frequency of LT‐HSCs in donor‐derived BM cells was the same in both groups (Appendix Fig S3). After secondary transplantation of LT‐HSCs initially derived from HO‐1^−/−^ and HO‐1^+/+^ donors, recipient mice exhibited no difference in PB chimerism in any of tested lineages (Fig 7C) and no difference in chimerism among BM LT‐HSCs 36 weeks later (Fig 7D). This indicates that the HO‐1^+/+^ niche can restore function of HO‐1^−/−^ LT‐HSCs after secondary transplantation to HO‐1^+/+^ recipients.

Additionally, we compared the transcriptome of freshly isolated LT‐HSCs from HO‐1^+/+^ and HO‐1^−/−^ mice, as well as from donor‐derived HO‐1^+/+^ and HO‐1^−/−^ LT‐HSCs isolated from secondary recipients 36 weeks after transplantation (Fig 7A). By sorting similar numbers of identically defined LT‐HSCs and processing all samples with the same RNA‐seq library preparation and sequencing steps, we verified whether the secondary HO‐1^+/+^ niche reverses the defective transcriptome of LT‐HSCs freshly isolated from HO‐1^−/−^ mice. Given that transplantation alone changes the transcriptome of LT‐HSCs, we identified and then excluded the genes whose expression significantly changes with transplantation (Fig 7E). To identify genes that are affected by transplantation alone, we compared freshly isolated, non‐transplanted HO‐1^+/+^ LT‐HSCs with serially transplanted HO‐1^+/+^ LT‐HSC isolated from the secondary recipient. We identified 479 DEGs (Fig 7E step I) and subsequently excluded them from our analysis. Next, we identified 412 DEGs between freshly isolated, non‐transplanted LT‐HSCs from HO‐1^+/+^ and HO‐1^−/−^ mice, of which 267 DEGs did not overlap with previously excluded genes that were changed by transplantation alone (Fig 7E step II, III, 7F) and were included in the next steps of analysis. A total of 145 overlapping DEGs that were excluded from the analysis at this step (Fig 7F, genes that were changed between non‐transplanted HO‐1^+/+^ and HO‐1^−/−^ LT‐HSCs and changed by transplantation alone) were used for GSEA to indicate processes that cannot be addressed with this experimental scheme (Fig 7G).

Finally, we checked whether secondary transplantation to wild‐type HO‐1^+/+^ recipients reverses transcriptional alterations of HO‐1^−/−^ LT‐HSCs. When we compared HO‐1^+/+^ and HO‐1^−/−^ LT‐HSCs from secondary recipients (Fig 7E step IV), we identified only 33 DEGs (Fig 7H). Next, we verified how many of 267 DEGs identified in non‐transplanted HO‐1^−/−^ LT‐HSCs are still dysregulated in HO‐1^−/−^ LT‐HSCs transplanted twice to the wild‐type HO‐1^+/+^ secondary recipients (Fig 7E step V), and we found only one overlapping gene (Fig 7H).

We also compared fold changes of DEGs identified between non‐transplanted HO‐1^−/−^ LT‐HSCs and HO‐1^+/+^ LT‐HSCs to fold changes of DEGs identified between serially transplanted HO‐1^−/−^ LT‐HSCs and HO‐1^+/+^ LT‐HSCs from the secondary recipients (Fig 7I). The observed pattern indicates that most genes dysregulated in non‐transplanted HO‐1^−/−^ LT‐HSCs were normalized in HO‐1^−/−^ LT‐HSCs that were transplanted twice into HO‐1^+/+^ recipients (Fig 7I). The GSEA based on genes that were normalized upon transplantations (2.5 log‐fold changed in young in non‐transplanted HO‐1^−/−^ LT‐HSC, but less than 2.5 log‐fold changed in transplanted HO‐1^−/−^ LT‐HSCs from the secondary recipients, Fig 7I) implicated processes related to snRNA processing and ncRNA metabolism, myeloid differentiation, Notch signaling pathway, BMP signaling pathway, Rho signaling, several metabolic processes, as well as processes and pathways involved in proliferation (Fig 7J). In summary, serial transplantation of HO‐1^−/−^ LT‐HSCs into HO‐1^+/+^ recipients restores function and reverses transcriptional alterations of HO‐1^−/−^ LT‐HSCs.

### HSCs co‐cultured with HO‐1^−/−^ mesenchymal stromal cells give rise to colonies with altered growth kinetic and differentiation

**Figure 8:**
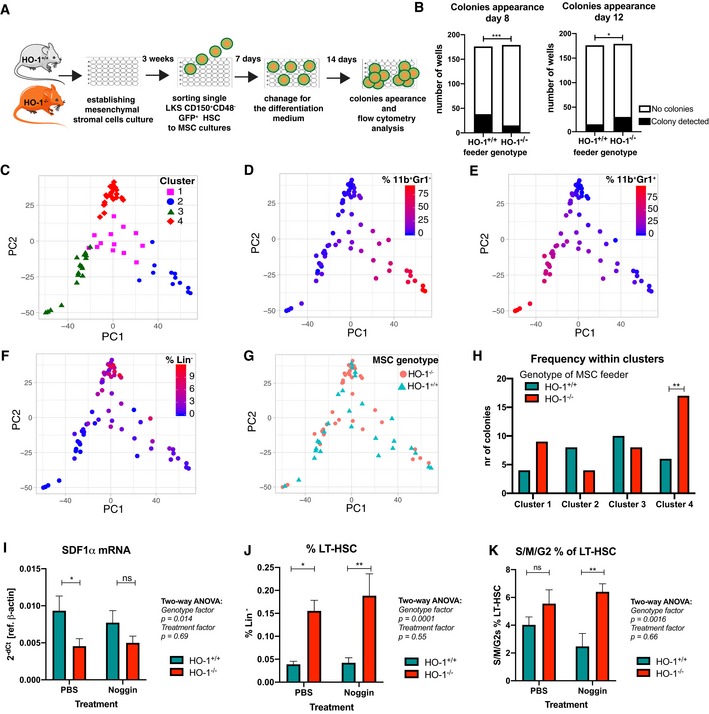
HO‐1 deficiency in MSCs affects growth and differentiation of HSC‐derived hematopoietic colonies AScheme of experiment based on HSC co‐culture with MSCs and colony formation from single cells in differentiation media.BFrequency of colonies appearing at day 8 and day 12 from HSCs co‐cultured with HO‐1^+/+^ or HO‐1^−/−^ MSCs. *P < 0.05, ***P < 0.001, Fisher's exact test.C–FPCA and cluster analysis of the colonies based on initial flow cytometry gating revealed (C) four distinct clusters of colonies that differ by frequency of (D) CD11b^+^Gr1^−^, (E) CD11b^+^Gr1^+^, and (F) cells lacking expression of analyzed lineage markers (Lin^−^).G, HAnalysis of frequency of colonies derived from HO‐1^+/+^ or from HO‐1^−/−^ MSC co‐cultures among identified clusters. Enrichment in cluster analyzed by Fisher's exact test, **P < 0.01, two‐tailed unpaired *t*‐test.ISdf1α mRNA levels in BM of HO‐1^+/+^ or HO‐1^−/−^ mice after administration of Noggin. Data are shown as mean ± SEM. *P < 0.05, two‐tailed unpaired *t*‐test, *n* = 10/group.J, K(J) LT‐HSC frequency and (K) cell cycling status of LT‐HSCs after administration of Noggin to HO‐1^+/+^ or HO‐1^−/−^ mice. Data are shown as mean ± SEM. *P < 0.05, **P < 0.01, two‐tailed unpaired *t*‐test, *n* = 8–10/group.

**Figure EV4:**
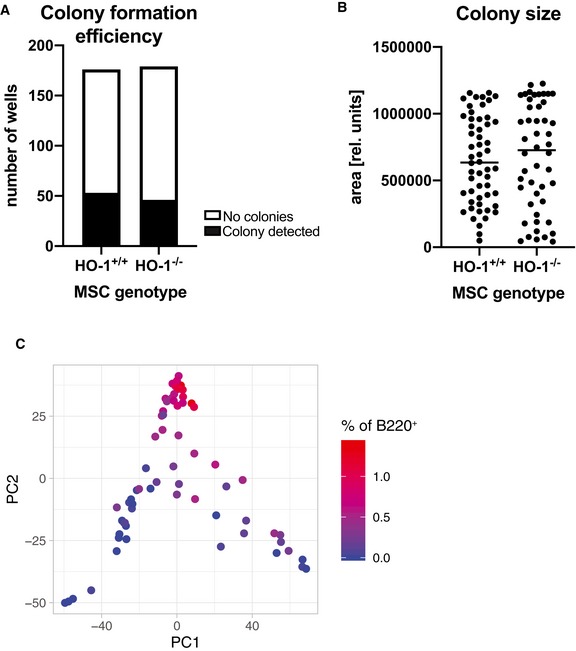
Characteristic of colonies formed by HSCs co‐cultured with HO‐1^−/−^ or HO‐1^+/+^ MSCs A, B(A) Colony formation efficiency and (B) the size of the formed colonies did not differ between groups.CFrequency of B220^+^ cells among analyzed colonies. 46–56 analyzed colonies/group.

Serial transplantation of HO‐1^+/+^ HSCs to HO‐1^−/−^ recipients revealed that HO‐1 deficiency affects HSCs in cell‐extrinsic manner (Fig 6). This effect could possibly be explained by HO‐1 deficiency in local BM niche or general systemic effect linked to microcytic anemia that was previously evidenced in HO‐1^−/−^ mice. To verify whether mesenchymal component of the local HO‐1‐deficient niche contributes to LT‐HSC dysfunction in HO‐1^−/−^ mice, we established *in vitro* cultures of mesenchymal stromal cells (MSCs) and co‐cultured them with LKS CD150^+^CD48^−^ HSCs isolated from HO‐1^+/+^GFP^+^ mice (Fig 8A). After 1 week of co‐culture, we sorted the single GFP^+^ HSCs for colony formation assay in serum‐free differentiation media (supplemented with TPO, IL‐3, SCF, and EPO) (Fig 8A). The first colonies could be detected by day 8 of differentiation, while the second group of colonies were first visible at day 12. We observed significantly less colonies that were cultured with HO‐1^−/−^ stromal cells among colonies appearing at day 8, while more among colonies appearing at day 12 (Fig 8B). This indicates that co‐culture of wild‐type HSCs with HO‐1^−/−^ stromal cells affects kinetic of growth of the HSC‐derived colonies. The total efficiency of colony formation did not differ between compared groups (HO‐1^+/+^ MSC—53/176; HO‐1^−/−^ MSC—46/179, Fig EV4A), as well as the size of the colonies (Fig EV4B).

After 14 days of colony formation, we analyzed the phenotype of cells in colonies by flow cytometry (stained with CD11b, Gr1, B220, and CD3). We observed that majority of cells within the colonies showed myeloid phenotype (CD11b^+^Gr1^−^ or CD11b^+^Gr1^+^), small fraction showed B220 expression, and some of the cells did not express any of the analyzed markers. This population, named here as Lineage^−^ (Lin^−^), possibly represents still undifferentiated cells or cells of lineage not included in our analysis, e.g., erythroid. Based on our initial gating, we performed PCA and then unsupervised k‐mean clustering that allowed us to distinguish four clusters of colonies (Fig 8C). Cluster 1 and cluster 2 represent colonies with high content of CD11b^+^Gr1^−^ cells (Fig 8D), while cluster 3 consists of colonies with predominant CD11b^+^Gr1^+^ cells (Fig 8E). Cluster 4 includes colonies with highest frequency of cells without expression of analyzed markers (Fig 8F) and B220^+^ cells (Fig EV4C); however, frequency of B220^+^ was generally low (below 1.5%, Fig EV4C). Then, we analyzed whether the colonies formed by the HSCs co‐cultured with HO‐1^+/+^ or HO‐1^−/−^ MSCs were differentially distributed among clusters (Fig 8G). We found that colonies derived from HSCs co‐cultured with HO‐1^−/−^ MSCs were significantly enriched in cluster 4 (Fig 8H).

Thus, the performed *in vitro* colony formation assay showed that co‐culture of HSCs with BM MSCs from HO‐1^−/−^ mice affected the kinetic of growth and the differentiation of colonies they formed. Altogether, this might indicate that lack of HO‐1 in mesenchymal component of the BM niche is at least in part responsible for the phenotype of LT‐HSCs observed in HO‐1^−/−^ mice.

Nevertheless, *in vitro* assays are limited in representation of the *in vivo* niche biology. Therefore, we checked whether we can reverse the phenotype of LT‐HSCs in HO‐1^−/−^ mice by normalization of function of mesenchymal cells *in vivo*. We showed that HO‐1^−/−^ mice have decreased number of CARs in BM that produced less SDF‐1α (Fig 3A and C). As shown previously by others, SDF‐1α can be upregulated in the mesenchymal cells by Noggin 47. Thus, we treated mice with Noggin for 14 days and checked if we can rescue the LT‐HSC phenotype. However, in our protocol Noggin treatment did not upregulate SDF‐1α mRNA neither in HO‐1^+/+^ nor in HO‐1^+/+^ mice (Fig 8I) and did not affect the number (Fig 8J) and cell cycle (Fig 8K) of LT‐HSCs in HO‐1^−/−^ mice.

### Serial bleeding of HO‐1^+/+^ mice does not induce LT‐HSC phenotype observed in HO‐1^−/−^ mice

HO‐1^−/−^ mice were originally described to develop microcytic anemia due to iron deficiency 31. We checked whether the phenotype of LT‐HSCs observed in HO‐1^−/−^ mice is the result of the compensation of the systemic anemia. To this aim, we serially bled HO‐1^+/+^ mice each third day for 12 and 2 days after last bleeding analyzed the hematological parameters as well as frequency, cell cycle, and transcriptome of LT‐HSCs.

**Figure EV5:**
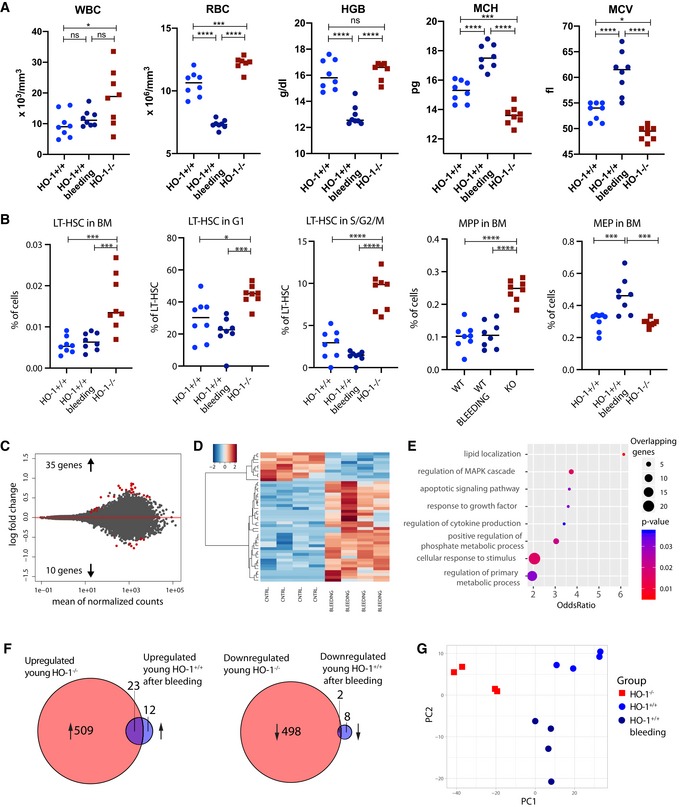
Induction of anemia by serial bleeding does not induce LT‐HSC phenotype observed in HO‐1^−/−^ mice AComparison of selected blood morphology parameters of HO‐1^−/−^ mice with HO‐1^+/+^ that were bled. *P < 0.05, ***P < 0.001, ****P < 0.0001, two‐tailed unpaired *t*‐test.BComparison of frequency and cell cycle of LT‐HSCs and frequency of MPPs and MPPs from bled HO‐1^+/+^ mice with LT‐HSCs from HO‐1^−/−^ mice. *P < 0.05, ***P < 0.001, ****P < 0.0001, two‐tailed unpaired *t*‐test.C, DRNA‐seq analysis revealed 45 differentially regulated genes in LT‐HSCs from bled HO‐1^+/+^ mice vs. LT‐HSCs from control HO‐1^+/+^ mice. Color key represents gene expression (as *z*‐score among row).EGSEA among GOBP annotations based on differentially regulated genes.FComparison of overlapping genes that were differentially expressed in LT‐HSCs from young HO‐1^−/−^ mice with genes that were differentially expressed in LT‐HSCs from bled HO‐1^+/+^ mice.GPCA based on genes differentially expressed in LT‐HSCs from both young HO‐1^−/−^ and bled HO‐1^+/+^ mice.

Analyzed young HO‐1^−/−^ mice have decreased MCH and MCV parameters that are indicators of microcytic anemia; however, hemoglobin level is not altered, and RBCs are even slightly elevated (Fig EV5A). This may indicate that young HO‐1^−/−^ mice can initially compensate the iron deficiency. Bleeding of wild‐type mice resulted in significant anemia demonstrated by reduced RBC and hemoglobin levels, with elevated MCH and MCV (Fig EV5A). The WBC levels were higher in HO‐1^−/−^ mice but were not altered by bleeding (Fig EV5A). Moreover, bleeding did not induce LT‐HSC phenotype observed in HO‐1^−/−^ mice: frequency and cell cycle status of LT‐HSCs in bled mice were not altered like in LT‐HSC HO‐1^−/−^ mice (Fig EV5B). The bleeding did not alter MPP frequency either, but significantly expanded the MEP progenitors (Fig EV5B).

Next, we analyzed whether bleeding of HO‐1^+/+^ mice can induce transcriptome changes in LT‐HSCs that characterize LT‐HSCs from HO‐1^−/−^ mice. To this aim, we parallelly processed LT‐HSCs from control HO‐1^+/+^ mice, bled HO‐1^+/+^ mice and HO‐1^−/−^ mice, and made RNA‐seq analysis. Consistently, we observed 1,032 differentially expressed genes (FDR < 0.1) in HO‐1^−/−^ mice compared to control HO‐1^+/+^ mice. In contrast, bleeding of HO‐1^+/+^ resulted only in 45 differentially regulated genes that distinguished LT‐HSCs from bled and control HO‐1^+/+^ mice (Fig EV5C and D, Dataset EV7). These genes were enriched in general GOBP terms connected with response to growth factor and stimulus, cytokine production, and apoptotic pathway (Fig EV5E). Comparison of genes significantly dysregulated by bleeding with genes dysregulated in young HO‐1^−/−^ mice revealed that 25 of 45 genes dysregulated by bleeding overlaps with genes dysregulated in HO‐1^−/−^ mice (Fig EV5F, Dataset EV8). While this represents over half (55.6%) of the dysregulated genes in bleeding group, PCA based on all differentially expressed genes in both groups did not reveal similarity of the transcriptome of LT‐HSCs from HO‐1^+/+^ bled mice with transcriptome of LT‐HSCs from HO‐1^−/−^(Fig EV5G). Altogether, we were not able to mimic phenotype of LT‐HSCs from HO‐1^−/−^‐deficient mice by serial bleeding and induction of anemia.

## Discussion

We demonstrated that HO‐1 is highly expressed in the BM niche. HO‐1 deficiency affects the BM niche and is associated with premature exhaustion of HSCs. Expression of HO‐1 in the niche decreases with age. In accordance, LT‐HSCs in HO‐1‐deficient mice reveal impaired function and aged‐like transcriptional signature. Notably, the impaired function and alterations of HO‐1^−/−^ LT‐HSCs can be reversed by serial transplantation into young wild‐type HO‐1^+/+^ recipients.

In this study, we characterized the aging of HSCs not only by known hallmarks but also by global transcriptional profiling (Fig 5). We found transcriptional similarity between LT‐HSCs isolated from young HO‐1^−/−^ mice and LT‐HSCs isolated from physiologically aged wild‐type mice of matching genotypic strains. This similarity pertains to genes involved in broad kinds of processes: cell cycle, metabolism, cell adhesion, DNA repair, and cytokine signaling (Fig 5H). The altered expression of cell cycle genes in old LT‐HSCs is consistent with previous work on the transcriptome of aged HSCs 48, although we did not observe an increase in the percentage of LT‐HSCs in the G1 phase in old wild‐type mice, as reported previously 48. Nevertheless, we observed that LT‐HSCs from young HO‐1^−/−^ mice have an increased fraction of cells in G1 and S/G2/M phases compared to young HO‐1^+/+^ (Fig 4F), while LT‐HSCs from older HO‐1^−/−^ mice have more cells in G1 phase compared to older HO‐1^+/+^, but not in S/G2/M phases (Fig 4H). The latter results can be explained by generally decreased proliferation capability of old HSCs as shown previously 2.

The other characteristic of aging LT‐HSCs is the dysregulation of metabolic checkpoints. Sirt‐7 has been proposed as one of the metabolic regulators that are decreased in aged LT‐HSCs 49. Consistently, we observed significantly reduced expression of Sirt‐7 in aged wild‐type LT‐HSCs and similar trends in young HO‐1^−/−^ LT‐HSCs. Moreover, we found that genes regulating pyruvate metabolism and maintaining glycolysis in HSCs are also decreased in old LT‐HSCs and young HO‐1^−/−^ LT‐HSCs (Fig EV3A).

In the present work, we did not use myeloid skewing as a marker of LT‐HSC aging. We previously showed that the myeloid bias in HO‐1^−/−^ mice is linked to HO‐1's role at the level of myelocytes 50. Given that HO‐1 deficiency causes the myeloid bias at the level of myelocytes, aging of LT‐HSCs in HO‐1^−/−^ animals could not be judged by increased output of mature myeloid cells.

Finally, while we observed the broad similarities of transcriptome of HSCs from young HO‐1^−/−^ mice to HSCs from old physiologically aged HO‐1^+/+^ mice, some features differ between these two groups. For example, it is known that in old mice, the HSC fraction expands but the MPP fraction does not 51. In contrast, in young HO‐1^−/−^ animals we observed expansion of both HSCs and MPPs (Fig 4B and D).

Two previous papers assessed the role of HO‐1 in HSCs; however, they did not focus on steady‐state function of the niche. Cao and colleagues showed that HO‐1^+/−^ mice had normal hematopoiesis in steady‐state conditions, but showed impaired long‐term hematopoietic activity upon 5‐FU‐induced myelotoxic injury and serial transplantation 52. Other group revealed that HO‐1 inhibition may be a strategy to improve mobilization of HSCs 53 and that cultured stromal cells produce less SDF‐1α and present impaired ability to support HSPC adhesion 53. While, the conclusions of these papers are in line with our work, here we describe a broader role of HO‐1 on strictly defined HSCs. For the first time, we showed that HO‐1 should be considered not only as intrinsic enzyme activated in various hematopoietic cells in stress conditions, but also as crucial extrinsic regulator of strictly defined HSCs in steady‐state conditions.

Our transcriptional data indicating the aged‐like phenotype of HO‐1^−/−^ LT‐HSCs have been followed by functional *in vivo* studies. We have proposed novel schemes of HSC transplantation to test the role of the cell‐extrinsic factors in HSC aging (Fig 6B). Namely, we transplanted HSCs from wild‐type mice into recipients deficient in HO‐1 and then performed secondary transplant of the same number of only donor‐derived BM cells to young wild‐type animals. This allowed us to prospectively check how aging of LT‐HSCs in HO‐1^−/−^ mice affects their function and to demonstrate cell‐extrinsic reversal of the observed phenotype. We believe that our experimental strategy may be used to study the role of other factors.

While HSCs’ aging and exhaustion are well described, the reversal of impaired HSCs’ function has remained elusive. Here, we showed that the phenotype of LT‐HSCs from young HO‐1^−/−^ mice can be reversed by the wild‐type niche. Upon primary transplantation into wild‐type recipients, HO‐1^−/−^ LT‐HSCs demonstrated impaired reconstitution even after 16 weeks, without any signs of a reversal of declined function (Fig 4N). However, as we showed, LT‐HSCs isolated from young HO‐1^−/−^ mice have altered gene expression profiles (Fig 5) and cell cycle status (Fig 4F). Therefore, it is possible that they lost the competition for niches with residual wild‐type LT‐HSCs and LT‐HSCs within the supporter cells at the initial stages after transplantation, and they are not able to rebound the contribution to blood production afterward. Indeed, we observed lower chimerism among the HSPC fraction after transplantation of HO‐1^−/−^ HSCs (Fig 6D). Finally, while HO‐1 is barely detectable in HSCs in steady state, it might be expressed upon stress conditions 52. Thus, lack of HO‐1 during the transplantation stress may affect their viability. To avoid these equivocal interpretations, we did serial transplantation of the same number of donor‐derived cells from primary recipients into the secondary recipients. By this way, we provided the same conditions for the compared groups. In such experimental settings, the secondary HO‐1^+/+^ niche fully restored the reconstitution potential of HO‐1^−/−^ HSCs to equal that of control HO‐1^+/+^ HSCs (Fig 7C). This implies that the young HO‐1^+/+^ niche can reverse the impaired reconstitution capacity of HO‐1^−/−^ HSCs and that the induction of HO‐1 in HSCs during transplantation is not a limiting factor in the assay.

A recent study demonstrated that reduced expression of osteopontin (*Spp1*) in BM stroma regulates aging of HSCs, and restoring Spp1 levels attenuates aged phenotype of HSCs 54. This is in line with our study indicating that the young niche has the potential to reverse some of the aging‐related features of HSCs. However, we did not observe altered expression of *Spp1* in HO‐1^−/−^ ECs or CARs (Datasets EV1 and EV2). Instead, we demonstrate that specific niche populations of CARs and ECs produce reduced amounts of SDF‐1α and precursor form of TGF‐β1, respectively. As SDF‐1α and TGF‐β1 are crucial for HSC quiescence 25, 26, 55, 56, we suppose that their decreased production in HO‐1^−/−^ niche may contribute to the observed phenotype of HO‐1^−/−^ LT‐HSCs.

Along with demonstrating that function of HO‐1^−/−^ LT‐HSCs can be restored by HO‐1^+/+^ niche, we checked whether the transcriptional profile of HO‐1^−/−^ LT‐HSCs was also reversed. Naturally, this comparison has limitations, as transplanting the cells twice alone induces their aging 57 as well the duration of experiment can cause significant transcriptional changes. Thus, it is not unreasonable that a large number of genes that were changed in LT‐HSCs from serial transplantation overlap with genes that are differentially expressed between the impaired young HO‐1^−/−^ LT‐HSCs and the wild‐type young HO‐1^+/+^ LT‐HSCs (Fig 7F and G). Although these overlapping genes had to be excluded from our analysis given the confounding effect of transplantation itself, we still demonstrated that transplantation of HO‐1^−/−^ LT‐HSCs into the HO‐1^+/+^ niche reverses the altered expression of the other non‐overlapping, differentially expressed genes (Fig 7I).

Our findings suggest that reversing the age‐related decline in HSC function may be achieved by enhancing the HO‐1 activity in the niche. Potential clinical translation of HO‐1 function in the HSC niche should take into consideration: (i) monitoring HO‐1 expression as a diagnostic for bone marrow age and niche dysfunction, (ii) screening for HO‐1 polymorphisms in hematological disorders, and (iii) targeting HO‐1 activity and expression by pharmacological induction. In humans, the activity of HO‐1 is linked to promoter polymorphisms 58, 59. Longer promoters with more GT repeats are linked to lower expression of HO‐1, while shorter GT repeats lead to higher levels of HO‐1 34, 59. We showed previously that in human endothelial cells, the shorter alleles of HO‐1 promoter provide better response to oxidative stress, VEGF‐induced proliferation, and lower production of inflammatory cytokines 34. Given the crucial role of endothelial cells in the HSC niche, people with longer HO‐1 promoter variant and impaired function of endothelial cells might have increased risk of accelerated aging of the HSCs. Secondly, some kinds of porphyrins, e.g., cobalt protoporphyrin IX, induce expression of HO‐1 *in vivo* and might be considered as potential treatment in case the function of HSCs is impaired. Taken together, this study suggests an important role of HO‐1 also in the human HSC niche and calls for future studies to pursue the clinical applications of our findings.

While our functional experiments demonstrate the cell‐extrinsic role of HO‐1 in regulation of HSC exhaustion, the question remains whether this cell‐extrinsic role is mediated by local HSC niche or by systemic factors, e.g., linked to iron deficiency and microcytic anemia in HO‐1^−/−^ mice. We demonstrated high HO‐1 expression in steady‐state conditions in BM niche, characterized which populations express HO‐1, how the expression changes in aging, and demonstrated that lack of HO‐1 in niche cells dysregulates production of hematopoietic fractions. Additionally, we showed that co‐culture of HSCs with HO‐1^−/−^ stromal cells affects their kinetic of growth and differentiation of the derived colonies (Fig 8). However, the differences in *in vitro* co‐culture assay showed delayed and impaired differentiation of HSCs on HO‐1^−/−^ stromal cells what does not fully concur with *in vivo* observations indicating accelerated proliferation and exhaustion of HSCs in HO‐1^−/−^ mice. This can be possibly explained by the fact that *in vitro* models based solely on stromal cells cannot fully recapitulate the complex physiological HSC niches. Nevertheless, the *in vitro* assay demonstrated that lack of HO‐1 in stromal cells affects the phenotype of LT‐HSCs.

In contrast, induction of anemia by serial bleeding does not lead to the phenotype of LT‐HSCs observed in HO‐1^−/−^ mice (Fig EV5).

Given our findings on role of HO‐1 in BM niche, it seems likely that lack of HO‐1 in the niche cells triggers the phenotype of LT‐HSCs in HO‐1^−/−^ mice. However, we cannot exclude that other systemic factors intertwine with HO‐1 role within the BM niche and together contribute to dysregulation of HSCs in HO‐1^−/−^ mice.

While our HO‐1^−/−^ mice represent the constitutive knock‐out model, the observed phenotype of HSC in BM of young mice is not likely caused by developmental alterations and colonization of BM by HSCs, as existing data indicate no HO‐1 expression in fetal liver HSCs 60.

In conclusion, we identified HO‐1 as a new factor in the BM niche. The expression of HO‐1 in the BM niche decreases in aged animals, and extrinsic HO‐1 deficiency is linked to premature exhaustion of LT‐HSCs. This study suggests that modulation of HO‐1 activity in BM niche may be used to restore the impaired function of exhausted HSCs.

## Materials and Methods

### Animals

All animal procedures and experiments were performed in accordance with national and European legislations, after approval by the First or Second Local Ethical Committee on Animal Testing at the Jagiellonian University in Krakow (approval numbers: 56/2009, 113/2014, 120/2019).

The HO‐1^−/−^ mice and HO‐1^−/−^ mice constitutively expressing GFP (HO‐1^−/−^GFP^+^) were kindly provided by Dr. Anupam Agarwal from University of Alabama at Birmingham. HO‐1^−/−^ strain poorly breeds on pure C57/Bl6 background (5.1% of expected HO‐1^−/−^ pups) and therefore is maintained on mixed C57/Bl6 × FVB background (20.1% of expected HO‐1^−/−^ pups, when HO‐1^−/−^ males are crossed with HO‐1^+/−^ females) 61. While there is increased perinatal mortality of HO‐1^−/−^ animals, we did not observe decreased lifespan among HO‐1^−/−^ mice that were born alive. The weight of HO‐1^−/−^ mice during aging in steady‐state conditions did not differ, and HO‐1^−/−^ mice did not reveal acute sickness or abnormalities.

For bleeding experiments, mice were serially bled—every third day, total of four bleedings. Three days after last bleeding, mice were euthanized and material was collected. Blood (~ 100–200 μl) was collected by submandibular vein puncture.

Recombinant murine Noggin (Peprotech) was administered i.p. 500 μg/kg, every second day for 2 weeks, and 2 days after last dose, mice were euthanized and material was collected.

To assure relative homogeneous background, all HO‐1^+/+^ controls were C57/Bl6xFVB littermates from the same breeders used to obtain HO‐1^−/−^ mice. HO‐1^fl/fl^;LysM‐Cre were analyzed at Center of Translational Research, Medical University of Vienna, Austria.

### Flow cytometry analysis and cell sorting

Flow cytometry analysis was done on LSR II and LSRFortessa cytometers (BD Sciences). Cell sorting was done on MoFlo XDP cell sorter (Beckman Coulter). The populations used in studies were defined as follows: HSCs—LKS CD150^+^CD48^−^, LT‐HSCs—LKS CD150^+^CD48^−^CD34^−^, ST‐HSCs—LKS CD150^+/mid^CD48^−^CD34^+^, ST‐HSCs II—LKS CD150^−^CD48^−^CD34^+^, MPP—LKS CD150^−^CD48^+^, ECs—CD45^−^Ter119^−^PDGFRα^−^CD31^+^Sca‐1^+^, CARs—CD45^−^Ter119^−^PDGFRα^+^ CD31^−^Sca‐1^−^, and PαS—CD45^−^Ter119^−^PDGFRα^+^CD31^−^Sca‐1^+^. Following antibody clones were used in the study: CD34 (clone RAM34, BD Biosciences), Sca‐1 (D7, BD Biosciences, eBioscience), c‐Kit (clone 2B8, eBioscience, USA), CD45 (clone 30F‐11, BD Biosciences), CD150 (clone TC15‐12F12.2, BioLegend), CD48 (clone HM‐48‐1, BioLegend), CD45R‐PE (clone RA3‐6B2, BD Biosciences), Ly6G and Ly6C‐PE (clone RB6‐8C5, BD Biosciences), TCRγδ‐PE (clone GL3, BD Biosciences), TCRβ‐PE (clone H57‐597, BD Biosciences), CD11b‐PE (clone M1/70, BD Biosciences), Ter119‐PE (clone TER119, BD Biosciences), CD3 (clone 17A2, BD Biosciences), F4/80 (clone BM8, eBioscience), MHCII (clone M5/114.15.2), CD31 (clone MEC13.3, BD Biosciences), CD140α (clone APA5, eBioscience), Ki67 (clone B56), HO‐1 (SPA894, Enzo Life Sciences), SDF‐1α‐APC (clone 79018, R&D), LAP(TGF‐β1)‐Alexa647 (clone TW7‐16B4, BD Biosciences), and SCF‐biotinylated (#BAF455, R&D). p‐γH2aX (Ser139) staining (clone JBW301, Millipore) was done using IntraSure Kit—after surface antigen staining, 200 μl of Reagent A was added, followed by 4 ml BD FACS Lysing Solution. p‐γH2aX was added in Reagent B, for 45 min RT, diluted 500×. Incubation times were chosen according to IntraSure Kit manual. Goat anti‐Mouse Alexa 488 H + L dil. 1/400 (Molecular Probes) was used for detection.

Intracellular staining for flow cytometry analysis of HO‐1 and γH2aX was done using IntraSure Kit (BD Biosciences). Analysis of intracellular cytokines by flow cytometry was done using Fix/Perm Kit (BD Biosciences) according to the provided instructions with GolgiStop protein transport inhibitor, added to the enzyme for bone marrow digestion. Analysis of the cell cycle and measurement of the ATP content were done according to the protocols described in detail previously 62, 63.

### Comet assay

Alkaline comet assay was performed with Trevigen CometAssay Kit according to the manufacturer's protocol. In brief, LT‐HSCs from young 10‐ to 12‐week‐old HO‐1^−/−^ and HO‐1^+/+^ mice were sorted into small volume of PBS, embedded in Comet LMAgarose and transferred onto single CometAssay HT Slide. The slide was then incubated in 4°C for 30 min to enhance gelling process and placed in lysis solution for an overnight incubation in 4°C. Next, samples were treated with freshly made alkaline unwinding solution for 20 min RT and subjected to electrophoresis in alkaline conditions with an adjustment for electrophoresis units other than supplied by Trevigen (4°C, 1 V/cm, 300 mA, 30 min). After electrophoresis, slide was washed twice in dH_2_O followed by single 70% ethanol wash and dried in 37°C. Samples were stained with SYBR Gold and imaged. Analysis was performed on blinded files with CometScore software (TriTek), which calculated values for percent of Tail DNA and Olive Tail Moment. Statistical significance was calculated using unpaired Student's *t*‐test in GraphPad Prism Software.

### Immunohistochemistry

Tibias and femurs were fixed in 4% PFA with 10% EDTA for 4–8 h on ice in 4°C while rotating, followed by overnight incubation in 20% EDTA in 4°C. Next, the bones were put to sucrose solutions in PBS: 10%—2 h, 20%—2 h, and 30%—overnight in 4°C. Next, the excess of sucrose was removed, and bones were embedded in Tissue‐Tek Oct media (VWR) and frozen in dry‐ice‐cooled isopentane (Sigma). 70‐μm‐thick longitudinal section of bones were cut on cryostat (Leica), blocked with 5% goat or donkey serum and 3% BSA, and stained overnight with primary antibodies in 4°C, followed by 1‐h staining with secondary antibodies in room temperature. Primary antibodies used for immunohistochemistry include HO‐1 (cat. SPA894, Enzo Life Sciences), CD31 (clone MEC13.3, BD Biosciences), endomucin (clone V.7C7), Sca‐1 (cat. AF1226, R&D), SDF‐1α (cat. sc‐6193, Santa Cruz Biotechnology), and CD140β (clone APB5, eBioscience). Secondary antibodies used in the study include donkey F(ab’)2 fragments (Jackson ImmunoResearch) and donkey or goat whole IgG fragments (Molecular Probes) conjugated with Alexa Fluor dyes or Cy3 dyes. Imaging was done on LSM780 confocal microscope (Zeiss) and analyzed in ImageJ software.

### MSC cultures and *in vitro* colony assay

The primary cultures were isolated from femurs and tibias using enzymatic digestion, and after initial passages, CD45^+^ cells were excluded by magnetic sorting as described in detail in Ref. 64. The differentiation media was based on StemSpan SFEM (Stem Cell Technologies), supplemented with 20% of BIT 9500 Serum Substitute (Stem Cell Technologies), and cytokines: murine stem cell factor (mSCF, Peprotech), human thrombopoietin (hTPO, Peprotech), murine interleukin‐3 (mIL‐3, Peprotech), and human erythropoietin (hEPO, Sigma‐Aldrich), all at the concentration of 20 ng/ml as described in details in Ref. 65.

### Bone marrow transplants and chimerism analysis

Mice were irradiated with Cs‐137 source with total dose of 900 cGy for transplants presented in Figs 4N and 7A, and 950 cGy for transplants presented in Fig 6B in two equal split doses, 4 h apart. Twenty‐four hours after the last dose, mice were transplanted by tail vein injection with HSCs or donor‐derived whole BM cells in case of secondary transplantation. Sorted HSCs were transplanted with 10^5^ whole BM supporter cells, while in case of secondary transplantation, when donor‐derived whole BM cells were transplanted, the supported cells were not given.

The donor cells were distinguished by GFP expression. HO‐1^+/+^ HSC transplanted to HO‐1^+/+^ or HO‐1^−/−^ recipients (data in Fig 6B) was transduced with GFP coding lentiviral vectors according to previously published protocol 66. In brief, 360 HSCs were sorted to serum‐free expansion (StemSpan SFEM, Stem Cell Technologies), supplemented with 20% of BIT 9500 supplement (Stem Cell Technologies), 0.1% of EX‐CYTE supplement (Millipore), 5 μg/ml polybrene, 10 ng/ml murine stem cell factor (mSCF, Peprotech), and 100 ng/ml human thrombopoietin (hTPO, Peprotech), transduced with 100 MOI lentiviral vectors. After 12 h, all cells were transplanted together with 2 × 10^5^ whole BM supporter cells.

For transplantation of HO‐1^+/+^ or HO‐1^−/−^ HSCs (data in Fig 3N), we used mice strain HO‐1^+/+^ GFP^+^ and HO‐1^−/−^GFP^+^. For the assessment of the short‐term homing, 30,000 LSK isolated from C57BL/6‐Tg(UBC‐GFP)30Scha/J were transplanted per HO‐1^+/+^ or HO‐1^−/−^ recipient. Recipients were irradiated with 950 cGy in a single dose, 24 h prior to transplantation. The number of homed GFP^+^ cells was evaluated by 12 h after transplantation by flow cytometry using Trucount Tubes (BD) for absolute cell number evaluation.

The chimerism was checked by peripheral blood bleeding from retro‐orbital sinus or submandibular vein puncture. The percentage of GFP^+^ donor cells were checked among total blood cells (CD45^+^), or granulocytes (CD11b^+^Gr1^+^SSC^high^), B cells (CD11b^−^Gr1^−^B220^+^CD3^−^), and T cells (CD11b^−^Gr1^−^B220^−^CD3^+^). The positive recipients were defined as having at least 0.5% chimerism among granulocytes, what was above detection threshold based on GFP‐ mice.

### Transcriptome analysis by RNA‐seq

To prepare libraries from small number of cells, we used previously described Smart‐seq2 protocol 67. In case when 300–3,000 cells were sorted (analysis presented in Figs 2 and 4), RNA was isolated using the Single Cell RNA Purification Kit (Norgen Biotek). In case where 25–250 cells were available, the cells were sorted directly to lysis buffer (0.2% Triton X‐100 with RNase inhibitors), which was used for next steps of Smart‐seq2 protocol (data presented in Fig 7E–J). The quality of RNA and material during preparation of libraries was checked by Bioanalyzer. The samples were sequenced on NextSeq 500 (Illumina) with 75 bp single‐end reads, aligned to mm^10^ reference mouse genome by BWA or STAR mapping software (~ 85% mapping efficiency with ~ 10–20 million uniquely mapped reads/sample). Differential gene expression (DGE) analysis was done using DESeq2 package 68 and R software environment. We analyzed differentially expressed genes only between libraries prepared in the same experimental batch. In the experiment where limited number of cells was available (data presented in Fig 7E–J), we repeated sorting the control groups and library preparation to eliminate batch effect and equal drop‐out rate among libraries.

### Statistical analysis

Statistical analysis was conducted using GraphPad Prism software. Data are shown as mean ± SEM. When two groups were compared, two‐tailed unpaired *t*‐test was applied. In case when more than two groups were compared, one‐way or two‐way ANOVA with Sidak or Bonferroni post‐test was performed. Number of samples per group and number of independent experiments are described in the figure legends. Results were considered as significant for *P* < 0.05 (*P < 0.05, **P < 0.01, ***P < 0.001).

## Author contributions

Conceptualization: KS, AJ, JD. Formal analysis: KS, MZ, GSG. Investigation: KS, MZ, AS, WN, IS, MC, KB‐S, NK‐T, AK, EE, JK, SC. Methodology: EE, HE, VB. Writing of original draft: KS, AJ, AS, GSG, ILW. Supervision: AJ, JD.

## Conflict of interest

The authors declare that they have no conflict of interest.

## Supporting information



Appendix

Expanded View Figures PDF

Dataset EV1

Dataset EV2

Dataset EV3

Dataset EV4

Dataset EV5

Dataset EV6

Dataset EV7

Dataset EV8

Review Process File

## Data Availability

The raw sequencing data from this publication have been deposited to the SRA database. Individual files are described and can be accessed in BioProject database entry, accession no.: PRJNA562450. https://www.ncbi.nlm.nih.gov/bioproject/PRJNA562450.
