# Supplementary material for: Heme oxygenase‐1 deficiency triggers exhaustion of hematopoietic stem cells
Source: EMBO Rep. 2019 Dec 29;21(2):e47895. doi: 10.15252/embr.201947895 (PMC7001511; doi:10.15252/embr.201947895)
Supplement: Supplementary file 1 — Appendix [file EMBR-21-e47895-s001.pdf]

Appendix content:

Appendix Figure S1 Related to Figure 1 page 2

Appendix Figure S2 Related to Figure 5 page 3

Appendix Figure S3 Related to Figure 7 page 4

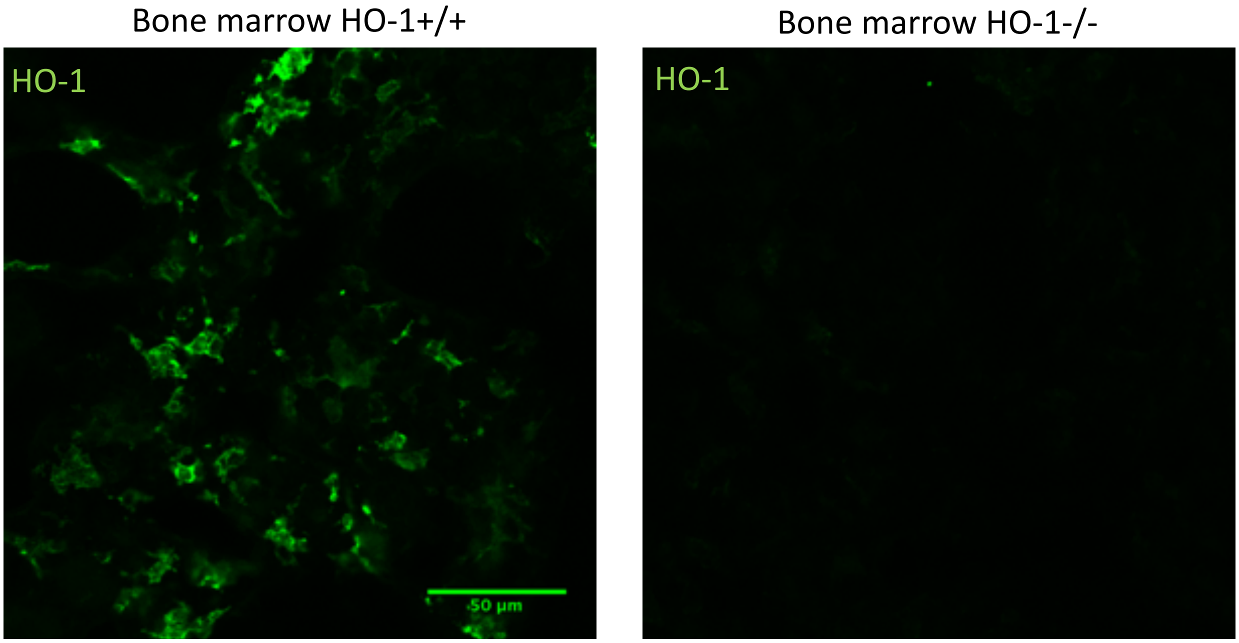

Appendix Figure S1 Related to Figure 1. Lack of staining with HO-1 antibody (clone SPA894) on bone marrow sections from HO-1$^{-/-}$ mice proves antibody specificity.

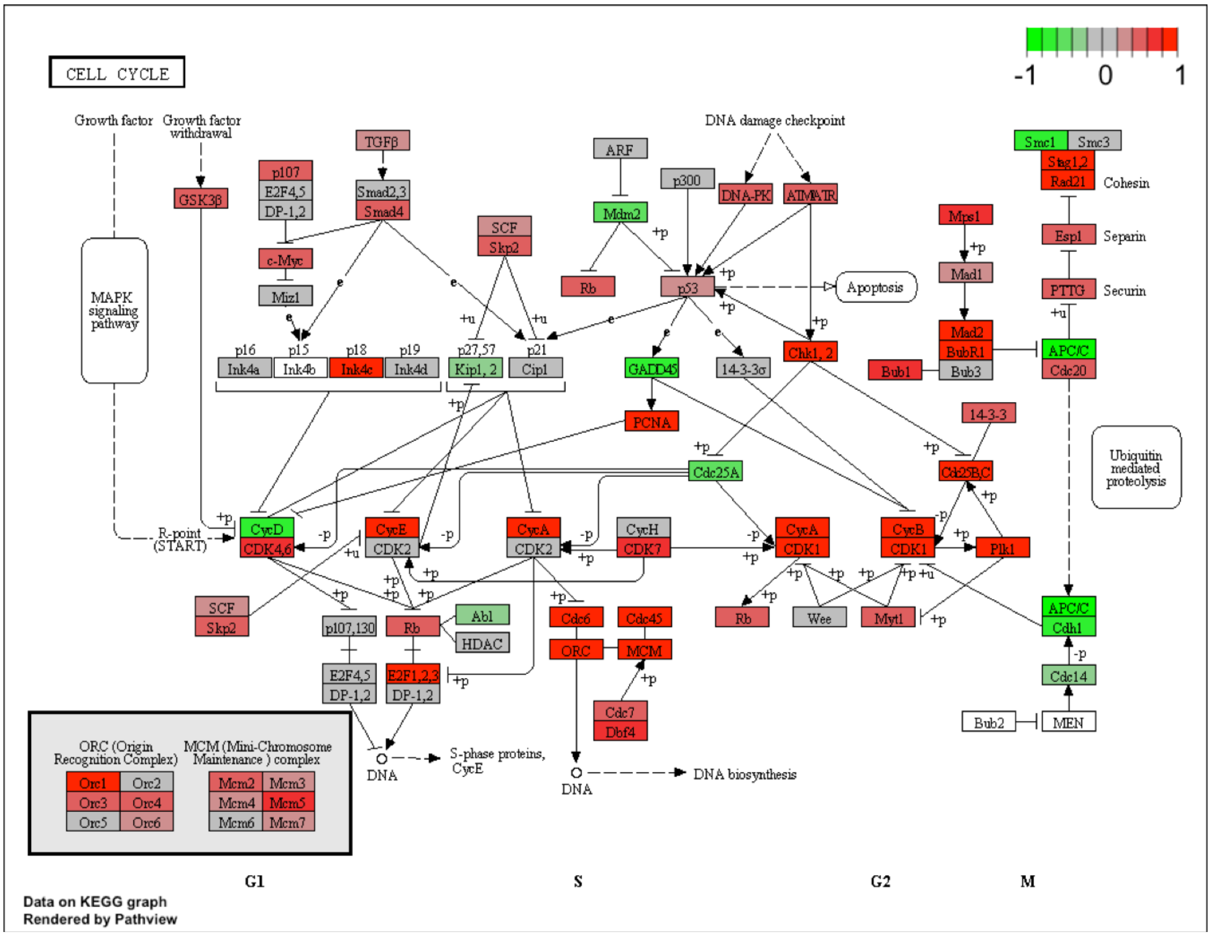

Appendix Figure S2 Related to Figure 5. KEEG pathway analysis showing the alterations among genes linked with cell cycle in young HO-1$^{-/-}$ LT-HSCs.

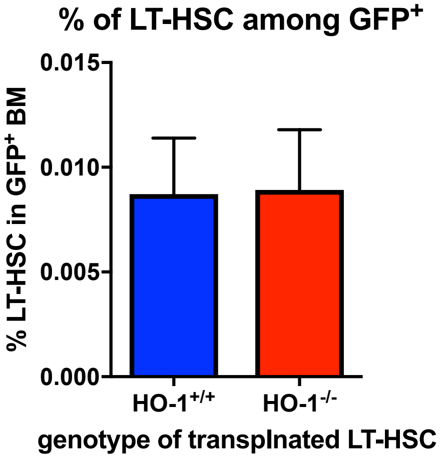

Appendix Figure S3 Related to Figure 7. Percentage of LT-HSC among all GFP+ donor derived BM cells in primary recipients, n = 7-8 mice/group.
