# Supplementary material for: Heme oxygenase‐1 deficiency triggers exhaustion of hematopoietic stem cells
Source: EMBO Rep. 2019 Dec 29;21(2):e47895. doi: 10.15252/embr.201947895 (PMC7001511; doi:10.15252/embr.201947895)
Supplement: Supplementary file 2 — Expanded View Figures PDF [file EMBR-21-e47895-s002.pdf]

Expanded View Figures

Figure EV1. Expression of HO-1 in BM niche (related to Fig 1).

A HO-1 is expressed by $CD31^+endomucin^+$ endothelial cells in metaphysis region of a tibia, scale bar 200 μm.
B HO-1 is expressed in sinusoids in diaphysis region, however, at lower levels, scale bar 100 μm.
C $PDGFR\beta^+$ stromal cells in diaphysis region of the bone express HO-1, scale bar 20 μm.
D Pericytes express HO-1. Part of the $HO\text{-}1^+$ pericytes express Sca-1 (#), while others express no or low levels of Sca-1 (*), scale bar 20 μm.
E HO-1 is expressed by $PDGFR\beta^+$ stromal cells. Part of $HO\text{-}1^+PDGFR\beta^+$ cells produce SDF-1α, scale bar 20 μm.

 © 2019 The Authors

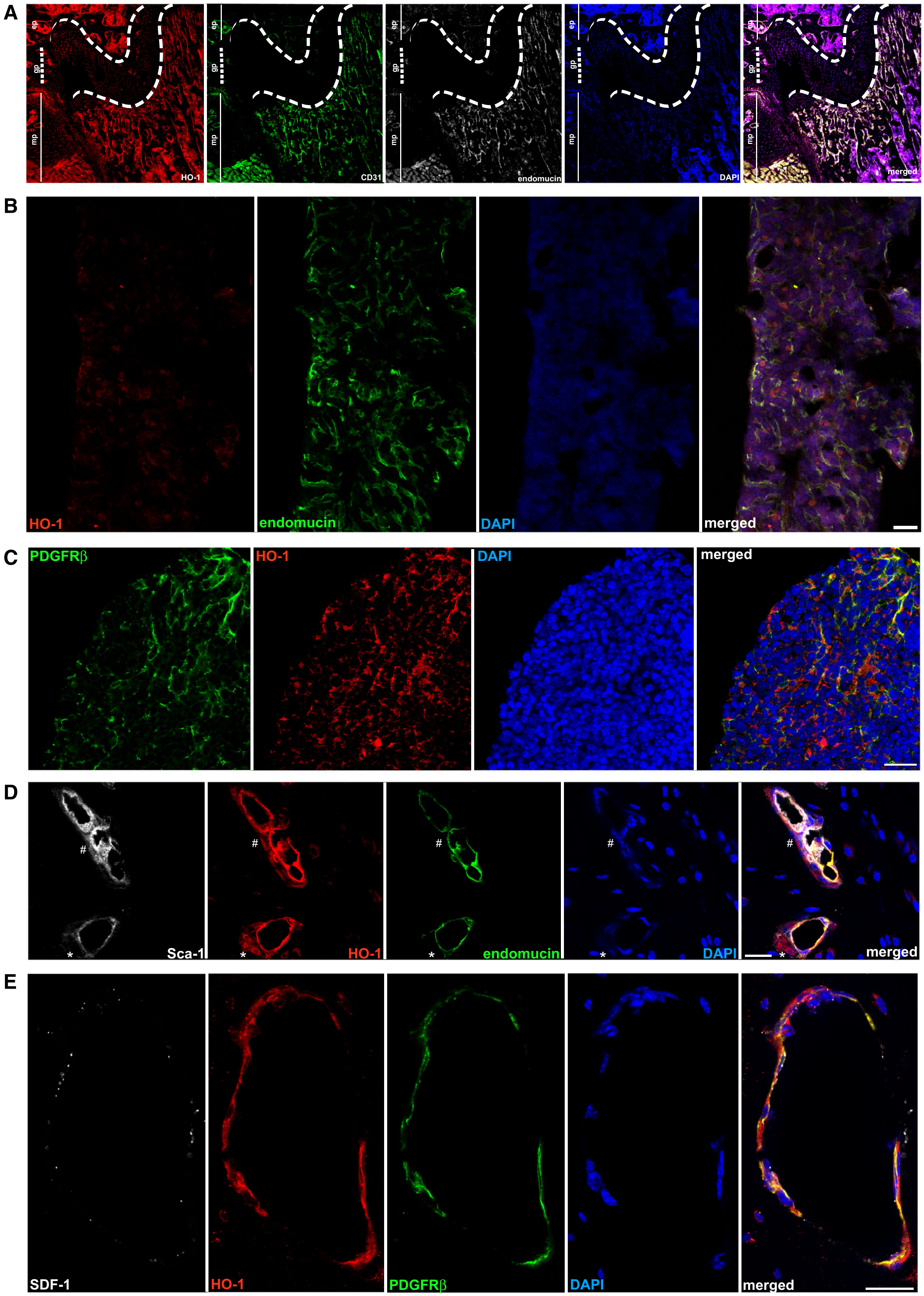
A
ep
gp
mp
HO-1
CD31
endomucin
DAPI
merged
B
HO-1
endomucin
DAPI
merged
C
PDGFRβ
HO-1
DAPI
merged
D
#
*
Sca-1
HO-1
endomucin
DAPI
merged
E
SDF-1
HO-1
PDGFRβ
DAPI
merged

Figure EV1.

Figure EV2. Frequency of hematopoietic stem and progenitors cells in HO-1$^{+/+}$ and HO-1$^{-/-}$ mice (related to Fig 4).

A Frequency of ST-HSC II population in young HO-1$^{+/+}$ and HO-1$^{-/-}$ mice. Two-tailed unpaired *t*-test.
B Frequency of MEP, EP, and GMP populations in young HO-1$^{+/+}$ and HO-1$^{-/-}$ mice. Two-tailed unpaired *t*-test.
C Frequency and total number of LT-HSCs, ST-HSCs, and MPPs in 12-month-old mice, n = 5 mice/group. Unpaired, two-tailed *t*-test.

© 2019 The Authors

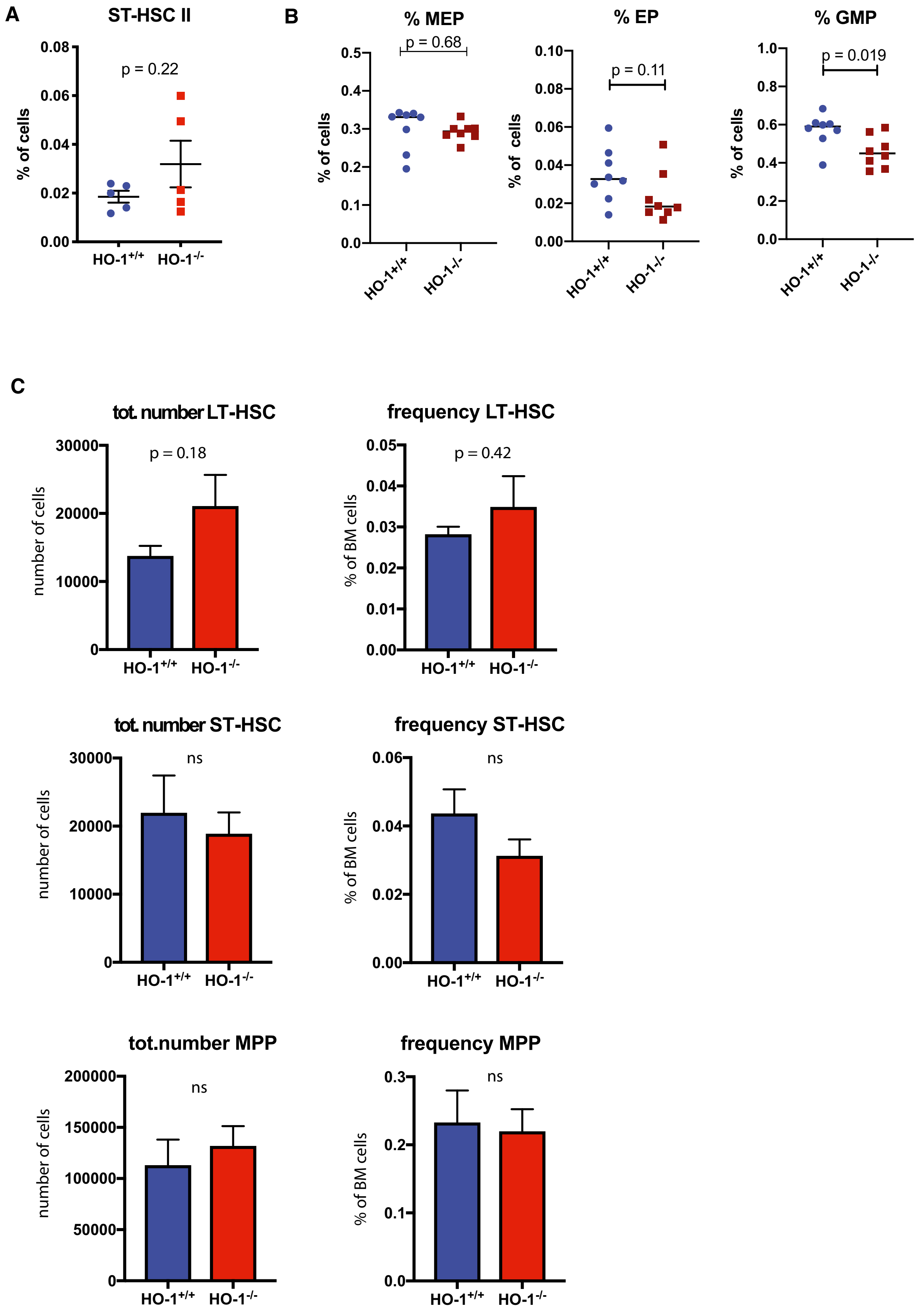
A
ST-HSC II
% of cells
p = 0.22
0.00 0.02 0.04 0.06 0.08
HO-1+/+ HO-1-/-
B
% MEP
p = 0.68
0.0 0.1 0.2 0.3 0.4 0.5
% EP
p = 0.11
0.00 0.02 0.04 0.06 0.08 0.10
% GMP
p = 0.019
0.0 0.2 0.4 0.6 0.8 1.0
% of cells
HO-1+/+ HO-1-/-
C
tot. number LT-HSC
number of cells
p = 0.18
0 10000 20000 30000
frequency LT-HSC
% of BM cells
p = 0.42
0.00 0.01 0.02 0.03 0.04 0.05
tot. number ST-HSC
ns
0 10000 20000 30000
frequency ST-HSC
ns
0.00 0.02 0.04 0.06
tot.number MPP
ns
0 50000 100000 150000 200000
frequency MPP
ns
0.0 0.1 0.2 0.3
HO-1+/+ HO-1-/-

Figure EV2.

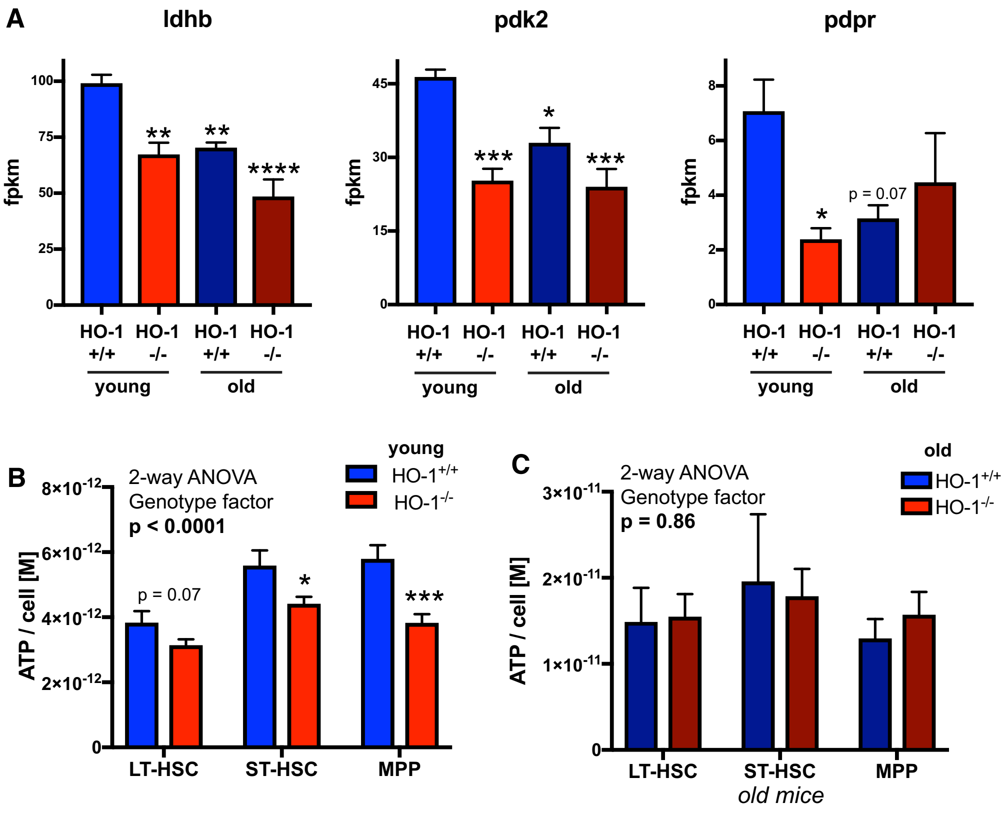

Figure EV3. Decreased expression of genes regulating pyruvate metabolism in young HO-1$^{-/-}$ LT-HSCs and old LT-HSCs is associated with lower ATP levels.

A Ldhb, Pdk2, and Pdpr are downregulated in young HO-1$^{-/-}$ LT-HSCs, but not in old HO-1$^{-/-}$ LT-HSCs. Analyzed by RNA-seq. Data are shown as mean ± SEM, four mice/group, *$P < 0.05$, **$P < 0.01$, ***$P < 0.001$, ****$P < 0.0001$ comparing to young HO-1$^{+/+}$ group, two-tailed unpaired *t*-test.

B, C ATP levels are (B) lower in young HO-1$^{-/-}$ LT-HSCs comparing to young HO-1$^{+/+}$ LT-HSCs, (C) but not in old HO-1$^{-/-}$ LT-HSCs comparing to old HO-1$^{+/+}$ LT-HSCs. ATP levels measured in two independent experiments. Data are shown as mean ± SEM, n = 8–18/group. *$P < 0.05$, ***$P < 0.001$, two-tailed unpaired *t*-test.

 © 2019 The Authors

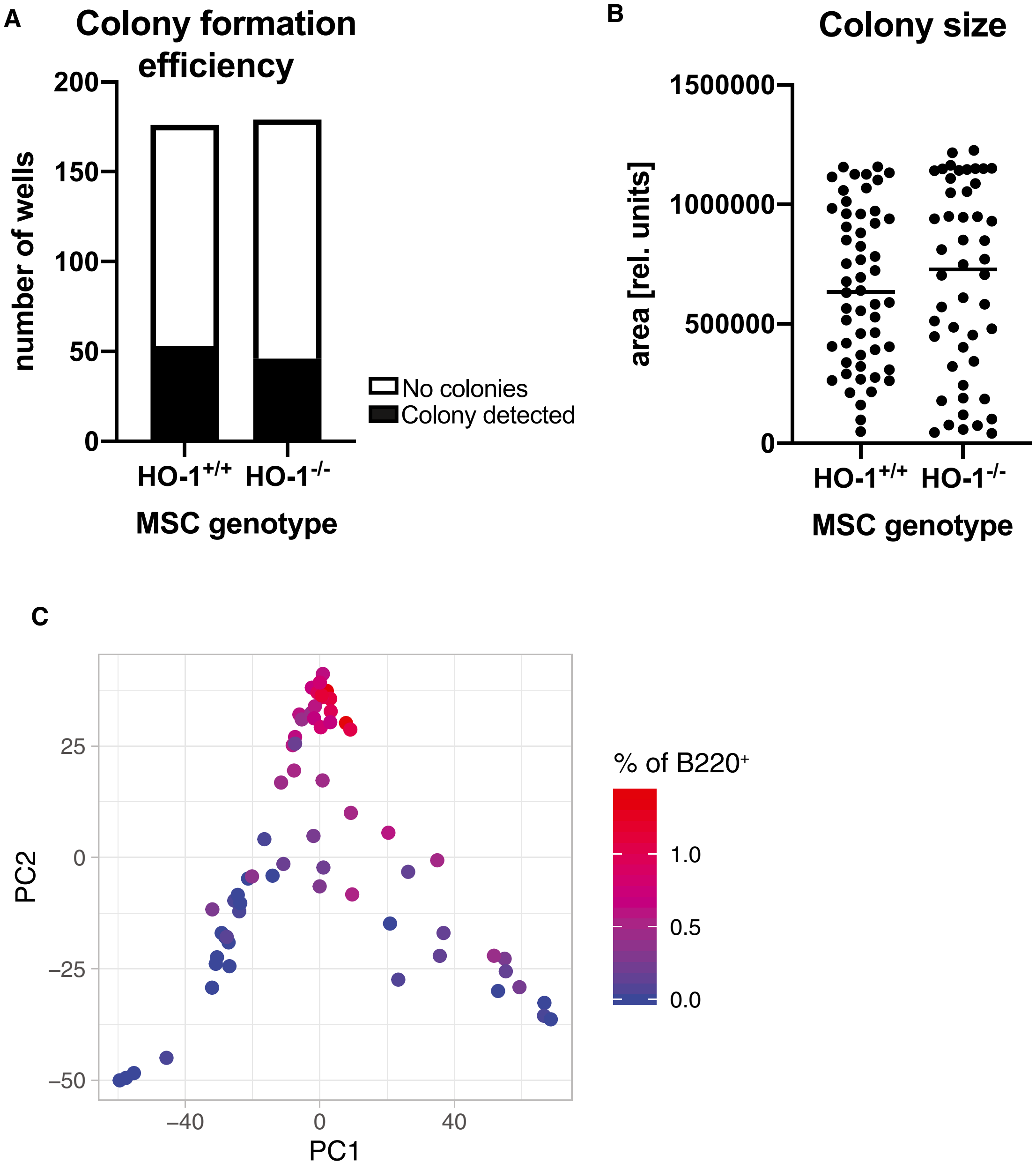

Figure EV4. Characteristic of colonies formed by HSCs co-cultured with HO-1$^{-/-}$ or HO-1$^{+/+}$ MSCs.

A, B (A) Colony formation efficiency and (B) the size of the formed colonies did not differ between groups.

C Frequency of B220^{+} cells among analyzed colonies. 46–56 analyzed colonies/group.

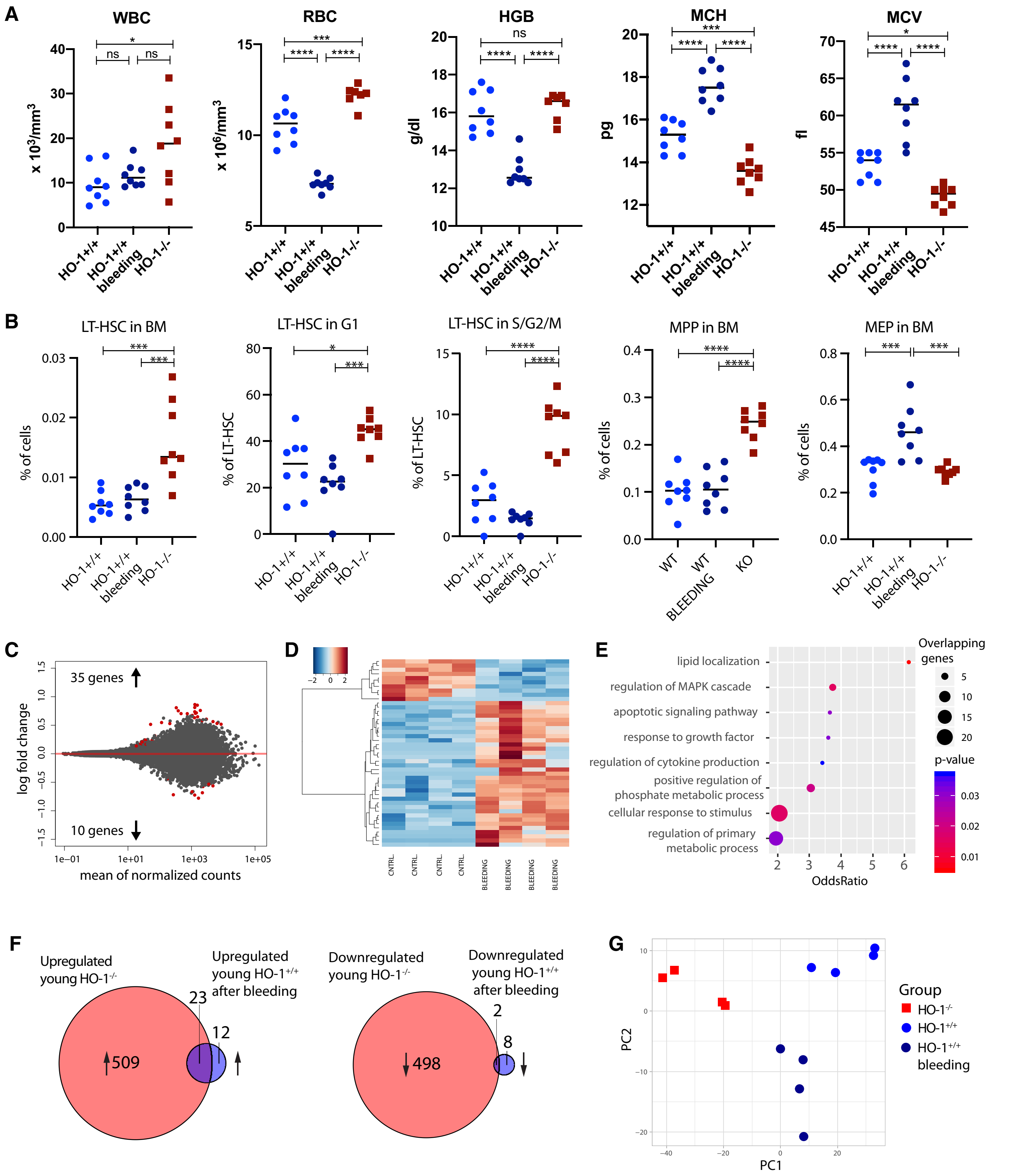

Figure EV5.

 © 2019 The Authors

Figure EV5. Induction of anemia by serial bleeding does not induce LT-HSC phenotype observed in HO-1$^{-/-}$ mice.

A Comparison of selected blood morphology parameters of HO-1$^{-/-}$ mice with HO-1$^{+/+}$ that were bled. *$P < 0.05$, ***$P < 0.001$, ****$P < 0.0001$, two-tailed unpaired *t*-test.

B Comparison of frequency and cell cycle of LT-HSCs and frequency of MPPs and MPPs from bled HO-1$^{+/+}$ mice with LT-HSCs from HO-1$^{-/-}$ mice. *$P < 0.05$, ***$P < 0.001$, ****$P < 0.0001$, two-tailed unpaired *t*-test.

C, D RNA-seq analysis revealed 45 differentially regulated genes in LT-HSCs from bled HO-1$^{+/+}$ mice vs. LT-HSCs from control HO-1$^{+/+}$ mice. Color key represents gene expression (as *z*-score among row).

E GSEA among GOBP annotations based on differentially regulated genes.

F Comparison of overlapping genes that were differentially expressed in LT-HSCs from young HO-1$^{-/-}$ mice with genes that were differentially expressed in LT-HSCs from bled HO-1$^{+/+}$ mice.

G PCA based on genes differentially expressed in LT-HSCs from both young HO-1$^{-/-}$ and bled HO-1$^{+/+}$ mice.

© 2019 The Authors
