# Supplementary material for: Heme oxygenase‐1 deficiency triggers exhaustion of hematopoietic stem cells
Source: EMBO Rep. 2019 Dec 29;21(2):e47895. doi: 10.15252/embr.201947895 (PMC7001511; doi:10.15252/embr.201947895)
Supplement: Supplementary file 11 — Review Process File [file EMBR-21-e47895-s011.pdf]

EMBO
reports

Heme oxygenase-1 deficiency triggers exhaustion of hematopoietic stem cells

Krzysztof Szade, Monika Zukowska, Agata Szade,Witold Nowak, Izabella Skulimowska, Maciej Ciesla, Karolina Bukowska-Strakova, Gunsagar Singh Gulati, Neli Kachamakova-Trojanowska, Anna Kusienicka, Elisa Einwallner,Jacek Kijowski, Szymon Czauderna, Harald Esterbauer, Vladimir Benes, Irving Weissman, Jozef Dulak, Alicja Jozkowicz

Review timeline:

Submission date:	8 February 2019
Editorial Decision:	11 February 2019
Revision received:	31 August 2019
Editorial Decision:	11 October 2019
Revision received:	14 November 2019
Accepted:	22 November 2019

Editor: Achim Breiling

Transaction Report: This manuscript was transferred to *EMBO reports* following peer review at *The EMBO Journal.*

(Note: With the exception of the correction of typographical or spelling errors that could be a source of ambiguity, letters and reports are not edited. The original formatting of letters and referee reports may not be reflected in this compilation.)

1st Editorial Decision 11 February 2019

Thank you for the transfer of your research manuscript to EMBO reports. I now went through your paper and the referee reports from The EMBO Journal.

All referees acknowledge the potential interest of the findings. Nevertheless, all three referees have raised a number of concerns and suggestions to improve the manuscript, or to strengthen the data and the conclusions drawn. As the reports are below, I will not detail them here. Looking at the reports, we feel that a significantly revised manuscript could be suitable for publication at EMBO reports, provided the major referee concerns are addressed (as detailed below).

As EMBO reports emphasizes novel functional over detailed mechanistic insight, we will not require to address points regarding more refined mechanistic details. However, if you have such data, we would of course welcome their inclusion in the revised version.

Moreover, we do not think that data using niche cell-specific conditional HO-1 knockout mice would be required, if the alternative experiment suggested by referee #1 is performed (in vitro co-culture assays, LTC-IC). Further, it would be important to test the expression levels of crucial hematopoietic factors on the protein level, and to include rescue experiments (second point of referee #1), and to analyse the influecne of anemia and increased cycling (referee #3). Please also address als the points regarding experimental shortcommings, lacking information, errors, and missing controls. Finally, please have the manuscript proofread by a native speaker (see minor point 6 by referee #3).

Given the constructive referee comments, we would like to invite you to revise your manuscript with the understanding that all referee concerns must be fully addressed in the revised manuscript

(as detailed above) and in a complete point-by-point response. Acceptance of your manuscript will depend on a positive outcome of a second round of review (using the same referees that have assessed the study at The EMBO Journal). It is EMBO reports policy to allow a single round of revision only and acceptance or rejection of the manuscript will therefore depend on the completeness of your responses included in the next, final version of the manuscript.

Revised manuscripts should be submitted within three months of a request for revision; they will otherwise be treated as new submissions. Please contact us if a 3-months time frame is not sufficient for the revisions so that we can discuss the revisions further.

Please refer to our guidelines for preparing your revised manuscript for EMBO reports: http://embor.embopress.org/authorguide#manuscriptpreparation

Supplementary/additional data: The Expanded View format, which will be displayed in the main HTML of the paper in a collapsible format, has replaced the Supplementary information. You can submit up to 5 images as Expanded View. Please follow the nomenclature Figure EV1, Figure EV2 etc. The figure legend for these should be included in the main manuscript document file in a section called Expanded View Figure Legends after the main Figure Legends section. Additional Supplementary material should be supplied as a single pdf labeled Appendix. The Appendix includes a table of content on the first page, all figures and their legends. Please follow the nomenclature Appendix Figure Sx throughout the text and also label the figures according to this nomenclature. For more details please refer to our guide to authors.

Important: All materials and methods should be included in the main manuscript file.

Regarding data quantification and statistics, can you please specify the number "n" for how many experiments were performed, the bars and error bars (e.g. SEM, SD) and the test used to calculate p-values in the respective figure legends? This information must be provided in the figure legends. Please provide statistical testing where applicable. See: http://embor.embopress.org/authorguide#statisticalanalysis

Please also follow our guidelines for the use of living organisms, and the respective reporting guidelines: http://embor.embopress.org/authorguide#livingorganisms

We now strongly encourage the publication of original source data with the aim of making primary data more accessible and transparent to the reader. The source data will be published in a separate source data file online along with the accepted manuscript and will be linked to the relevant figure. If you would like to use this opportunity, please submit the source data (for example scans of entire gels or blots, data points of graphs in an excel sheet, additional images, etc.) of your key experiments together with the revised manuscript. Please include size markers for scans of entire gels, label the scans with figure and panel number, and send one PDF file per figure.

When submitting your revised manuscript, we will require:

- a complete author checklist, which you can download from our author guidelines (http://embor.embopress.org/authorguide#revision). Please insert page numbers in the checklist to indicate where the requested information can be found.
- a letter detailing your responses to the referee comments in Word format (.doc)
- a Microsoft Word file (.doc) of the revised manuscript text
- editable TIFF or EPS-formatted single figure files in high resolution (for main figures and EV figures)

I look forward to seeing a revised version of your manuscript when it is ready. Please let me know if you have questions or comments regarding the revision.

REFEREE REPORTS

Referee #1:

In the current study, Szade et al. describe a role for heme oxygenase 1 (HO-1) expressed by niche cells in the maintenance of hematopoietic stem cells (HSCs) in the bone marrow. The authors found that young mice lacking HO-1 have an expanded pool of activated HSC, and that HO-1-deficient HSCs show signs of premature aging. HO-1 is expressed by endothelial cells and mesenchymal stromal cells in the bone marrow niche, and appears to affect HSC function predominantly in a cell-extrinsic fashion. In addition, HO-1 expression by niche cells decreases with age, and the premature aging phenotype of HSCs in the HO-1-deficient niche could be reversed by transplantation of HO-1-deficient HSCs to wildtype animals. The authors therefore suggest that modulation of HO-1 activity in the bone marrow niche may be used to restore the impaired function of aged HSCs.

Overall, the study is elegantly performed and addresses an important question in the field, i.e. how important are cell-extrinsic versus cell-intrinsic factors in HSC aging? However, while the authors claim that HO-1 is an importance factor in niche cells regulating HSC function and that decreased HO-1 expression by niche cells plays a role in HSC aging, the study lacks solid data to support these claims.

Major comments:

- The key claim of the paper is that HO-1 is important for HSC maintenance through its function in bone marrow niche cells including endothelial cells and cxcl12-abundant reticular cells (CAR cells). However, all experiments are performed using global HO-1 knockout mice. While the transplant experiments nicely show that HO-1 likely affects HSCs in a cell-extrinsic manner, the recipient mice lack HO-1 in all cells, not just the proposed niche cells. While HO-1 may not be expressed by many cell types at steady-state, the recipient mice are lethally irradiated, a stress state that may increase HO-1 expression throughout the body. This is not investigated or discussed. Therefore, in the absence of niche cell-specific conditional HO-1 knockout mice, it is difficult to conclude how much of the observed phenotype is through niche effects rather than systemic changes induced by HO-1 loss. An alternative way to address this would be through in vitro co-culture assays of specific niche populations (ECs or CAR cells) from HO-1 wildtype or deficient animals with wildtype HSCs (for example LTC-IC assays) to show HSC malfunction in a setting where HO-1 is missing specifically in niche cells. Even then the systemic effects that may be occurring in the global KO animals needs to be discussed.

- The results described in Figure 2 are interesting but highly descriptive. Validation of some of the findings on a protein level would support the findings (changes in SDF-1 or SCF expression in niche cells for example). Inclusion of rescue experiments would significantly strengthen the author's claim that HO-1 deletion affects HSCs via its effects in niche cells: several strategies to increase SDF-1 expression in the niche have been described (treatment of mice with Noggin or PTH for example; see Khurana et al. Stem Cells, 2014 or Jung et al. Bone, 2006), which would be a feasible approach. Does loss of HO-1 affect the number of ECs and CAR cells in the BM of young mice? HO-1 is an enzyme that catalyzes the degradation of heme producing biliverdin, ferrous iron, and carbon monoxide. Do the RNAseq data provide any mechanistic insight into how lack of this enzyme possibly affects niche cell function?

- Figure 3, panel b: What are the effects of HO-1 loss on more committed progenitor populations and mature cells? Given the role of different niche populations in the support of HSCs versus more committed progenitors this would be of interest. Panels l-m: The GCSFR data seem a bit out of place. The authors describe a decrease of GCSFR+ HSCs with loss of HO1 or aging, but since this HSC subpopulation has not been described in literature or is not further characterized in the current study, the added value of these analyses is unclear.

- Figure 5: The authors describe an altered pyruvate metabolism in HO-1-deficient LT-HSCs, associated with lower ATP levels. It is however unclear whether the authors believe this is a cause or consequence of HSC malfunction? To show the relevance of the gene expression changes, the authors should also assess glucose metabolism in a functional manner by measuring mitochondrial versus glycolytic activity (for example by Seahorse assay), glucose uptake (for example using the fluorescent glucose analog 2-NBDG) and/or mitochondrial parameters (such as mitochondrial mass, mitochondrial membrane potential).

- Figure 7: Given the focus on pyruvate metabolism in Figure 5, it would be of interest to know whether any genes related to pyruvate metabolism (or more generally to glucose metabolism) were still different between HO-1 WT and KO LT-HSCs after 2 transplantations in a WT host.

- Discussion: The discussion is rather long and a succession of observations rather than a comprehensive text, and would benefit from more structure.
- Discussion: Wysoczynski et al (ref 49) showed that BM stroma of HO-1-/- mice produces less SDF1a and is less capable of supporting HSPC adhesion, this should be acknowledged.

Minor comments:
- How does irradiation affect HO-1 levels in niche cells?
- Figure 2, panels K and L: order of KO and WT should be reverted (as in panel E and F) for clarity. What about Sdf-1 in CAR cells? Scf is shown (but named Kitl?). Some consistency between EC and CAR would help readability. Panel K shows genes related to skeletal biology and bone/cartilage development, but legend says hematopoietic factors.
- Figure 3: Panel a: ST-HSC II population is described but not analyzed anywhere in the paper.
- Figure 6: The authors used HO-1fl/fl;LysM-Cre mice to exclude a role for HO-1 in macrophages. Did the authors check adequate knockout of HO-1 in these mice?
- Throughout the paper, chimerism is described on the Y-axis as "% of GFP+ cells". This should be "% GFP+ cells" or "GFP+ cells (% of total)" For example in Fig. 3N, Fig. 6D, Fig. 7B, C and D.
- Methods, page 43, paragraph 3: Lethal irradiation is said to be 900 cGy, but in the figures is indicated as 9.5Gy

----
Referee #2:

Szade et al present a well-rounded examination of the role that heme oxygenase plays in affecting the potential of HSCs. This niche factor was demonstrated to affect the HSCs' cycling status and aspects of functional potential, in a non-cell autonomous fashion. The authors present a comprehensive analysis of how the niche factor affects the HSCs by demonstrating WT HSCs transplants into HO-1-/- mice recapitulate the phenotype, while HO-1-/- HSC have no significant phenotypes when transplanted into WT recipients. The authors also provide insight into the sub-population of niche cells that may contribute to some age-associated phenotypes. The findings that levels of this niche factor affect the function of HSCs (driving phenotypes that resemble aging) is relevant and important to aging, stem cell, and hematology fields.

Minor Concerns:
The age groups of the young mice (1.5 months) is a bit young for examining the young adult HSC compartment, as long as the comparisons between the ages of WT and HO-1-/- include a similar spectrum of ages: this should be clarified as there are differences seen between the 1 and 3 month old mice (evidenced in figure 3L). 12 month old mice should be classified as mid-aged.

The authors examine only gH2ax staining for measuring DNA damage and stress, but gH2ax staining can be influenced by cell cycle status of the cells. Given that the authors do see differences in the cycling of the HSC compartment, they should include an additional marker of DNA damage- pKAP1, 53BP1, or comet assays to attribute the increased gH2ax to DNA damage / stress. Also, an induced damage control (IR) in the yH2AX intensity plots would be valuable.

The authors demonstrate exposure to HO-1-/- niche environment impairs the overall reconstitution potential of the HO-1+/+ HSCs. Does exposure to decreased HO-1 levels also recapitulate the lineage bias seen in the aged HSC compartment?

In both the EC's and CAR HO-1-/- transcriptomes there is striking PC2 variation- could the authors mention what is driving this/

Fig 3N- double check GFP of recipient

Referee #3:

The manuscript by Kryzstof Szade et al titled Heme oxygenase-1 deficiency affects bone marrow niche and triggers premature exhaustion of hematopoietic stem cells examines the cell extrinsic role of heme oxygenase-1 (HO-1) in maintaining hematopoietic stem cells (HSCs). The authors report that HO-1 is expressed by endothelial cells (ECs) and Cxcl12-abundant reticular cells (CARs) within the bone marrow (BM) niche, and the expression of HO-1 decreases within these cell populations during aging. Using HO-1-deficient mice, the authors demonstrate that HO-1 supports HSC function in a non-cell autonomous manner. Some of the changes in HSCs isolated from HO-1-deficient mice resemble changes that occur during HSC aging. The authors thus claim that HO-1 expression by ECs and CARs is required to prevent premature HSC aging. Although the data provide potentially interesting insight into the regulation of key HSC niche cell types, there are insufficient data to support all of the claims being made. Additional experiments and more precise writing could significantly strengthen this manuscript.

Major Comments:

1. HO-1-deficient mice develop anemia. Can the authors rule out that changes in the HSC compartment do not occur as a consequence of the anemia? For example, does increased HSC cycling occur to produce more erythroid cells in order to compensate for the anemia?

2. A major conclusion of the study is that HSC maintenance requires HO-1 expression by ECs and/or CARs. It is surprising then that the authors only used the HO-1 conditional knockout mice to delete HO-1 in macrophages, but not in ECs and/or CARs. If the effects of HO-1 deficiency on HSCs are indeed mediated by ECs and CARs, then the phenotype should be recapitulated with conditional deletion of HO-1 in one or both of those populations.

3. The authors claim that wild-type HSCs transplanted into HO-1-deficient mice exhibit reduced long-term reconstituting activity and don't reconstitute secondary recipients (Fig. 6). However, HO-1-deficient ECs and CARs express reduced levels of Sdf1 (Fig. 2), which is a key factor required for HSC homing to the BM. Are these results explained by reduced homing/retention of HSCs in the BM after transplantation? Also, do HO-1 deficient mice have elevated numbers of HSCs in the spleen and peripheral blood?

4. The authors claim that HSCs in HO-1-defcient mice exhibit premature aging. This is based on the HO-1-defcient HSCs exhibiting increased cycling, increased DNA damage, reduced reconstituting activity and some changes in gene expression. Do these changes really reflect premature aging? Perhaps the changes in DNA damage, reconstituting activity and gene expression mostly reflect the increased cycling of HSCs in HO-1 deficient mice? The authors should compare HSCs from HO-1-deficient mice to wild-type HSCs that have been driven into cycle.

5. The most significant age-related change in HSCs is myeloid skewing (increased myeloid and reduced lymphoid differentiation). Do the authors observe myeloid biased differentiation from HSCs isolated from HO-1-deficient mice? If not, the authors should reconsider their claim of premature aging.

6. The "old" animals used in this study are typically 11-12 months old, and in some cases 18 months old. This is significantly younger than animals used in most HSC aging studies. In order to claim premature aging, the authors should use sufficiently old (>20-month-old) mice.

7. Many of the differences within HSCs observed in HO-1-deficient mice disappear in 11-12-month-old mice (Fig. 3, 4). However, in Fig. 7 the authors claim that prolonged residence in a wild-type environment rescues transcriptional changes in HO-1-deficient HSCs. These experiments in Fig. 7 occur 48 weeks after transplant. How can the authors distinguish whether the rescue occurs because of the environment or because sufficient time has elapsed to allow for normalization of the transcriptional changes?

8. The authors claim to identify GCSFR expression as a new marker of aged HSCs. No validation of this marker is provided, and its overall relevance to this manuscript is questionable and distracting.

9. The metabolic data in Figure 5 do not provide significant mechanistic insight. If these data can't

be expanded, I would suggest removing them entirely from the manuscript.

10. The authors need to state their claims more carefully. For example, in the Abstract the authors state that "HSCs from young HO-1-/- animals lose... regenerative potential". This statement is misleading. Consistent with the authors' claims, the regenerative capacity of HO-1-deficient HSCs is significantly reduced. There is an important difference between reduced regenerative capacity and lost regenerative potential.

Minor Comments:

1. What do the individual symbols represent in Figure 1H? The figure legend indicates that n for this experiment is 10-11, and yet there are only 3-4 data points.

2. What do the red dots represent in Figure 3E?

3. Are the time points in Figure 3N shifted relative to the labels on the x-axis? Were samples taken at 4, 8, 12, and 16 weeks?

4. The Methods section is incomplete.
a. The gamma H2Ax staining description is missing.
b. The precise setup of transplantation experiments is difficult to interpret (both from the Methods section and schematics in figures). Are these always competitive transplants? Also, the irradiation dose listed in the Methods section differs from what is shown in the schematics.

5. What is the reason for using 18-month-old animals for RNA seq experiments and 11-12 months old animals for other experiments assessing aging phenotype?

6. The manuscript has many typos and inconsistent labeling in Figures (LKS/KLS; days after transplant/weeks after transplant), which makes the text unclear and difficult to follow.

7. Negative controls for HO-1 staining should be included (i.e. cells and tissue from HO-1-deficient mice).

1st Revision - authors' response 31 August 2019

Dear Reviewers
Dear Editorial Board

We are grateful for in-depth and precise reviews. The reviews showed us where our reasoning was unclear, what additional experiments would help to understand the observed phenotype and that the interpretation of our data could be significantly improved. Simultaneously, we thank the Reviewers for acknowledging the high quality of our experiments. We would like to respond to the concerns raised by Reviewers in general, as well as in details with point-by-point comments.
We understand that the main critics raised by the Reviewers concern ascribing the altered phenotype of HSCs in global HO-1 knock-out mice to the bone marrow niche. **We do agree with the Reviewers and the Editorial Board that we did not have direct proof that niche cells are the only cause for HSC impairment in global HO-1 knock-out mice and we could not exclude the potential systemic factors.**
To address this point, **we performed additional experiments suggested by the Reviewers, that showed that *in-vitro* co-cultures of HSCs with BM-derived mesenchymal cells from HO-1$^{-/-}$ mice recapitulate altered function of HSCs while systemic anemia did not induce LT-HSC phenotype observed in HO-1$^{-/-}$ mice**. Although these results (described in details below) might indicate that niche mediates the LT-HSC phenotype in HO-1$^{-/-}$, **we do not propose interpretation that suggests the lone niche role**. Our data show, we believe, that differences between WT and HO-1 KO LT-HSCs are governed extrinsically, but we do not exclude other potential systemic factors. According to the Reviewers remarks we changed also the title of the manuscript, now: *"Heme oxygenase-1 deficiency affects bone marrow niche and triggers exhaustion of hematopoietic stem cells in cell-extrinsic manner."*
We believe that additional experiments, more precise writing, and restatement of our claims made our article stronger, and we provide conclusions fully supported by the data.

Point-by-point response

Referee #1:

Synopsis:
In the current study, Szade et al. describe a role for heme oxygenase 1 (HO-1) expressed by niche cells in the maintenance of hematopoietic stem cells (HSCs) in the bone marrow. The authors found that young mice lacking HO-1 have an expanded pool of activated HSC, and that HO-1-deficient HSCs show signs of premature aging. HO-1 is expressed by endothelial cells and mesenchymal stromal cells in the bone marrow niche, and appears to affect HSC function predominantly in a cell-extrinsic fashion. In addition, HO-1 expression by niche cells decreases with age, and the premature aging phenotype of HSCs in the HO-1-deficient niche could be reversed by transplantation of HO-1-deficient HSCs to wildtype animals. The authors therefore suggest that modulation of HO-1 activity in the bone marrow niche may be used to restore the impaired function of aged HSCs.

Overall, the study is elegantly performed and addresses an important question in the field, i.e. how important are cell-extrinsic versus cell-intrinsic factors in HSC aging? However, while the authors claim that HO-1 is an importance factor in niche cells regulating HSC function and that decreased HO-1 expression by niche cells plays a role in HSC aging, the study lacks solid data to support these claims.

Major comments:
- The key claim of the paper is that HO-1 is important for HSC maintenance through its function in bone marrow niche cells including endothelial cells and cxcl12-abundant reticular cells (CAR cells). However, all experiments are performed using global HO-1 knockout mice. While the transplant experiments nicely show that HO-1 likely affects HSCs in a cell-extrinsic manner, the recipient mice lack HO-1 in all cells, not just the proposed niche cells.
While HO-1 may not be expressed by many cell types at steady-state, the recipient mice are lethally irradiated, a stress state that may increase HO-1 expression throughout the body. This is not investigated or discussed.
Therefore, in the absence of niche cell-specific conditional HO-1 knockout mice, it is difficult to conclude how much of the observed phenotype is through niche effects rather than systemic changes induced by HO-1 loss. An alternative way to address this would be through in vitro co-culture assays of specific niche populations (ECs or CAR cells) from HO-1 wildtype or deficient animals with wildtype HSCs (for example LTC-IC assays) to show HSC malfunction in a setting where HO-1 is missing specifically in niche cells. Even then the systemic effects that may be occurring in the global KO animals needs to be discussed.

As mentioned in our general response, we fully agree with the Reviewer, that the observed LT-HSC phenotype in HO-1$^{-/-}$ mice cannot be interpreted as lone niche-dependent effect. We agree that conditional deletion of HO-1 in mice would provide more precise answer, however we estimate it would take around 1.5 year to complete these experiments (crossing, expanding the colony, inducing deletion of HO-1, and transplantation of HSC), and still it might be difficult to definitely distinguish local and systemic cell-type specific effects. Therefore, with acceptance of the Editorial Board, **we performed the *in-vitro* experiment proposed by the Reviewer as an alternative approach to replace mice models**.
We established *in-vitro* mesenchymal stromal cells (MSC) cultures from HO-1$^{+/+}$ and HO-1$^{-/-}$ mice and performed modified LTC-IC assay. We prepared confluent 96-well plates and sorted single LSK CD150^{+}CD48^{-} HSCs from HO-1$^{+/+}$ GFP^{+} mice per well in 10% FBS DMEM HG medium. After one week we changed medium for serum free StemSpan differentiation medium supplemented with SCF, TPO, EPO, IL3 and BIT9500 (BSA, insulin, iron saturated transferrin). Then we monitored colony formation and after 14 days we analyzed each of the colony by flow cytometry to evaluate cell phenotype. Such protocol based on single GFP^{+} HSC allows us to monitor early appearance of the colonies and efficiently track the fate of single HSC.
The results of this experiment constitute the new additional Figure 8 in the manuscript (pasted also below).

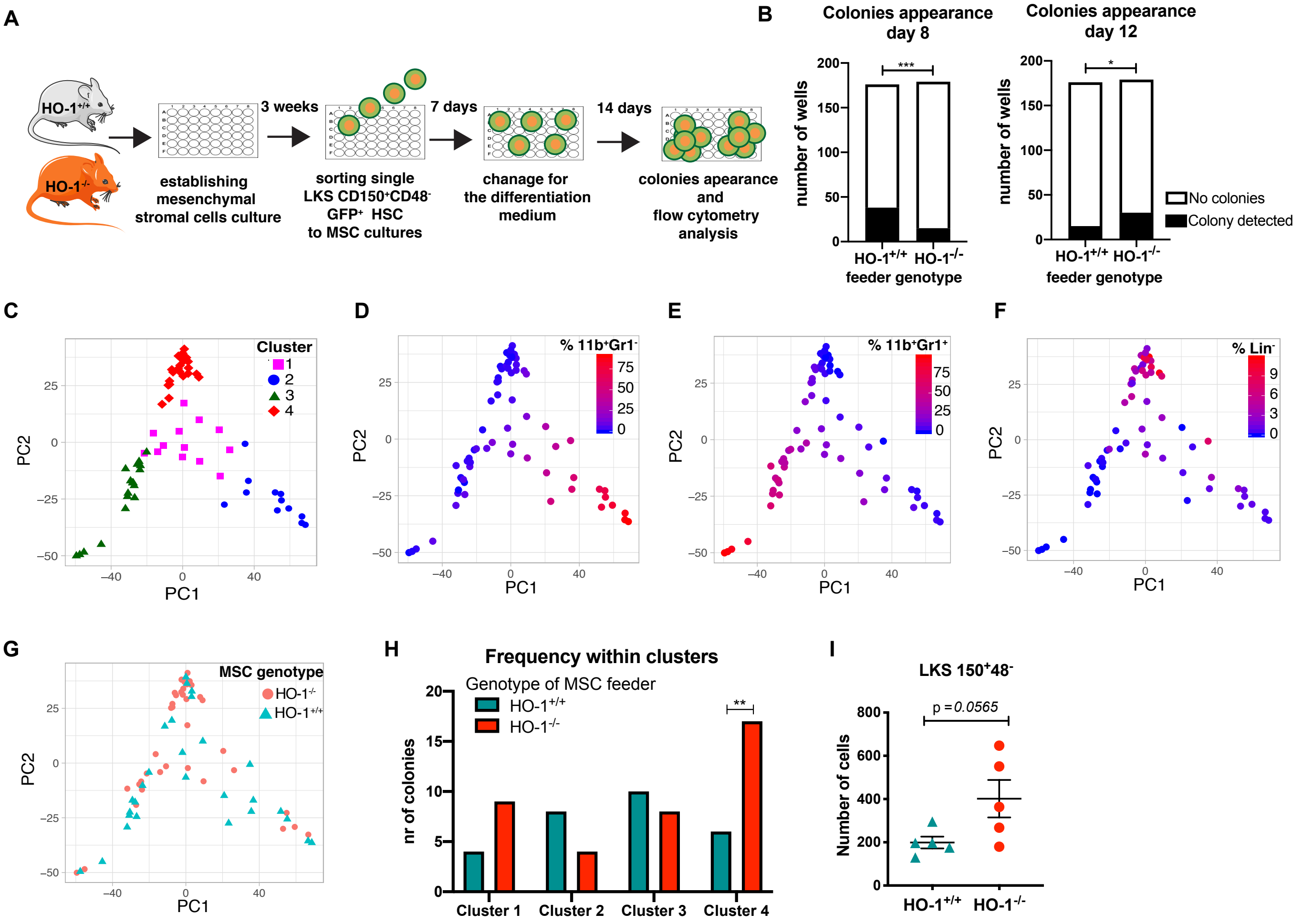

New **Figure 8** in the manuscript – colony formation assay on MSCs from HO-1$^{+/+}$ or HO-1$^{-/-}$ mice.

We observed that colonies from HSCs co-cultured with HO-1$^{-/-}$ MSCs appeared later in the culture than ones derived from HSCs co-cultured with HO-1$^{+/+}$ MSCs (new Fig. 8B), however the total size and efficiency of colony formation did not differ (new Fig. EV8AB).

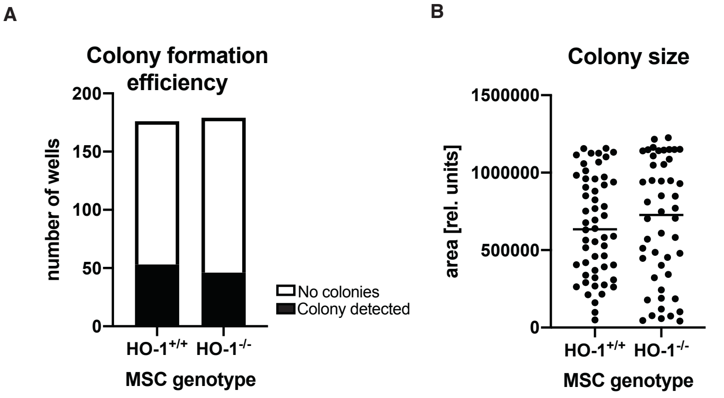

New **Figure EV8AB** in the manuscript – efficiency of colony formation and size of the colonies.

Then we analyzed the phenotype of the colonies by flow cytometry. We gated the main observed phenotypes based on CD11b, Gr1, B220, CD3 and performed PCA analysis. The majority of the observed cells were of myeloid CD11b^{+} origin, but the PCA analysis and unsupervised clustering allowed us to distinguish 4 clusters (new Fig. 8C). Clusters were mainly defined by CD11b^{+}Gr1^{-}, CD11b^{+}Gr1^{+}, and cells that lack expression of analyzed markers (called "lineage^{-}", however they may represent lineages not analyzed here) (new Fig. 8DEF). There were only few B220^{+} cells detected (new Fig. EV8C in the manuscript).

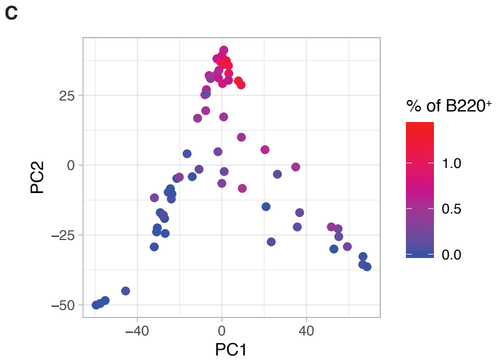

New **Figure EV8C** in the manuscript – frequency of B220^{+} cells among colonies.

We did observe that colonies co-cultured with MSCs from HO-1$^{-/-}$ mice were significantly enriched in cluster 4. While the *in-vitro* assays are only simplified model of physiological niche and we did not analyze all blood phenotypes, we believe that this experiment showed the niche-dependent HO-1 effect on kinetic of growth and differentiation of HSC-derived colonies. Next, when 10k LSKs were seeded on MSCs from HO-1$^{-/-}$ mice, after one week we detected more cells with HSC phenotype (LSK 150^{+}CD48^{-}) (new Fig. 8I). We are aware that we study here only mesenchymal fraction of the BM niche, and the endothelial component is missing, but in our hands *in-vitro* culture of primary non-transformed BM-derived ECs does not work well.
We performed also additional experiment (please see response to Reviewer #3, and new Fig. 9 in the manuscript) with bleeding of HO-1$^{+/+}$, to induce anemia and see whether such systemic challenge can elicit LT-HSC phenotype observed in HO-1$^{-/-}$ mice, but we did not observe significant changes among HSCs.
Nevertheless, we fully agree with the Reviewer that even with these experiments we cannot rule out other systemic factors that can contribute to the observed phenotype of LT-HSCs in HO-1$^{+/+}$ mice. Therefore we changed the title of the manuscript and, as suggested, we discussed it in the paper – cited from the discussion:

"*While our functional experiments demonstrate the cell-extrinsic role of HO-1 in regulation of HSC exhaustion, the question remains whether this cell-extrinsic role is mediated by local HSC niche or by systemic factors, eg. linked to iron deficiency and microcytic anemia in HO-1$^{-/-}$ mice. We demonstrated high HO-1 expression in steady state conditions in BM niche, characterized which populations express HO-1, how the expression changes in aging, and demonstrated that lack of HO-1 in niche cells dysregulates production of hematopoietic fractions. Moreover, we showed that co-cultured of HSCs with HO-1$^{-/-}$ stromal cells affects their numbers, kinetic of growth*

and differentiation of the derived colonies (Fig 8). In contrast, serial bleeding does not induce the phenotype of LT-HSC observed in HO-1$^{-/-}$ mice (Fig 9).
Given our findings on role of HO-1 in BM niche, it is likely that lack of HO-1 in the niche cells trigger the phenotype of LT-HSCs in HO-1$^{-/-}$ mice. However, we cannot exclude that other systemic factors intertwine with HO-1 role within the BM niche and together contribute to dysregulation of HSC in HO-1$^{-/-}$ mice."

We fully agree with the Reviewer that irradiation of mice may upregulate HO-1 expression. While we did not check the levels of HO-1 after irradiation, we can indirectly discuss the role of potential induction of HO-1 upon irradiation based on irradiation of HO-1$^{-/-}$ mice. We did not observe any increased morbidity or mortality of HO-1$^{-/-}$ mice upon irradiation. All mice survived the conditioning and transplantation, demonstrating that HO-1$^{-/-}$ mice can be used in standard transplantation protocols as recipients. Furthermore, we did not observe any short-term effects on hematopoietic parameters in HO-1$^{-/-}$ recipients (Fig. 6C) that one may expect when HO-1 dependent protection early after irradiation is crucial. Instead, we observed long-term effects on hematopoiesis, especially during secondary transplantation assay (Fig. 6C,F), what is thought to be classical output to measure HSC function.
We discussed the potential induction of HO-1 in HSCs upon transplantation, as a factor that can potentially affect interpretation of our data, especially when HO-1$^{-/-}$ cells were transplanted. Indeed, this potentially could explain worse reconstitution after HO-1$^{-/-}$ HSC transplantation. However, when we made secondary transplantation of HO-1$^{-/-}$ HSCs, no difference in reconstitution potential was visible, what indicates that their worse reconstitution in primary transplant does not depend on intrinsic HO-1 expression. As suggested by the Reviewer this is included in part of the discussion:

"Finally, while HO-1 is not expressed in HSCs in steady-state it might be expressed upon stress conditions [1]. Thus, lack of HO-1 during the transplantation stress may affect their viability. To avoid this equivocally interpretations we did serial transplantion of the same number of donor-derived cells from primary recipients into the secondary recipients. By this way we provided the same conditions for the compared groups. In such experimental settings, the secondary HO-1$^{+/+}$ niche fully restored the reconstitution potential of HO-1$^{-/-}$ HSCs to equal that of control HO-1$^{+/+}$ HSCs (Figure 7C). This implies that the young HO-1$^{+/+}$ niche can reverse the impaired reconstitution capacity of HO-1$^{-/-}$ HSCs and that the induction of HO-1 in HSCs during transplantation is not limiting factor in the assay."

- The results described in Figure 2 are interesting but highly descriptive. Validation of some of the findings on a protein level would support the findings (changes in SDF-1 or SCF expression in niche cells for example). Inclusion of rescue experiments would significantly strengthen the author's claim that HO-1 deletion affects HSCs via its effects in niche cells: several strategies to increase SDF-1 expression in the niche have been described (treatment of mice with Noggin or PTH for example; see Khurana et al. Stem Cells, 2014 or Jung et al. Bone, 2006), which would be a feasible approach. Does loss of HO-1 affect the number of ECs and CAR cells in the BM of young mice? HO-1 is an enzyme that catalyzes the degradation of heme producing biliverdin, ferrous iron, and

carbon monoxide. Do the RNAseq data provide any mechanistic insight into how lack of this enzyme possibly affects niche cell function?

We thank Reviewer for the suggestions. As we performed RNA-seq analysis in defined minor niche population, these changes may be not recapitulated when whole BM protein is analyzed. Therefore, we established intracellular flow cytometry analysis of selected factors and these results are presented as new Fig. 3 in the manuscript. We confirmed that CARs isolated from HO-1$^{-/-}$ mice produce less SDF-1α, while ECs from HO-1$^{-/-}$ mice produce less Tgfβ1 (we analyzed LAP peptide, which is a product of Tgfβ1 gene and makes inactive complex with Tgfβ1 protein).

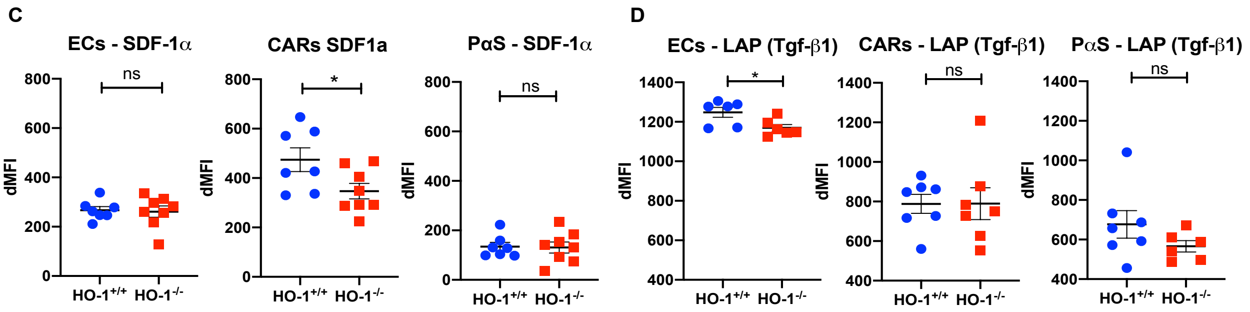

New **Figure 3** in the manuscript, panel C,D – intracellular production of SDF-1α and LAP (TGF-β1) in tested niche populations.

Moreover, we analyzed membrane-bond surface SCF (we were not able to reliably detect intracellular SCF). We observed minor populations that were SCF$^+$ among ECs and CARs, with tendency (p = 0,068) to lower frequency among ECs in HO-1$^{-/-}$ mice, and no differences among CARs (we did not detect the SCF$^+$ PαSs).

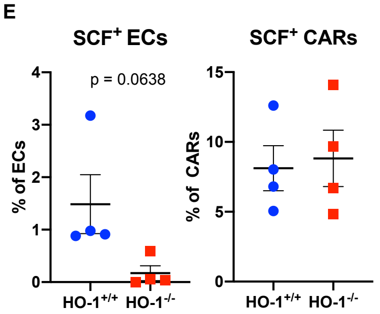

New **Figure 3** in the manuscript, panel E – frequency of surface SCF$^+$ ECs and CARs

Our results showed that the altered production of hematopoietic factors in HO-1$^{-/-}$ mice is population specific. This is important as Morrison group showed that deletion of crucial hematopoietic factors in different population exert different effect on LT-HSCs [2–4].
As suggested by the Reviewer, we performed an attempt to reverse the decreased SDF-1α levels in the HO-1$^{-/-}$ niche. We chose Noggin and administered 500 μg/kg every second day for 2 weeks and 2 days after last dose we checked Sdf1α mRNA levels and evaluated the phenotype of the LT-HSC. However, in our hands, we did not observe increased Sdf1α expression after *in-vivo* administration of Noggin (show in the manuscript in new Fig. 8J). Thus, our attempt to rescue the SDF-1α levels was not successful as

expected by the literature data. Accordingly, we did not observe any rescue of the LT-HSC phenotype in HO-$1^{-/-}$ mice (Fig. 8K,M), or any effect on hematological parameters or frequency of HSC and progenitors in HO-$1^{-/-}$ or HO-$1^{+/+}$ mice (not shown).

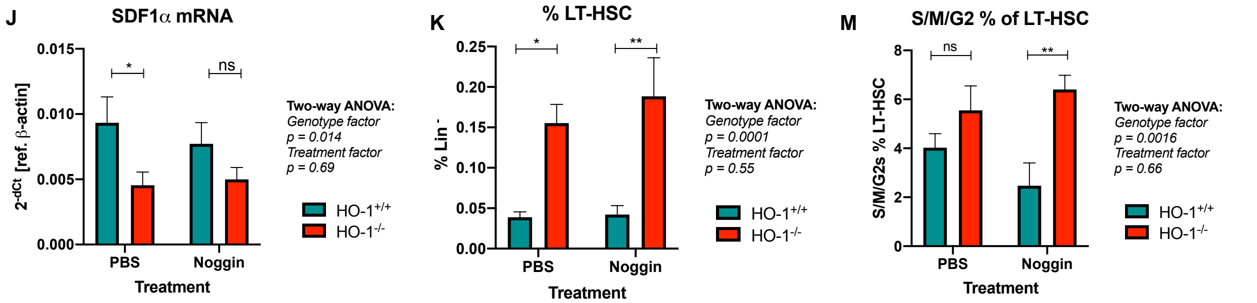

New **Figure 8** in the manuscript, panel J,K,M – lack of Noggin treatment in vivo on Sdf1α levels and LT-HSC phenotype.

According to the mechanistic role of HO-1, we investigated the possible influence of carbon monoxide (CO) as one of the HO-1 product. First, we found that differences in CO levels in the BM of HO-1 WT and KO mice are moderate (Fig. R1A – not included in the manuscript). This is possible due to activity of constitutive HO-2 that also produces CO. Broxmeyer group showed that isolation and incubation of HSCs in low oxygen can increase HSC quiescence upon transplantation [5]. Therefore, we hypothesized that incubation with CO, which blocks oxidative phosphorylation, may have similar effect. However, incubation of HSC for 36 hours with 250 ppm CO and subsequent transplantation did not show any influence of CO on HSCs, (Fig. R1A). Therefore, our data do not suggest that CO can mediate the observed phenotype of LT-HSC in the HO-$1^{-/-}$ mice.

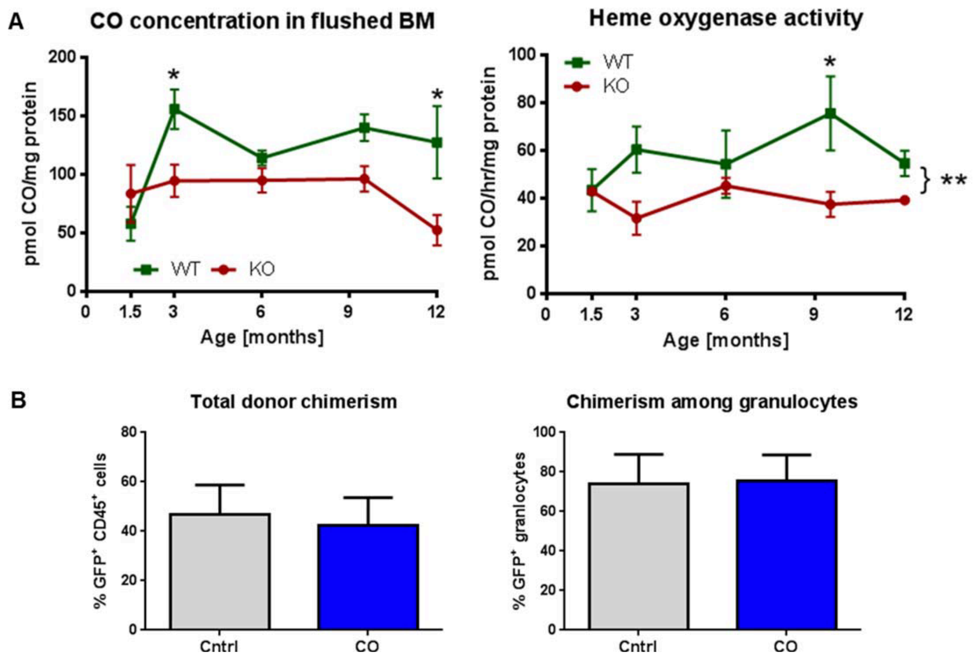

Figure R1 – not included in the manuscript. Assessing the role of CO on HSC. (A) CO measured in total BM by gas chromatography (B) Effect of incubation of HSC for 36h with 250ppm CO and subsequent transplantation.

Indeed, our RNA-seq data from young WT and HO-1 KO HSCs show possibly altered iron metabolism. First, there are significant differences in expression of genes associated with iron uptake and storage, *e.g.*, Tfrc transferrin receptor, Steap3 metalloreductase, or Fth1 and Ftl1 ferritin chains (Fig. R2 – not included in the manuscript). Next, we observed dysregulation of genes involved in iron metabolism/regulated by iron (Fig. R3 – not included in the manuscript). This indicates that intracellular ferrous ions (as one of the HO-1 product) or availability of iron recycled from heme by macrophages (in a process mediated by HO-1) may be the mechanistic mediator. However, we checked the amount of ferrous ions (using FeRhoNox™-1 fluorescent probe) specifically in HSC and other progenitor populations by flow cytometry and did not observe any differences. Therefore, we could not definitively conclude about the possible altered iron metabolism as mechanism explaining the phenotype of HO-1$^{-/-}$ LT-HSCs.

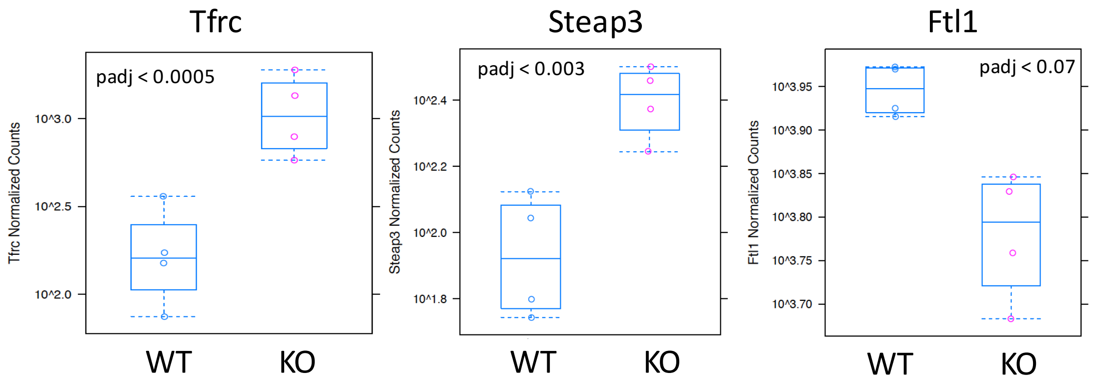

Figure R2 – not included in the manuscript. Expression of Tfrc transferrin receptor, Steap3 metalloreductase and Ftl1 ferritin chains in young WT and HO-1KO HSC, RNA-seq data.

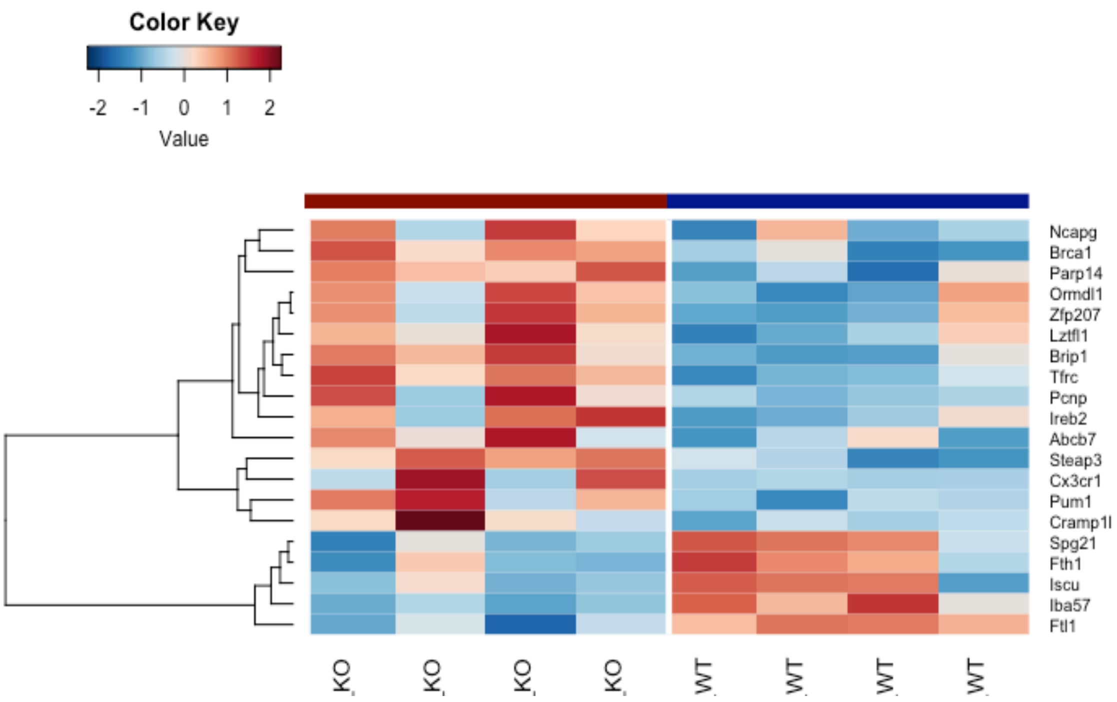

Figure R3 – not included in the manuscript. Expression of selected genes in WT and HO-1 KO HSC involved in iron metabolism/regulated by iron. RNA-seq data.

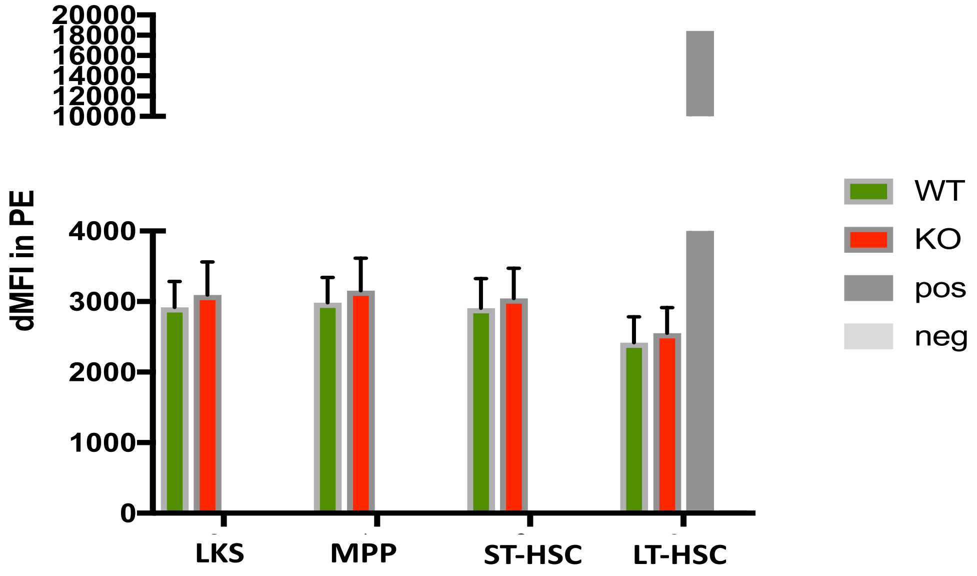

Figure R4 – not included in the manuscript – Levels of labile $Fe2^{+}$ in progenitors and HSC. Positive control – incubation with Mohr's salt.

- Figure 3, panel b: What are the effects of HO-1 loss on more committed progenitor populations and mature cells? Given the role of different niche populations in the support of HSCs versus more committed progenitors this would be of interest. Panels l-m: The GCSFR data seem a bit out of place. The authors describe a decrease of GCSFR+ HSCs with loss of HO1 or aging, but since this HSC subpopulation has not been described in literature or is not further characterized in the current study, the added value of these analyses is unclear.

We analyzed MEP ($Lin^{-}Kit^{+}Sca1^{-}CD150^{+}CD48^{mid}$), GMP ($Lin^{-}Kit^{+}Sca1^{-}CD150^{-}CD48^{+}CD34^{+}$) and EP ($Lin^{-}Kit^{+}Sca1^{-}CD150^{-}CD48^{low}$) in BM of HO-$1^{-/-}$ mice and included these results as new Fig. EV4B.

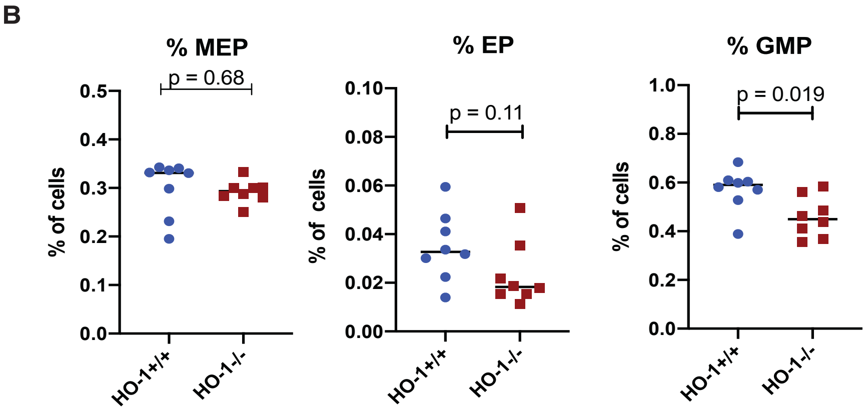

New **Figure EV4B** – included in the manuscript. Frequency of committed progenitors in HO-$1^{-/-}$ mice.

GMP are less frequent in HO-$1^{-/-}$ mice, while MEP and EP are not significantly affected. The more detailed analysis of specific myelocyte stages are included in our previous work [6]. The HO-$1^{-/-}$ mice have more granulocytes and monocytes in peripheral blood as also shown previously (Fig. R5 – not included in the manuscript).

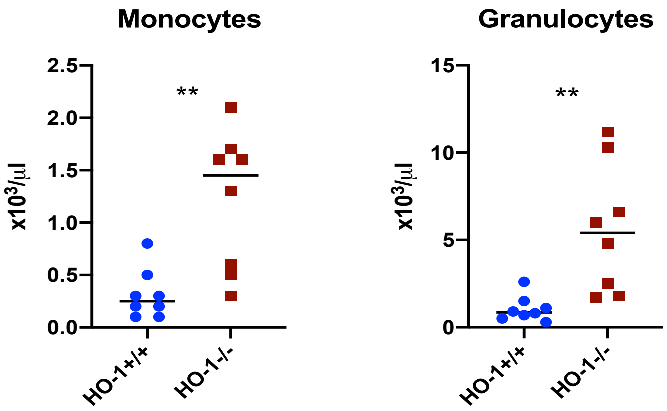

Figure R5 – not included in the manuscript – granulocyte and monocyte counts in peripheral blood of HO-$1^{-/-}$ mice.

We thank for the remark and agree with the comment about GCSFR data – accordingly, we decided to remove these results.

- Figure 5: The authors describe an altered pyruvate metabolism in HO-1-deficient LT-HSCs, associated with lower ATP levels. It is however unclear whether the authors believe this is a cause or consequence of HSC malfunction? To show the relevance of the gene expression changes, the authors should also assess glucose metabolism in a functional manner by measuring mitochondrial versus glycolytic activity (for example by Seahorse assay), glucose uptake (for example using the fluorescent glucose analog 2-NBDG) and/or mitochondrial parameters (such as mitochondrial mass, mitochondrial membrane potential).

We agree that the proposed methods would reveal the potential role of disturbed metabolism in our analyses. However, according to the Editorial Board that may be beyond the scope of the manuscript.

- Figure 7: Given the focus on pyruvate metabolism in Figure 5, it would be of interest to know whether any genes related to pyruvate metabolism (or more generally to glucose metabolism) were still different between HO-1 WT and KO LT-HSCs after 2 transplantations in a WT host.

We checked several genes connected with pyruvate and glucose metabolism and did not detect significant changes among the genes in KO LT-HSC after 2 transplantations (Table R1 – not included in the manuscript). While not statistically significant, the gene that showed the biggest difference was Ldhb.

	baseMean	log2FoldChange	pvalue	padj	gene_name
Ldhb	2854.61475	1.53740115	0.00477027	0.47687019	lactate dehydrogenase B
Gapdh	82.0321333	1.267192469	0.15918485	0.9995844	glyceraldehyde-3-phosphate dehydrogenase
Pdk3	1197.02644	0.816013463	0.35601141	0.9995844	pyruvate dehydrogenase kinase, isoenzyme 3
Hk1	1178.93041	-0.611291234	0.49500674	0.9995844	hexokinase 1
Pdha1	1427.46056	-0.5027232	0.52711022	0.9995844	pyruvate dehydrogenase E1 alpha 1
Eno1b	261.477978	-0.403954618	0.65684661	0.9995844	enolase 1B, retrotransposed
Pdhx	93.20862	-0.3145546	0.687505	0.9995844	pyruvate dehydrogenase complex, component X
Hk2	445.180652	-0.364189463	0.68817225	0.9995844	hexokinase 2
Ldha	12237.9997	-0.1176332	0.73581397	0.9995844	lactate dehydrogenase A
Pdhb	3684.59448	0.17900627	0.76558502	0.9995844	pyruvate dehydrogenase (lipoamide) beta
Pkm	7215.63558	0.074794313	0.87913366	0.9995844	pyruvate kinase, muscle
Eno1	142.910444	0.122171855	0.88867066	0.9995844	enolase 1, alpha non-neuron
Pdk1	1508.70808	-0.059668568	0.94400888	0.9995844	pyruvate dehydrogenase kinase, isoenzyme 1
Pdk2	72.161571	-0.094962012	NA	NA	pyruvate dehydrogenase kinase, isoenzyme 2
Eno3	403.806334	0.557015769	NA	NA	enolase 3, beta muscle
Hk3	574.171884	-0.979270653	NA	NA	hexokinase 3

Table 1R – not included in the manuscript – differential expression of selected genes included in pyruvate and glucose metabolism in HO-$1^{-/-}$ and HO-$1^{+/+}$ HSCs after 2 transplantations to WT recipient.

- Discussion: The discussion is rather long and a succession of observations rather than a comprehensive text, and would benefit from more structure.

Thank you for the suggestion. We shortened the discussion and removed the repeated parts.

- Discussion: Wysoczynski et al (ref 49) showed that BM stroma of HO-1-/- mice produces less SDF1a and is less capable of supporting HSPC adhesion, this should be acknowledged.

We acknowledged this finding in the discussion. Nevertheless, regarding the HSPC adhesion, the authors analyzed very broad population, and we are not sure how this can be compared to analysis of adhesion of defined HSC:

"Other group revealed that HO-1 inhibition may be a strategy to improve mobilization of HSC [52] and that cultured stromal cells produces less SDF-1α and present impaired ability to support HSPC adhesion [52]. While the conclusions of these papers are in line with our work, here we describe a broader role of HO-1 in strictly defined HSCs."

Minor comments:
- How does irradiation affect HO-1 levels in niche cells?

We discussed this point in response to your previous comment.

- Figure 2, panels K and L: order of KO and WT should be reverted (as in panel E and F) for clarity. What about Sdf-1 in CAR cells? Scf is shown (but named Kitl?). Some consistency between EC and CAR would help readability. Panel K shows genes related to skeletal biology and bone/cartilage development, but legend says hematopoietic factors.

Thank you for the remark – we corrected the Figure 2 according to your comment.

Figure 3: Panel a: ST-HSC II population is described but not analyzed anywhere in the paper.

Using the panel of antigens, we are able to distinguish several progenitor populations, however we were not able to show data from all of them in the main figures. Therefore, we included data regarding ST-HSC II frequency in the new Fig. EV4A.

A

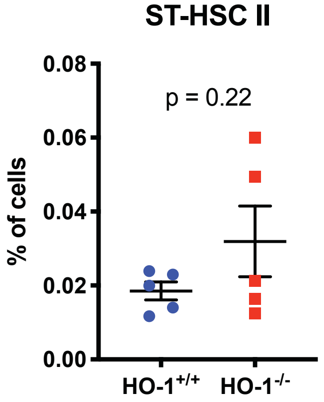

New **Figure EV4A** – included in the manuscript – frequency of ST-HSCII population.

- Figure 6: The authors used HO-1fl/fl;LysM-Cre mice to exclude a role for HO-1 in macrophages. Did the authors check adequate knockout of HO-1 in these mice?

These mice were provided by our collaborators, who characterize the adequate knock-downed HO-1 levels in the paper by Jais et al. Cell 2014, Fig. 3AB. [7]

- Throughout the paper, chimerism is described on the Y-axis as "% of GFP+ cells". This should be "% GFP+ cells" or "GFP+ cells (% of total)" For example in Fig. 3N, Fig. 6D, Fig. 7B, C and D.

Thank you for the comment – we corrected this.

- Methods, page 43, paragraph 3: Lethal irradiation is said to be 900 cGy, but in the figures is indicated as 9.5Gy.

We are sorry for this inconsistency: we used 9 or 9.5 Gy depending on the experiment. We clarified this in the methodology section.

Referee #2:

Szade et al present a well-rounded examination of the role that heme oxygenase plays in affecting the potential of HSCs. This niche factor was demonstrated to affect the HSCs' cycling status and aspects of functional potential, in a non-cell autonomous fashion. The authors present a comprehensive analysis of how the niche factor affects the HSCs by demonstrating WT HSCs transplants into HO-1-/- mice recapitulate the phenotype, while HO-1-/- HSC have no significant phenotypes when transplanted into WT recipients. The authors also provide insight into the sub-population of niche cells that may contribute to some age-associated phenotypes. The findings that levels of this niche factor affect the

function of HSCs (driving phenotypes that resemble aging) is relevant and important to aging, stem cell, and hematology fields.

Minor Concerns:
The age groups of the young mice (1.5 months) is a bit young for examining the young adult HSC compartment, as long as the comparisons between the ages of WT and HO-1-/- include a similar spectrum of ages: this should be clarified as there are differences seen between the 1 and 3 month old mice (evidenced in figure 3L). 12 month old mice should be classified as mid-aged.

Thank you for the comment – all comparisons were done between mice within the same age group – young mice were 1.5-3 month old. We always took mice with matching ages when comparing HO-$1^{-/-}$ and HO-$1^{+/+}$, even within the same group. Indeed, in one analysis regarding GCSFR expression we included 1 month old mice, however according to other Reviewers' comments, these data were removed. We classified the 12 months group as mid-age in the revised article.

The authors examine only gH2ax staining for measuring DNA damage and stress, but gH2ax staining can be influenced by cell cycle status of the cells. Given that the authors do see differences in the cycling of the HSC compartment, they should include an additional marker of DNA damage- pKAP1, 53BP1, or comet assays to attribute the increased gH2ax to DNA damage / stress. Also, an induced damage control (IR) in the yH2AX intensity plots would be valuable.

Thank for the comment, we fully agree with the Reviewer. We performed alkaline comet assay to directly verify DNA damage in HSCs. The analysis confirmed significantly more DNA damage in HO-$1^{-/-}$ HSCs. These data are now included in main manuscript in Fig. 4L,M.

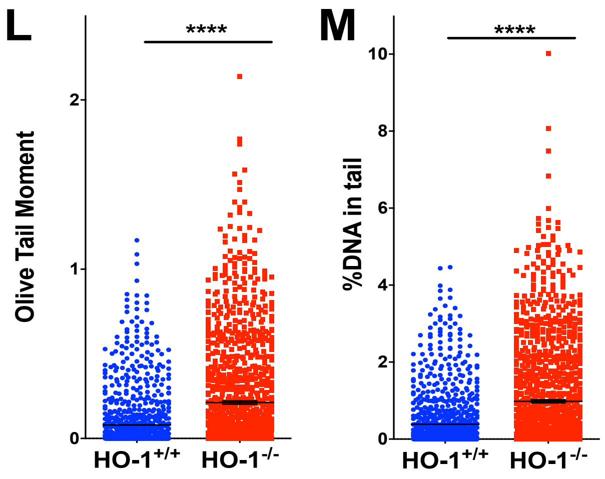

New **Figure 4**, panel L,M – included in the manuscript – comet assay indicated more DNA damage in the HO-$1^{-/-}$ LT-HSC.

We did not present the signal from the induced damage controls (Etoposide 100 μM) on the histograms as they make graph harder to read, but we show below the signal we obtained with induced damage and our protocol (Fig R6).

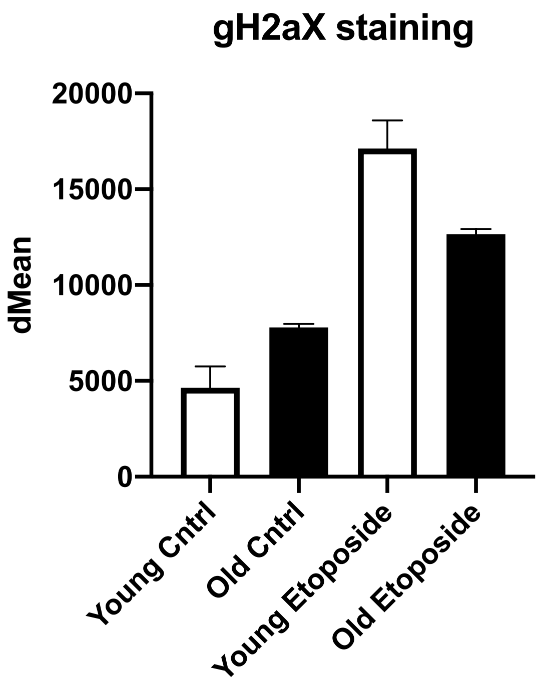

Figure R6 – not presented in the manuscript – gH2ax staining in LKS after etoposide

The authors demonstrate exposure to HO-1-/- niche environment impairs the overall reconstitution potential of the HO-1+/+ HSCs. Does exposure to decreased HO-1 levels also recapitulate the lineage bias seen in the aged HSC compartment?

We did not observe the bias after transplantation to the HO-1 deficient mice, but we generally observed myeloid biased contribution in both transplanted groups (HO-1$^{+/+}$ and HO-1$^{-/-}$) in this experiment. While our endpoint was 32 weeks after transplant, and with the additional secondary transplants, we suppose that HO-1$^{+/+}$ HSCs might already age and acquire myeloid bias during this period.

In both the EC's and CAR HO-1-/- transcriptomes there is striking PC2 variation- could the authors mention what is driving this.

While we checked genes with highest loading to PC2 in both groups we did not find any particular group of genes that are annotated to defined biological process or biology of studied populations. Therefore, we think that this variation represents random noise. This RNA-seq experiment is performed from the low number of sorted cells and requires Smartseq2 based amplification. This method always contribute to increased noise comparing to the RNA-seq done from high number of cells.

Referee #3:

The manuscript by Kryzstof Szade et al titled Heme oxygenase-1 deficiency affects bone marrow niche and triggers premature exhaustion of hematopoietic stem cells examines the cell extrinsic role of heme oxygenase-1 (HO-1) in maintaining hematopoietic stem cells (HSCs). The authors report that HO-1 is expressed by endothelial cells (ECs) and Cxcl12-abundant reticular cells (CARs) within the bone marrow (BM) niche, and the expression of HO-1 decreases within these cell populations during aging. Using HO-1-deficient mice, the authors demonstrate that HO-1 supports HSC function in a non-cell autonomous manner. Some of the changes in HSCs isolated from HO-1-deficient mice resemble changes that occur during HSC aging. The authors thus claim that HO-1 expression by ECs and CARs is required to prevent premature HSC aging. Although the data provide potentially interesting insight into the regulation of key HSC niche cell types, there are insufficient data to support all of the claims being made. Additional experiments and more precise writing could significantly strengthen this manuscript.

Major Comments
1. HO-1-deficient mice develop anemia. Can the authors rule out that changes in the HSC compartment do not occur as a consequence of the anemia? For example, does increased HSC cycling occur to produce more erythroid cells in order to compensate for the anemia?

Thank you for the valuable comment. We analyzed anemia in HO-1$^{-/-}$ mice and performed experiment to verify whether phenotype of LT-HSCs in HO-1$^{-/-}$ mice can be recapitulated by bleeding-induced anemia in HO-1$^{+/+}$ mice. The results are presented in new Fig. 9 in the manuscript.
When we analyzed 8-10 week old HO-1$^{-/-}$ mice we observed altered MCV and MCH blood parameters, that may indicate iron deficiency and microcytic anemia (Fig. 9A). However, the hemoglobin levels were not changed, while RBC levels where even higher in HO-1$^{-/-}$ mice (Fig. 9A), what suggests that young HO-1$^{-/-}$ mice can still compensate the iron deficiency.

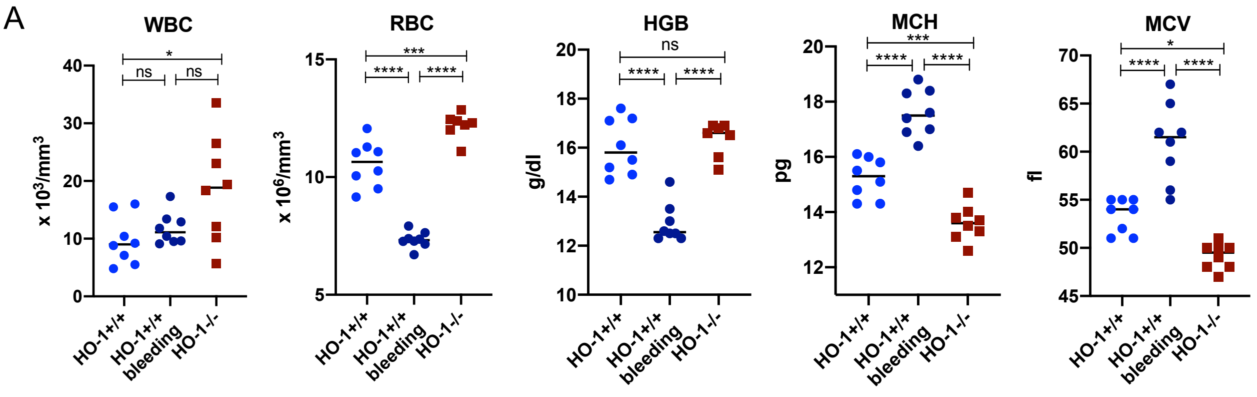

New **Figure 9**, panel A – included in the manuscript – blood morphology in young HO-1$^{-/-}$ mice.

Nevertheless, we serially bled HO1$^{+/+}$ mice – each third day, total 4 bleedings – and 3 days after last bleeding we assessed whether the bleeding-induced anemia can

recapitulate the LT-HSC phenotype. We analyzed the LT-HSC numbers, cell cycle, and global transcriptome by RNA-seq (all samples were sorted and processed simultaneously).

We confirmed that bleeding induces significant anemia in mice, as indicated by RBC and hemoglobin levels. However, the anemia did not affect the LT-HSC frequency and cell cycle (Fig. 9B). We did not observe any effect on MPPs, but found significant expansion of MEPs (Fig. 9B).

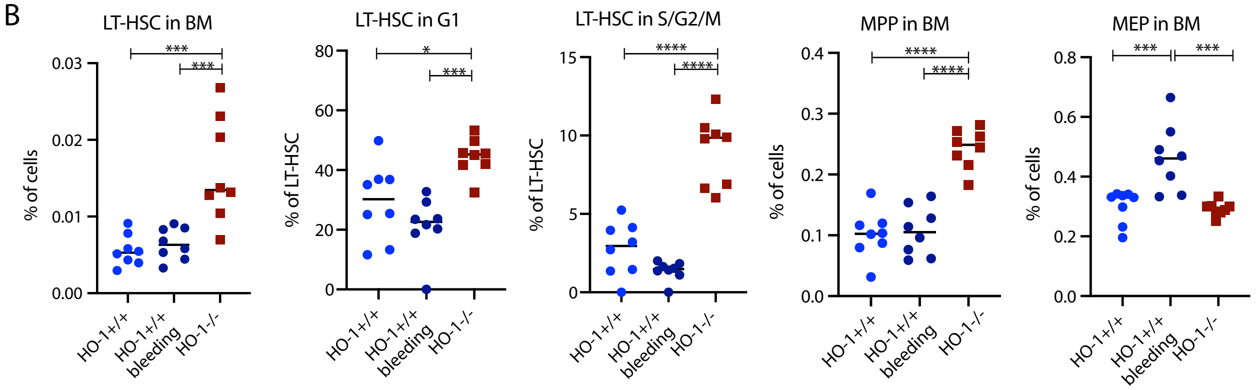

New **Figure 9**, panel B – included in the manuscript – effect of bleeding on HSC, MPP and MEP frequency.

Next, we checked if the bleeding affects the transcriptome of the LT-HSCs. Generally, we found only 45 differentially expressed genes (FDR <0.1) by bleeding (Fig. 9C,D), while consistently we observed over 1000 differentially expressed genes in HO-1$^{-/-}$ LT-HSCs (Fig. 9F). The performed GO analysis of the genes dysregulated by bleeding showed enrichment in general biological processes (Fig. 9E). When we compared the genes dysregulated by bleeding and dysregulated in HO-1$^{-/-}$ LT-HSCs we found that 25 of 45 genes affected by bleeding overlaps with the genes differentially expressed in HO-1$^{-/-}$ LT-HSCs. Nevertheless, PCA analysis on genes differentially expressed in both groups did not reveal similarity between LT-HSC from bled HO-1$^{+/+}$ mice and HO-1$^{-/-}$ mice.

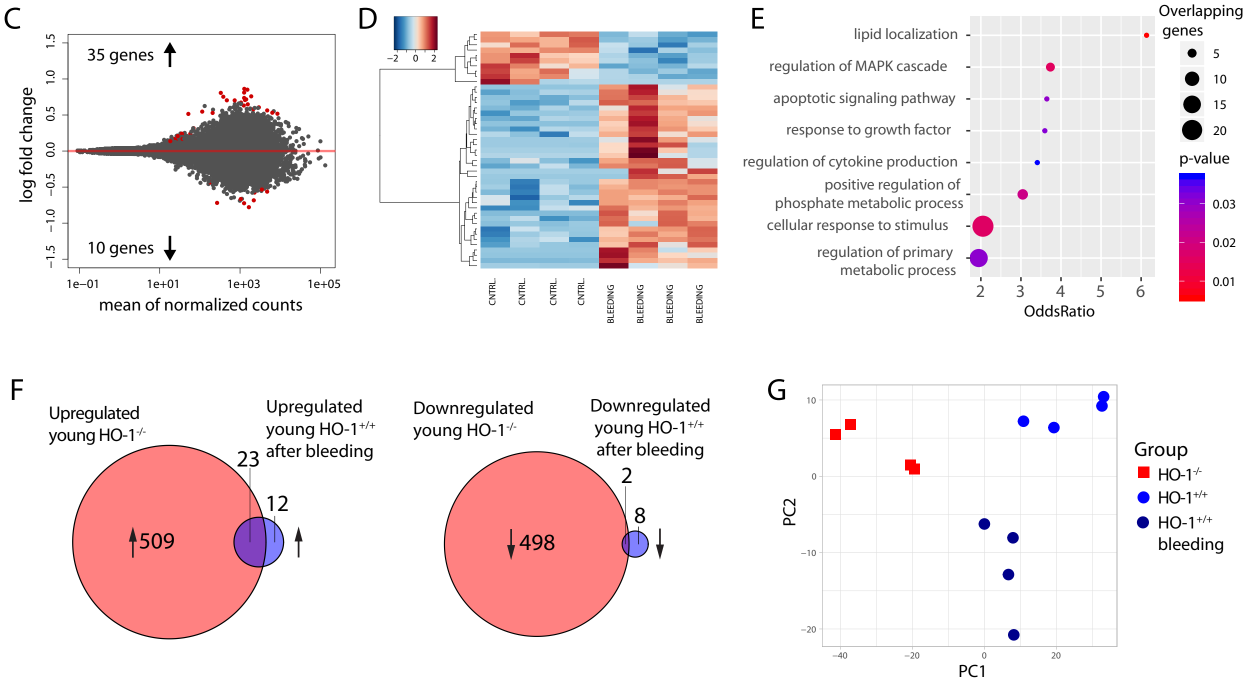

New **Figure 9**, panels C-G – included in the manuscript – effect of bleeding on LT-HSC transcriptome.

While this experiment showed that bleeding and anemia in our experimental scheme did not induce the phenotype typical for HO-1$^{-/-}$ LT-HSCs, we still cannot exclude that systemic effects in HO-1$^{-/-}$ mice affect LT-HSCs. Therefore, we rewrite the manuscript to present this possible interpretation.

2. A major conclusion of the study is that HSC maintenance requires HO-1 expression by ECs and/or CARs. It is surprising then that the authors only used the HO-1 conditional knockout mice to delete HO-1 in macrophages, but not in ECs and/or CARs. If the effects of HO-1 deficiency on HSCs are indeed mediated by ECs and CARs, then the phenotype should be recapitulated with conditional deletion of HO-1 in one or both of those populations.

We fully agree with Reviewer that conditional deletion in ECs or CARs would help to interpret the observed phenotype of LT-HSC in HO-1$^{-/-}$ mice. However, at the time the experiments were performed we did not have access to HO-1-flox mice. The LysM-Cre;HO-1$^{-/-}$ were breed obtained by our collaborators.
While obtaining the conditional knockouts would take significant amount of time, we performed alternative experiments based on *in-vitro* coculture of HSCs with mesenchymal stromal cells (MSCs), as explained in response to Reviewer 1 and presented in the manuscript as Fig 8. This experiment demonstrated that HSCs co-cultured with HO-1$^{-/-}$ MSCs gave rise to colonies with altered differentiation and kinetic of growth (please, see the response to the Reviewer 1, first comment).

3. The authors claim that wild-type HSCs transplanted into HO-1-deficient mice exhibit reduced long-term reconstituting activity and don't reconstitute secondary recipients (Fig.

6). However, HO-1-deficient ECs and CARs express reduced levels of Sdf1 (Fig. 2), which is a key factor required for HSC homing to the BM. Are these results explained by reduced homing/retention of HSCs in the BM after transplantation? Also, do HO-1 deficient mice have elevated numbers of HSCs in the spleen and peripheral blood?

We checked by flow cytometry whether the numbers of HSCs in blood and in spleen in HO-1 mice are altered. In our hands we were able to detected only single LKS $CD150^+CD48^-$ HSCs in ~0.8 ml collected blood. Nevertheless, we did not observe differences in LKS or LKS $CD150^+CD48^-$ frequency in peripheral blood between analyzed groups (Fig. R7).

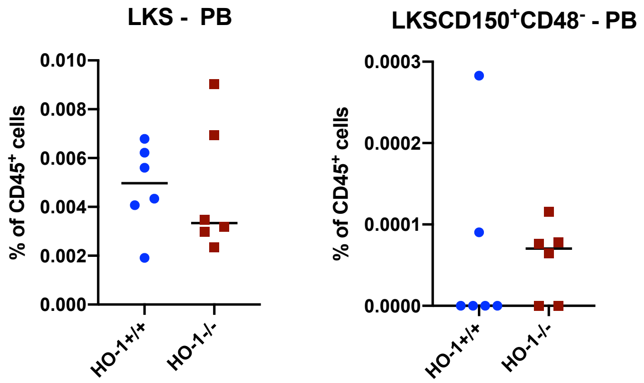

Figure R7 – not included in the manuscript – frequency of LKS and LKS $CD150^+CD48^-$ in peripheral blood.

We detected phenotypic LT-HSC (LKS $CD150^+CD48^-$) in the spleen in HO-$1^{+/+}$ mice, but their frequency as well as frequency of other progenitor populations were significantly diminished in HO-$1^{-/-}$ spleens (Fig. R8). However, it has to be noticed that spleens in young HO-$1^{-/-}$ mice are significantly enlarged but quickly become fibrotic. This dynamic process can likely affect the spleen niches, thus has to be taken into account when analyzing the HSC numbers in the spleen. Nevertheless, we did not observe any increased release of HSCs from the BM niche in HO-$1^{-/-}$ mice.

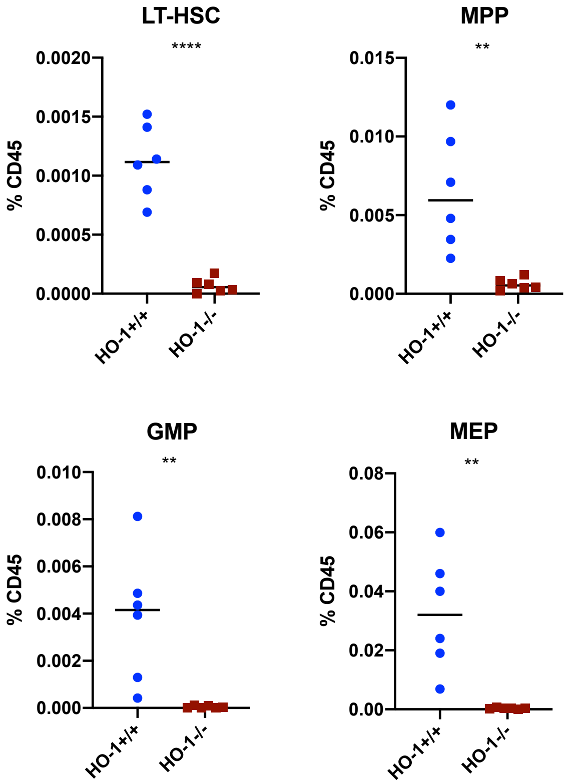

Figure R8 – not included in the manuscript – frequency of HSC and progenitors in spleen.

4. The authors claim that HSCs in HO-1-defcient mice exhibit premature aging. This is based on the HO-1-defcient HSCs exhibiting increased cycling, increased DNA damage, reduced reconstituting activity and some changes in gene expression. Do these changes really reflect premature aging? Perhaps the changes in DNA damage, reconstituting activity and gene expression mostly reflect the increased cycling of HSCs in HO-1 deficient mice? The authors should compare HSCs from HO-1-deficient mice to wild-type HSCs that have been driven into cycle.

We do agree with the Reviewer that such features like increased cycling, increased DNA damage, reduced reconstituting activity may not be enough to judge the aging of HSCs. Therefore, we performed the whole transcriptome analysis to analyze aging phenotype globally and found high similarity between aged HSC and young HSC from KO mice (Fig. 5 in the revised manuscript).
We agree that cycling can trigger premature aging of HSCs and can drive the exhaustion of the HSCs in HO-1 KO mice. We expected that bleeding would exert this effect as proposed by the Reviewer in the first point, as a natural physiological trigger of HSC proliferation, but it acted only on MEP level. While there are non-physiological methods to induce proliferation of HSC like, 5-FU, we think that such induction itself will alter the HSC transcriptome, making the transcriptome comparison of cycling HO-1$^{+/+}$ with HO-1$^{-/-}$ HSCs unreliable.

5. The most significant age-related change in HSCs is myeloid skewing (increased myeloid and reduced lymphoid differentiation). Do the authors observe myeloid biased differentiation from HSCs isolated from HO-1-deficient mice? If not, the authors should reconsider their claim of premature aging.

We addressed this issue in our manuscript:

"*In the present work, we did not use myeloid skewing as a marker of LT-HSC aging. We previously showed that the myeloid bias in HO-1-/- mice is linked to HO-1's role at the level of myelocytes [49]. Given that HO-1-deficiency causes the myeloid bias at the level of myelocytes, aging of LT-HSC in HO-1-/- animals could not be judged by increased output of mature myeloid cells.*"

Nevertheless, given that we cannot use the myeloid biased criteria, we rephrased our observation as premature exhaustion of HSCs, instead to premature aging according to the Reviewer criteria.

6. The "old" animals used in this study are typically 11-12 months old, and in some cases 18 months old. This is significantly younger than animals used in most HSC aging studies. In order to claim premature aging, the authors should use sufficiently old (>20-month-old) mice.

We use 12-month old animals in most of the experiments, and the 18-month old mice in RNA-seq experiment. We used 12-18 months old mice to check if there is early onset of aging process in young HSCs from HO-1 KO, what we called "premature exhaustion" process, but we are not claiming that they look liked aged (>20 month old) mice. According to the Reviewers advices we marked 12-month old animals as "mid-aged" in the revised manuscript.

7. Many of the differences within HSCs observed in HO-1-deficient mice disappear in 11-12-month-old mice (Fig. 3, 4). However, in Fig. 7 the authors claim that prolonged residence in a wild-type environment rescues transcriptional changes in HO-1-deficient HSCs. These experiments in Fig. 7 occur 48 weeks after transplant. How can the authors distinguish whether the rescue occurs because of the environment or because sufficient time has elapsed to allow for normalization of the transcriptional changes?

We fully agree with the Reviewer, that this is limitation of the assay, and we acknowledged this limitation in the manuscript. As the Reviewer noticed, aging itself affects expression of several genes, what probably leads to disappearing of some differences between HO-1 WT and HO-1 KO HSCs in old animals. We excluded these genes from analysis together with genes changed because of the transplantation itself (Fig. 7E in the revised manuscript). Although, some differences between WT and HO-1 KO HSCs disappeared with age, there are still significant number of genes changed in HSCs isolated from old HO-1 KO mice (Fig. 4B in the revised manuscript; 595 differentially regulated genes). Thus, our analysis aims to verify how many of these changes can be reversed by providing WT environment.

8. The authors claim to identify GCSFR expression as a new marker of aged HSCs. No validation of this marker is provided, and its overall relevance to this manuscript is questionable and distracting.

We agree with the Reviewer and removed these results from the manuscript. .

9. The metabolic data in Figure 5 do not provide significant mechanistic insight. If these data can't be expanded, I would suggest removing them entirely from the manuscript.

We agree with the Reviewer and moved these results to supplementary data.

10. The authors need to state their claims more carefully. For example, in the Abstract the authors state that "HSCs from young HO-1-/- animals lose... regenerative potential". This statement is misleading. Consistent with the authors' claims, the regenerative capacity of HO-1-deficient HSCs is significantly reduced. There is an important difference between reduced regenerative capacity and lost regenerative potential.

We fully agree with the Reviewer, and apologize for imprecise phrasing. This was corrected in the manuscript.

Minor Comments
1. What do the individual symbols represent in Figure 1H? The figure legend indicates that n for this experiment is 10-11, and yet there are only 3-4 data points.

For the real-time PCR experiment shown Fig. 1G the n = 10-11, however for the RNA-seq experiment shown in Fig. 1H n = 3-4, as shown by individual symbols. We clarified this in the Figure legend.

2. What do the red dots represent in Figure 3E?

These red dots represent the density of the dots on the plot – when the dots are overlapping red color indicate increased density.

3. Are the time points in Figure 3N shifted relative to the labels on the x-axis? Were samples taken at 4, 8, 12, and 16 weeks?

The samples are shifted to the labels on the x-axis, accordingly to exact time of analysis.

4. The Methods section is incomplete. a. The gamma H2Ax staining description is missing. b. The precise setup of transplantation experiments is difficult to interpret (both from the Methods section and schematics in figures). Are these always competitive transplants? Also, the irradiation dose listed in the Methods section differs from what is shown in the schematics.

a) We provided the exact protocol for γH2AX flow cytometry staining in the revised manuscript. b) All transplants are competitive, except the secondary transplant for the niche where we transplanted whole donor-derived BM. The irradiation doses were 9Gy

or 9.5Gy depending on experiment – we corrected it and clarified in the Materials and Methods section.

5. What is the reason for using 18-month-old animals for RNA seq experiments and 11-12 months old animals for other experiments assessing aging phenotype?

We wanted to use as old mice as possible for the RNA-seq experiment to define transcriptome signature of aged HSCs. The functional analyses were done in 11-12 month old mice, as these mice already showed age related changes. We analyzed whether young HO-1 KO mice showed early onset of aging changes, thus we used 11-12 month old individuals. While we understand that using older mice in whole experimental setting would be more consistent and clear, the aged HO-1 KO mice could not be obtained commercially and we are limited to our internal colony.

6. The manuscript has many typos and inconsistent labeling in Figures (LKS/KLS; days after transplant/weeks after transplant), which makes the text unclear and difficult to follow.

We apologize for the editing errors. We corrected them in the revised version of the article.

7. Negative controls for HO-1 staining should be included (i.e. cells and tissue from HO-1-deficient mice).

We added HO-1 KO control staining to the Supplement.

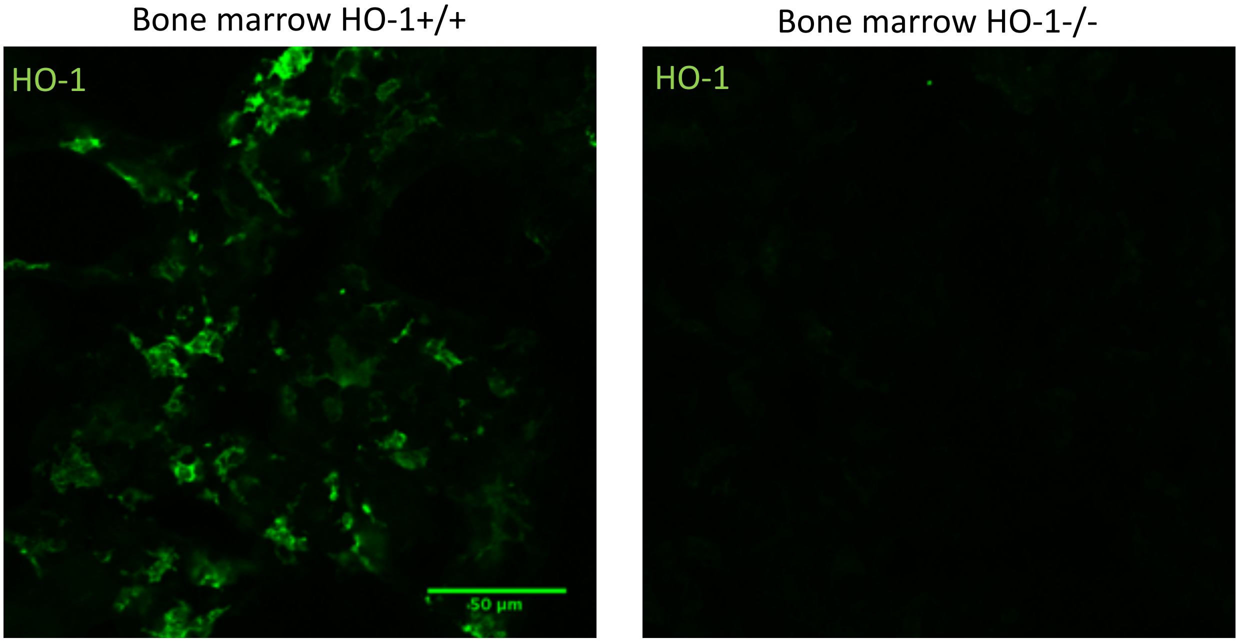

New **Appendix Figure S1.** Staining of bone marrow with HO-1 (SPA894) antibody in HO-$1^{+/+}$ and HO-$1^{-/-}$ mice.

2nd Editorial Decision 11 October 2019

Thank you for the submission of your revised manuscript to our editorial offices. We have now received the reports from the two referees that were asked to re-evaluate your study, you will find below. The original referee #2 declined to look into this again, but going through your point-by-point response, I consider his/her points as adequately addressed. As you will see, the two other referees support the publication of your study in EMBO reports. However, referee #1 (referee #3 from the submission to The EMBO Journal) has some remaining concerns and further suggestions to improve the manuscript, we ask you to address in a final revised version of the manuscript. Please also provide a point-by-point-response addressing these points.

Further, I have these editorial requests:

- I wonder if the title could be shortened:
Heme oxygenase-1 deficiency triggers exhaustion of hematopoietic stem cells

- Please format the manuscript as indicated in our instructions for authors:
http://www.embopress.org/page/journal/14693178/authorguide#manuscriptpreparation

The sections should be in this order:
Title page
Abstract
Introduction
Results
Discussion
Materials and Methods
Acknowledgements
Author contributions
Conflict of interest
References
Figure legends
Expanded View Figure legends

- Would it be possible to reduce the total number of main figures to 8. See also the comment 4 of referee #1. You can add one more EV figure.

- Please remove all the figures from the final manuscript text file. Figures should be uploaded as separate single files (or as part of the Appendix), and not included into the text file.

- Please separate the single microscopic images in Fig. 1A-D and EV1A-E by white dividing lines. Do not merge them together.

- It seems author Izabela Skulimowska is missing form the author contributions. Please check.

- It seems that call outs for Figs. 2E, 8G and 8H are missing in the manuscript text. Please check.

- Please add for all Dataset files a legend (describing the content) on the first TAB of the excel sheet.

- Please add page numbers to the Appendix file, and also to the table of contents (TOC).

- Please add uniform and clearly visible scale bars to all microscopic images.

- Part of the writing on the synopsis image is rather small looking at the image in the size it will be displayed online (see attached). Please provide a version with bigger fonts (in jpeg or tiff format with the exact width of 550 pixels and a height of not more than 400 pixels).

- Finally, please find attached a word file of the manuscript text (provided by our publisher) with changes we ask you to include in your final manuscript text, and some queries, we ask you to address. Please provide your final manuscript file with track changes, in order that we can see the

modifications done.

I look forward to seeing the final revised version of your manuscript when it is ready. Please let me know if you have questions regarding the revision.

REFEREE REPORTS

Referee #1 (referee #3 TEJ):

In this revised manuscript, Szade et al have done well to address the Reviewer comments. In general, the manuscript has been improved. However, some minor changes are required before publication.

1. From my previous review I stated: "The authors claim that wild-type HSCs transplanted into HO-1-deficient mice exhibit reduced long-term reconstituting activity and don't reconstitute secondary recipients (Fig. 6). However, HO-1-deficient ECs and CARs express reduced levels of Sdf1 (Fig. 2), which is a key factor required for HSC homing to the BM. Are these results explained by reduced homing/retention of HSCs in the BM after transplantation? Also, do HO-1 deficient mice have elevated numbers of HSCs in the spleen and peripheral blood?"

The authors addressed the second question, but I am still concerned that the diminished reconstitution in HO1-deficient recipients could be caused by diminished homing. The authors should transplant marked (eg. GFP+) hematopoietic stem and progenitor cells into irradiated HO-1-deficient and control mice and assess the abundance of GFP+ cells in the bone marrow 24 hours later.

2. I still disagree with the use of "premature aging" as terminology to describe the observed phenotypes. All use of this terminology should be replaced by more precise language. For example, stating that HO-1 deficient HSCs exhibit transcriptional profiles that more closely resemble aged HSCs rather than stating that HO1-Deficient HSCs exhibit premature aging.

3. In my interpretation, the new stromal co-culture data (Fig. 8) does not support the in vivo data. In vivo, HSCs from HO-1-deficient mice exhibit increased proliferation and premature exhaustion. If this is driven by defects in the niche cells, my expectation would be that HSCs co-cultured with HO-1-deficient stromal cells would form colonies more quickly (since the HSCs are more activated) and would give rise to more differentiated colonies (since the HSCs exhaust more quickly). Can the authors provide some clarity on the connection between the phenotypes in vitro or in vivo? In addition, I think the authors should remove Fig. 8I, as the cell surface phenotype of HSCs is not applicable to cultured cells.

4. I think the new bleeding data (Fig. 9) would be more appropriate as a supplementary figure.

Referee #2 (referee #1 TEJ):

The authors have adequately addressed prior concerns and modified the text to temper conclusions appropriately.

2nd Revision - authors' response 14 Novmeber 2019

We are grateful to the Reviewer for the positive evaluation of our resubmitted manuscript. Below please find our point-by-point response addressing the remaining concerns.

Ad.1

“*[From my previous review I stated: "The authors claim that wild-type HSCs transplanted into HO-1-deficient mice exhibit reduced long-term reconstituting activity and don't reconstitute secondary recipients (Fig. 6). However, HO-1-deficient ECs and CARs express reduced levels of Sdf1 (Fig. 2), which is a key factor required for HSC homing to the BM. Are these results explained by reduced homing/retention of HSCs in the BM after transplantation? Also, do HO-1 deficient mice have elevated numbers of HSCs in the spleen and peripheral blood?]*

The authors addressed the second question, but I am still concerned that the diminished reconstitution in HO1-deficient recipients could be caused by diminished homing. The authors should transplant marked (eg. GFP+) hematopoietic stem and progenitor cells into irradiated HO-1-deficient and control mice and assess the abundance of GFP+ cells in the bone marrow 24 hours later."

We agree with the Reviewer that impaired short-term homing may potentially contribute to the observed diminished reconstitution of HO-1 deficient recipients. To address this point we performed additional experiment addressing the short-term homing of GFP^+ hematopoietic stem and progenitor cells (HSPCs, defined as LKS phenotype) to irradiated HO-$1^{+/+}$ and HO-$1^{-/-}$ recipients. We did not observe any statistical difference in homing of HSPCs to HO-$1^{+/+}$ and HO-$1^{-/-}$ recipients. Concluding, the obtained data indicate that diminished reconstitution of HO-1 deficient recipients is not caused by impaired short-term homing.

These results were added and discussed in the manuscript and are presented in Fig. 6G,H. Additionally the Methods section was supplemented accordingly.

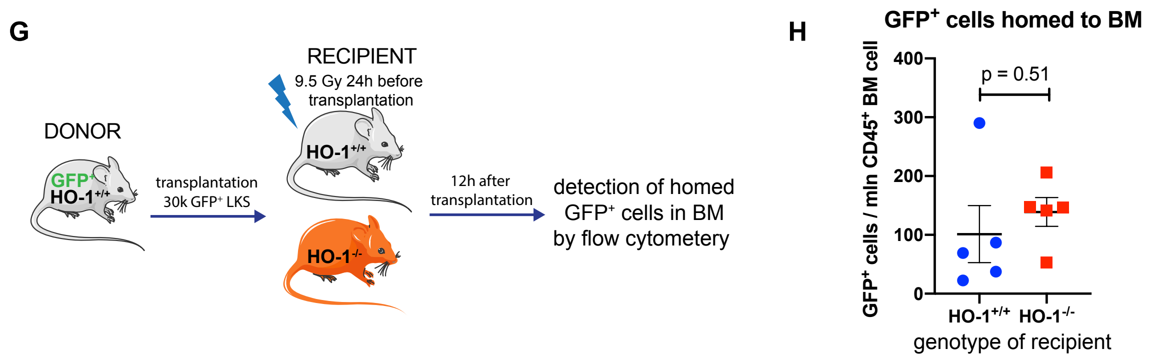

The results of the experiment assessing short-term homing of HSPCs to HO-$1^{+/+}$ and HO-$1^{-/-}$ recipients. Included in Fig. 6GH in the manuscript.

Ad. 2

"I still disagree with the use of "premature aging" as terminology to describe the observed phenotypes. All use of this terminology should be replaced by more precise language. For example, stating that HO-1 deficient HSCs exhibit transcriptional profiles that more closely resemble aged HSCs rather than stating that HO1-Deficient HSCs exhibit premature aging."

We removed all statements claiming the "premature aging" phenotype of HSCs and corrected accordingly to the Reviewer's suggestion.

Ad 3.

"In my interpretation, the new stromal co-culture data (Fig. 8) does not support the in vivo data. In vivo, HSCs from HO-1-deficient mice exhibit increased proliferation and premature exhaustion. If this is driven by defects in the niche cells, my expectation would be that HSCs co-cultured with HO-1-deficient stromal cells would form colonies more quickly (since the HSCs are more activated) and would give rise to more differentiated colonies (since the HSCs exhaust more quickly). Can the authors provide some clarity on the connection between the phenotypes in vitro or in vivo? In addition, I think the authors should remove Fig. 8I, as the cell surface phenotype of HSCs is not applicable to cultured cells. "

We agree that the data obtained from the performed in vitro assay do not fully concur with the in vivo phenotype. However, in vitro assays evaluating the function of HSCs clearly cannot reflect the complexity of the physiological niche of HSCs. Most of these assays, including the one used by us, are based solely on stromal cells. In contrast, our in vivo data suggest that both stromal cells and endothelial cells contribute to the niche-dependent HO-1 role on HSCs. Therefore, we believe that it is difficult to make any connection between phenotypes observed in the used in vitro assay with in vivo observations. We discussed these differences in in vitro and in vivo results in the revised manuscript.

According to Reviewer's suggestion, we removed Fig. 8I.

Ad. 4

"I think the new bleeding data (Fig. 9) would be more appropriate as a supplementary figure."

According to the Reviewer's suggestion, we changed Fig 9 to Expanded Figure 5 in revised version of the manuscript.

Acceptance 22 Novmeber 2019

I am very pleased to accept your manuscript for publication in the next available issue of EMBO reports. Thank you for your contribution to our journal.

© European Molecular Biology Organization

EMBO PRESS

YOU MUST COMPLETE ALL CELLS WITH A PINK BACKGROUND ➜

PLEASE NOTE THAT THIS CHECKLIST WILL BE PUBLISHED ALONGSIDE YOUR PAPER

Corresponding Author Name: Vladimir Benes
Journal Submitted to: EMBO Reports
Manuscript Number: EMBOR-2019-47895

Reporting Checklist For Life Sciences Articles (Rev. June 2017)

This checklist is used to ensure good reporting standards and to improve the reproducibility of published results. These guidelines are consistent with the Principles and Guidelines for Reporting Preclinical Research issued by the NIH in 2014. Please follow the journal's authorship guidelines in preparing your manuscript.

A- Figures
1. Data
The data shown in figures should satisfy the following conditions:

- the data were obtained and processed according to the field's best practice and are presented to reflect the results of the experiments in an accurate and unbiased manner.
- figure panels include only data points, measurements or observations that can be compared to each other in a scientifically meaningful way.
- graphs include clearly labeled error bars for independent experiments and sample sizes. Unless justified, error bars should not be shown for technical replicates.
- if n< 5, the individual data points from each experiment should be plotted and any statistical test employed should be justified
- Source Data should be included to report the data underlying graphs. Please follow the guidelines set out in the author ship guidelines on Data Presentation.

2. Captions

Each figure caption should contain the following information, for each panel where they are relevant:

- a specification of the experimental system investigated (eg cell line, species name).
- the assay(s) and method(s) used to carry out the reported observations and measurements
- an explicit mention of the biological and chemical entity(ies) that are being measured.
- an explicit mention of the biological and chemical entity(ies) that are altered/varied/perturbed in a controlled manner.

- the exact sample size (n) for each experimental group/condition, given as a number, not a range;
- a description of the sample collection allowing the reader to understand whether the samples represent technical or biological replicates (including how many animals, litters, cultures, etc.).
- a statement of how many times the experiment shown was independently replicated in the laboratory.
- definitions of statistical methods and measures:
  - common tests, such as t-test (please specify whether paired vs. unpaired), simple χ2 tests, Wilcoxon and Mann-Whitney tests, can be unambiguously identified by name only, but more complex techniques should be described in the methods section;
  - are tests one-sided or two-sided?
  - are there adjustments for multiple comparisons?
  - exact statistical test results, e.g., P values = x but not P values < x;
  - definition of 'center values' as median or average;
  - definition of error bars as s.d. or s.e.m.

Any descriptions too long for the figure legend should be included in the methods section and/or with the source data.

In the pink boxes below, please ensure that the answers to the following questions are reported in the manuscript itself. Every question should be answered. If the question is not relevant to your research, please write NA (non applicable). We encourage you to include a specific subsection in the methods section for statistics, reagents, animal models and human subjects.

USEFUL LINKS FOR COMPLETING THIS FORM

http://www.antibodypedia.com
http://1degreebio.org
http://www.equator-network.org/reporting-guidelines/improving-bioscience-research-repo
http://grants.nih.gov/grants/olaw/olaw.htm
http://www.mrc.ac.uk/Ourresearch/Ethicsresearchguidance/Useofanimals/index.htm
http://ClinicalTrials.gov
http://www.consort-statement.org
http://www.consort-statement.org/checklists/view/32-consort/66-title
http://www.equator-network.org/reporting-guidelines/reporting-recommendations-for-tum
http://datadryad.org
http://figshare.com
http://www.ncbi.nlm.nih.gov/gap
http://www.ebi.ac.uk/ega
http://biomodels.net/
http://biomodels.net/miriam/
http://jjj.biochem.sun.ac.za
http://oba.od.nih.gov/biosecurity/biosecurity_documents.html
http://www.selectagents.gov/

B- Statistics and general methods

Please fill out these boxes ➜ (Do not worry if you cannot see all your text once you press return)

1.a. How was the sample size chosen to ensure adequate power to detect a pre-specified effect size?	In the study the pre-specified size of the effect was not estimated before experiments. Most of the experiments involved animals, which number is limited accordign to obtained permission of local ethic commeete.
1.b. For animal studies, include a statement about sample size estimate even if no statistical methods were used.	Depending on the variation of the readout used, we aim to have 6-10 animals per group, where not possible, the minimal number of animal per group was 4.
2. Describe inclusion/exclusion criteria if samples or animals were excluded from the analysis. Were the criteria pre-established?	Any exlusion of data points were done accroding to outlier Grubbs test estimated with GraphPad software.
3. Were any steps taken to minimize the effects of subjective bias when allocating animals/samples to treatment (e.g. randomization procedure)? If yes, please describe.	All animals were randomly allocated to the group. We aimed to have all experiemental groups equally represented among one cage/litter of animals.
For animal studies, include a statement about randomization even if no randomization was used.	
4.a. Were any steps taken to minimize the effects of subjective bias during group allocation or/and when assessing results (e.g. blinding of the investigator)? If yes please describe.	The experimentators were not blinded during admnistartion of compounds to the animals . However, all sample processing, data collection and analysis was done with blinded manner.
4.b. For animal studies, include a statement about blinding even if no blinding was done	
5. For every figure, are statistical tests justified as appropriate?	We used the most appropriate test according the our best knowledge and the used test is reported in figures's legend. In general, for two groups comaprison we used un-pairad, two-tailed t test, for multigroup comparison wiht one variable 1way ANOVA with post-tests, and for multigroup compariosn with two variables we used 2-Way ANOVA with post-tests
Do the data meet the assumptions of the tests (e.g., normal distribution)? Describe any methods used to assess it.	We used GraphPad software for statistical analysis and estimation of normality and variance equality

Is there an estimate of variation within each group of data?	We report the SD or SEM as variance estimation on the graphs, or show the individual data points for direct estimation of variance
Is the variance similar between the groups that are being statistically compared?	We used GraphPad software for estimation of variance equality for comapred groups.

C- Reagents

6. To show that antibodies were profiled for use in the system under study (assay and species), provide a citation, catalog number and/or clone number, supplementary information or reference to an antibody validation profile. e.g., Antibodypedia (see link list at top right), 1DegreeBio (see link list at top right).	We reported the catalog numbers and/or clones of the reported antibodies. Most of the antibodies used are routinely tested and commonly used antibodies.
7. Identify the source of cell lines and report if they were recently authenticated (e.g., by STR profiling) and tested for mycoplasma contamination.	N/A No cell lines used in the study

* for all hyperlinks, please see the table at the top right of the document

D- Animal Models

8. Report species, strain, gender, age of animals and genetic modification status where applicable. Please detail housing and husbandry conditions and the source of animals.	We report age and strain of the mouse used for each experiemnt presented in the paper. All other experiments were performed in specific pathogen-free (SPF) conditions, with constant light/dark cycle and continuous monitoring of temperature and humidity. Mice were kept in groups ≤5 in the individually ventilated cages with food and water ad libitum.
9. For experiments involving live vertebrates, include a statement of compliance with ethical regulations and identify the committee(s) approving the experiments.	All animal procedures and experiments were performed in accordance with national and European legislations, after approval by the First or Second Local Ethical Committee on Animal Testing at the Jagiellonian University in Krakow (approval numbers: 56/2009, 113/2014, 120/2019).
10. We recommend consulting the ARRIVE guidelines (see link list at top right) (PLoS Biol. 8(6), e1000412, 2010) to ensure that other relevant aspects of animal studies are adequately reported. See author guidelines, under 'Reporting Guidelines'. See also: NIH (see link list at top right) and MRC (see link list at top right) recommendations. Please confirm compliance.	We confirm compliance.

E- Human Subjects

11. Identify the committee(s) approving the study protocol.	N/A
12. Include a statement confirming that informed consent was obtained from all subjects and that the experiments conformed to the principles set out in the WMA Declaration of Helsinki and the Department of Health and Human Services Belmont Report.	N/A
13. For publication of patient photos, include a statement confirming that consent to publish was obtained.	N/A
14. Report any restrictions on the availability (and/or on the use) of human data or samples.	N/A
15. Report the clinical trial registration number (at ClinicalTrials.gov or equivalent), where applicable.	N/A
16. For phase II and III randomized controlled trials, please refer to the CONSORT flow diagram (see link list at top right) and submit the CONSORT checklist (see link list at top right) with your submission. See author guidelines, under 'Reporting Guidelines'. Please confirm you have submitted this list.	N/A
17. For tumor marker prognostic studies, we recommend that you follow the REMARK reporting guidelines (see link list at top right). See author guidelines, under 'Reporting Guidelines'. Please confirm you have followed these guidelines.	N/A

F- Data Accessibility

18: Provide a "Data Availability" section at the end of the Materials & Methods, listing the accession codes for data generated in this study and deposited in a public database (e.g. RNA-Seq data: Gene Expression Omnibus GSE39462, Proteomics data: PRIDE PXD000208 etc.) Please refer to our author guidelines for 'Data Deposition'.<br><br>Data deposition in a public repository is mandatory for:<br>a. Protein, DNA and RNA sequences<br>b. Macromolecular structures<br>c. Crystallographic data for small molecules<br>d. Functional genomics data<br>e. Proteomics and molecular interactions	We provide "Data Availability" section and deposited the raw RNA-Seq data in SRA database. The description of files and accession are inlcuded in Bioproject entry The raw sequencing data from this publication have been deposited to the SRA database. Individual files are described can be accessed in BioProject database entry, accession: PRJNA562450.
19. Deposition is strongly recommended for any datasets that are central and integral to the study; please consider the journal's data policy. If no structured public repository exists for a given data type, we encourage the provision of datasets in the manuscript as a Supplementary Document (see author guidelines under 'Expanded View' or in unstructured repositories such as Dryad (see link list at top right) or Figshare (see link list at top right).	We deposited all output of analyzed RNA-seq data with the article as Expanded View Datasets
20. Access to human clinical and genomic datasets should be provided with as few restrictions as possible while respecting ethical obligations to the patients and relevant medical and legal issues. If practically possible and compatible with the individual consent agreement used in the study, such data should be deposited in one of the major public access-controlled repositories such as dbGAP (see link list at top right) or EGA (see link list at top right).	N/A
21. Computational models that are central and integral to a study should be shared without restrictions and provided in a machine-readable form. The relevant accession numbers or links should be provided. When possible, standardized format (SBML, CellML) should be used instead of scripts (e.g. MATLAB). Authors are strongly encouraged to follow the MIRIAM guidelines (see link list at top right) and deposit their model in a public database such as Biomodels (see link list at top right) or JWS Online (see link list at top right). If computer source code is provided with the paper, it should be deposited in a public repository or included in supplementary information.	We used commonly accepted R packages for the analysis and cited the published methods used for the bioinformatic analysis.

G- Dual use research of concern

22. Could your study fall under dual use research restrictions? Please check biosecurity documents (see link list at top right) and list of select agents and toxins (APHIS/CDC) (see link list at top right). According to our biosecurity guidelines, provide a statement only if it could.	Our study does not fall under dual use research restrictions.
